# Design and Synthesis of 2‐En‐4‐Ynedioate Compounds as Novel and Potential Antifungal Agents

**DOI:** 10.1155/bmri/5560073

**Published:** 2026-06-23

**Authors:** Mohadese Mohammadi, Soroush Sardari, Parisa Azerang, Parviz Rashidi Ranjbar

**Affiliations:** ^1^ Department of Chemistry, College of Science, University of Tehran, Tehran, Iran, ut.ac.ir; ^2^ Clinical Research Department, Pasteur Institute of Iran, Tehran, Iran, pasteur.ac.ir; ^3^ Drug Design and Bioinformatics Unit, Medical Biotechnology Department, Biotechnology Research Center, Pasteur Institute of Iran, Tehran, Iran, pasteur.ac.ir

**Keywords:** antifungal drugs, *in silico*, squalene epoxidase, synthesis

## Abstract

In this investigation, we focused on generating innovative antifungal agents inspired by terbinafine, a prominent allylamine that inhibits squalene epoxidase and is a crucial enzyme in the biosynthesis of fungal ergosterol. Utilizing computational methods such as molecular docking and molecular dynamics (MD) simulations, we designed and synthesized a collection of enyne derivatives featuring aromatic side chains. The choice of these compounds was influenced by their anticipated interactions with squalene epoxidase, informed by comprehensive in silico assessments. The synthesized derivatives were analyzed using ^1^H‐NMR, ^13^C‐NMR, and mass spectrometry techniques. Among the synthesized series, Compound 2b (1,6‐bis[(4‐methylphenyl)methyl] (2E)‐hex‐2‐en‐4‐ynedioate) exhibited potent antifungal activity, demonstrating a minimum inhibitory concentration (MIC) of 0.0391–0.7176 *μ*M/mL against *Candida albicans*, *Saccharomyces cerevisiae*, and *Aspergillus niger*. These findings highlight the promising strategy of applying these novel compounds, based on the structural design of terbinafine, as viable candidates for antifungal treatment. This approach demonstrates a significant advancement in the design of antifungal agents targeting squalene epoxidase and suggests promising avenues for further development.

## 1. Introduction

In recent years, despite remarkable progress in pharmaceutical research and the creation of antifungal medications, both superficial and invasive fungal infections continue to be a significant source of illness and death, particularly among immunocompromised individuals [1]. The frequency of opportunistic fungal infections, especially those attributed to *Candida* species, has been on the rise [2]. Currently, 17 distinct species of *Candida* with pathogenic potential have been recognized, with *Candida albicans* being the most common culprit behind fungal infections [3]. At‐risk groups encompass patients undergoing chemotherapy, individuals with diabetes, those receiving prolonged antibiotic therapies, organ transplant recipients, and older adults with dental prosthetics, all of whom face heightened vulnerability due to the invasive characteristics of these infections [4–8]. Throughout the last 40 years, numerous antifungal agents have been developed to address these infections. These medications, which include azoles, allylamines, polyenes, and echinocandins, function by inhibiting key enzymes involved in the ergosterol biosynthesis pathway or by disrupting the integrity of fungal cell membranes [9–13]. However, the emergence of multidrug‐resistant fungal strains and the side effects associated with these drugs have limited their clinical efficacy [14, 15]. To address these challenges, researchers have explored dual/multitarget drugs that can simultaneously inhibit multiple enzymes, thereby reducing the likelihood of resistance and side effects [16, 17]. Azole antifungals, which inhibit CYP51 during ergosterol biosynthesis, are commonly used for treating invasive fungal infections, particularly in patients with hematologic malignancies [18]. Terbinafine, an allylamine antifungal agent, inhibits squalene epoxidase (SQLE), a key enzyme in ergosterol biosynthesis, and is highly effective against dermatophytes and certain yeasts [19]. Given the potential of SQLE inhibitors in antifungal therapy, our study focuses on designing and synthesizing new antifungal compounds based on the structural framework of terbinafine. Previous studies suggest that the presence of alkene and alkyne groups can enhance the antifungal activity of these compounds [20–26]. Utilizing molecular docking and in silico ADMET analysis, we have identified a series of enyne derivatives with aromatic side chains as promising candidates [27–33].

Several heteroaryl and aryl‐substituted scaffolds have been reported to possess broad‐spectrum antimicrobial properties, demonstrating the value of aromatic substitution patterns for tuning biological activity [34, 35]. These findings support our design strategy of introducing diverse aromatic side chains onto the enyne/ester backbone to optimize SQLE inhibition. Preliminary results indicate that Compound 2b demonstrates significant antifungal activity, highlighting its potential as a leading compound for further development.

## 2. Material and Methods

### 2.1. Homology Modeling

Terbinafine exerts its antifungal effects by inhibiting the enzyme SQLE, playing a crucial role in ergosterol biosynthesis in fungi. For this study, SQLE from *C. albicans* was selected as the target enzyme. The enzyme sequence was retrieved from the GenBank database in FASTA format. Due to the absence of a crystallized three‐dimensional structure for the target enzyme, homology modeling was employed to construct a model. To build a reliable model, three‐dimensional structures with high identity were identified using the BLAST algorithm on the NCBI web server. The structure with 100% identity and an *E*‐value of 0 was chosen as the template [36]. Additionally, the fold recognition method was applied to compare various three‐dimensional structure prediction methods [37]. This approach involved substituting the target sequence directly into the main SQLE enzyme template. The initial sequence was searched using the I‐TASSER server.

### 2.2. Molecular Docking Study

#### 2.2.1. Preparation of Ligands

All ligands were searched for similarity using the Tanimoto index (> 70%) with databases such as ZINC15, PubChem, ChEBI, Enhanced NCI, Sigma, and ChemSpider. Terbinafine was chosen as the lead molecule for the search. Retrieved database structures were converted to 3D and optimized using HyperChem (HyperChem Professional 7.51, Hypercube, Inc., Gainesville, Florida, United States). Pharmacophore searching was conducted using the MOE application, with the enyne group selected as the pharmacophore.

#### 2.2.2. Preparation of Protein

Considering the mechanism of action of terbinafine, which inhibits SQLE, this enzyme was selected as the target protein. The final model of the enzyme obtained from the homology modeling procedure was subjected to docking analysis.

#### 2.2.3. Docking

PyRx Version 0.8 was used for docking studies [38]. Ligands were converted to PDBQT format, and the receptor was prepared similarly. The following amino acids were chosen as the active site: Phe420, Leu340, Pro430, Val240, Cys416, Phe402, Tyr77, Leu249, Leu398, His447, Leu434, and Leu394. A grid box was set to encompass these amino acids. Docking parameters included a central box of dimensions 79.5 × 64.6 × 67.0 Å and grid boxes of 24.5 × 29.5 × 25.1 Å. The lowest binding energy conformations were selected.

### 2.3. Molecular Dynamics Simulations

Compounds with the best docking poses were selected for molecular dynamics simulations to study ligand interactions with the active sites of the SQLE enzyme. Simulations were performed for 100 ns in explicit water using the AMBER software package. AmberTools was used to generate starting files for all ligands [39]. Force field parameters were calculated using the general AMBER force field (GAFF) and the RESP charge model. The AMBERff14SB force field was used for the protein. The TIP3P water model was employed to solvate the compounds, and ions were added for neutrality. Periodic boundary conditions were applied, and all simulations were carried out using the parallel version of PMEMD in Amber22. The system was minimized and equilibrated with Langevin dynamics, managing temperature and pressure.

### 2.4. Chemistry

#### 2.4.1. Instrumentation and Chemicals

All reagents and solvents were purchased from Sigma‐Aldrich (St. Louis, Missouri, United States) unless otherwise stated. Silica gel 60 (Merck, 70–230 mesh) was used for column chromatography. ^1^H‐NMR (500 MHz) and ^13^C‐NMR (125 MHz) were recorded on a Bruker DRX‐500; chemical shifts are reported in parts per million relative to TMS; DMSO‐d_6_ was used as the solvent, as indicated. Mass spectra (EI/ESI) were recorded on an Agilent 5975C mass spectrometer. TLC was performed on Merck silica gel 60 F_254_ and visualized under UV light (254 nm). Antifungal assays were performed using minimum inhibitory concentration (MIC) assays according to CLSI M27‐A3 (yeasts) and M38‐A2 (filamentous fungi) [40, 41].

#### 2.4.2. The Synthesis Route of Propiolic Acid

**Scheme 1:**
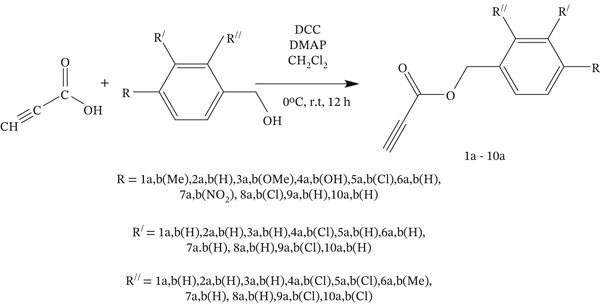
Esterification of propiolic acid to yield benzyl esters (1a–10a).

The intermediates of 1a and 1b were synthesized with some modifications. First, 5 mmol of propiolic acid was added to 0.5 mmol of DMAP and 7.5 mmol of benzyl alcohol in 5 mL of dichloromethane at 0°C. Then, 5 mmol of triethylamine was added to increase the nucleophilic character of propiolic acid, and 6 mmol of DCC was also added. The reaction was followed at 0°C for 12 h, and then, the reaction was brought to room temperature. After that, it was diluted with dichloromethane to reach neutral pH and washed with water. Then, it was dehydrated by Na_2_SO_4_ and dried by vacuum distillation. With the help of Celite, it was filtered until DCU (dicyclohexylurea) was produced as a by‐product and separated. The pure ester product was isolated by column chromatography. Eventually, the pure ester product was isolated with a petroleum ether and ethyl acetate solvent ratio of 4:1 (Scheme 1) [42].

#### 2.4.3. Coupling Reaction of Aryl Ester (1b–10b)

**Scheme 2:**
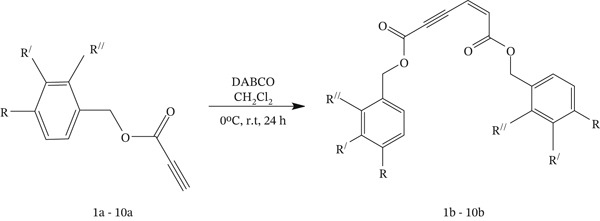
Coupling of aryl esters to form enyne derivatives (1b–10b).

Five millimoles of the primary synthesis ester (1a–10a) was added in 10 mL of dichloromethane in a 50‐mL flask at 0°C and stirred. Then, 0.05 mmol of DABCO was added to it, and stirring was continued for about 24 h. After the completion of the reaction, the product was separated by column chromatography (Scheme 2) [43].

### 2.5. Biology

#### 2.5.1. Antifungal Activity by Broth Microdilution Method

All of the synthesized compounds were tested for their antifungal activity, and the MIC was determined by the serial dilution technique according to the CLSI standard method [44]. *Aspergillus fumigatus*, as a fungal strain, and *C. albicans* (ATCC 10231) and *Saccharomyces cerevisiae* (PTCC 5052) were used for the in vitro studies. Terbinafine was used as a standard drug.

#### 2.5.2. Ergosterol Binding Assay

According to the study by Arthington‐Skaggs et al. [45], the determination of the compounds on ergosterol biosynthesis in *C. albicans* was done. In the applied method, the amount of intracellular ergosterol in *Candida* cells was measured. Briefly, 200 *μ*L of *Candida* cells (0.5 McFarland) was inoculated into 20 mL of SDB with and without 1 × MIC, 0.5 × MIC, and 0.25 × MIC of synthesis compounds and incubated at 35°C for 48 h in the shaker incubator. The cells were collected by centrifuging at 5000 rpm for 5 min and then washed with distilled water. Then, it was dried at room temperature, and the weight of the cell was determined. Next, 3 mL of 25% KOH was added and vortexed for 1 min. Then, the suspension was transferred to glass tubes, incubated at 90°C for 1 h in a water bath, and allowed to cool to room temperature. Sterol was extracted by adding 1 mL of distilled water and 3 mL of heptane–nitrogen. The mixture was vigorously vortexed for 3 min and then allowed to rest for 30 min. The heptane layer was transferred to clean glass tubes and kept at 20°C for 24 h. Before analysis, 1 mL of sterol standard value was diluted five times in 100% ethanol and then examined by UV. We analyzed the results based on the amount of ergosterol reduction compared to the control (without treatment).

#### 2.5.3. Cellular Morphology Analysis Using SEM and TEM

The morphological changes of the susceptible strains of *C. albicans* ATCC 18804 were assessed using SEM and TEM following treatment with potent Compounds 10a and 2b according to a previous report with some modifications [46]. Treated and untreated (control) cells were analyzed by SEM and TEM.

After the incubation period (*C. albicans*: 2, 4, and 6 h with the Derivatives 10a and 2b at concentrations of MIC, 2 MIC, and 1/2 MIC) at 35°C, for primary fixation, fungal cells were treated, and untreated cells were washed three times with PBS (3000 rpm for 5 min). After washing, it was treated with 0.2% glutaraldehyde at 37°C for 1 h. Subsequently, it was washed three times with PBS under the mentioned conditions, and the sediment was dissolved in 50 *μ*L of the last wash and spread on special slides. After drying, using 10%, 30%, 60%, 70%, 90% and 100% ethanol, the samples were dehydrated and prepared for imaging. At the end, they were coated with gold/platinum and observed with the SEM and TEM microscopes.

## 3. Results

### 3.1. Synthesis

^1^H‐NMR, ^13^C‐NMR, and mass spectrometry of all synthetic compounds are listed in the attachment. NMR spectroscopy was performed on a Varian INOVA 500 MHz device, and mass spectrometry was carried out using a 5975C device manufactured by Agilent Technologies. Table 1 showcases the chemical structures of the synthesized compounds, divided into esters (1a–10a).

**Table 1:** Structures of synthesized compounds containing ester (1a–10a).

1a	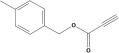	6a	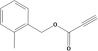
2a	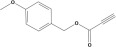	7a	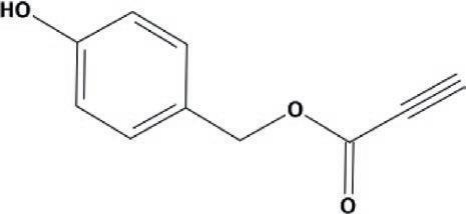
3a	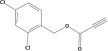	8a	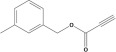
4a	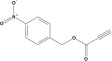	9a	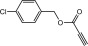
5a	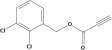	10a	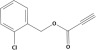

Table 2 presents the chemical structures of synthesized compounds containing the enyne moiety (1b–10b). These compounds are characterized by the presence of both an alkene and an alkyne within the same molecule.

**Table 2:** Structures of synthesized compounds containing enyne (1b–10b).

1b	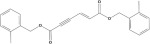	6b	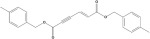
2b	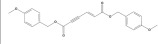	7b	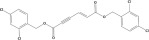
3b	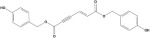	8b	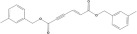
4b	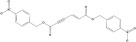	9b	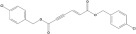
5b	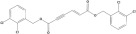	10b	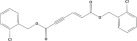

### 3.2. MIC Results

The MIC test for synthesized compounds was performed on *A. fumigatus*, *S. cerevisiae*, and *C. albicans*, and its results can be seen in Table 3.

**Table 3:** Best MIC results of synthesized compounds against *C. albicans*, *S. cerevisiae*, and *A. fumigatus*.

Compound	*C. albicans* (ATCC 10231) MIC (*μ*g/mL)	*S. cerevisiae* (PTCC 5052) MIC (*μ*g/mL)	*A. fumigatus* (ATCC 16424) MIC (*μ*g/mL)	Remarks
1a	15.62	62.5	62.5	Good activity
2a	15.62	31.25	62.5	Good activity
3a	23.44	62.5	125	Good activity
4a	31.25	125	125	Good activity
9a	7.6	7.6	7.6	Excellent activity
10a	7.6	7.6	7.6	Excellent activity
2b	250	31.25	250	Notable among enyne
9b	1000	1000	125	Potent in some strains
10b	1000	1000	125	Potent in some strains
Terbinafine (std.)	25	10	< 7.6	Reference compound
DMSO	10% *v*/*v*	10%	10%	Negative control

*Note:* Full MIC dataset (all synthesized Compounds 1a–10a and 1b–10b) is provided in Table S1 in the Supporting Information.

According to the MIC results, Compounds 1a, 2a, 3a, and 4a had good activity. From the screening results, Compounds 9a and 10a showed excellent activity against all strains (Table 3). Remaining compounds showed good to moderate activity against all strains.

### 3.3. SEM and TEM Results

In order to investigate the effects of the designed compounds on the cell surface and also to investigate the morphology of the fungus, SEM was used as one of the appropriate techniques to discover the possible mechanism of antimicrobial action of the best compounds (2a, 2b, and 10a). In SEM analysis, the morphological changes of *C. albicans* treated with compounds after exposure are examined. The exposure was evaluated during 24 h. Untreated *C. albicans* cells in Figure 1a had a smooth and intact surface. In fungi treated with terbinafine at concentrations of 0.5× MIC, the cells were wrinkled and perforated (Figure 1b).

Figure 1Image obtained using scanning electron microscope (SEM) and TEM. (a) *C. albicans* cells without drug treatment (24‐h culture). (b) *C. albicans* treated with terbinafine at 1/2 MIC concentration. (c) *C. albicans* treated with Compound 2a at 1/2 MIC concentration. (d) *C. albicans* treated with Compound 4a at 1/2 MIC concentration. (e) *C. albicans* treated with Compound 2b at 1/2 MIC concentration. (f) *C. albicans* treated with Compound 10a at 1/2 MIC concentration.

**Figure:**
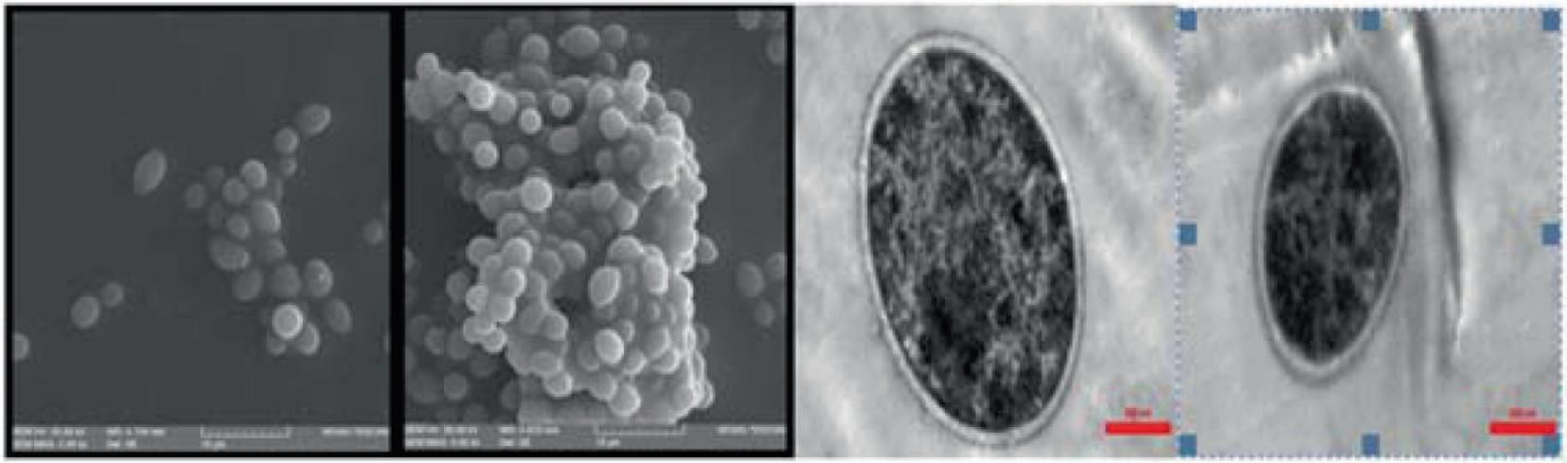
(a)

**Figure:**
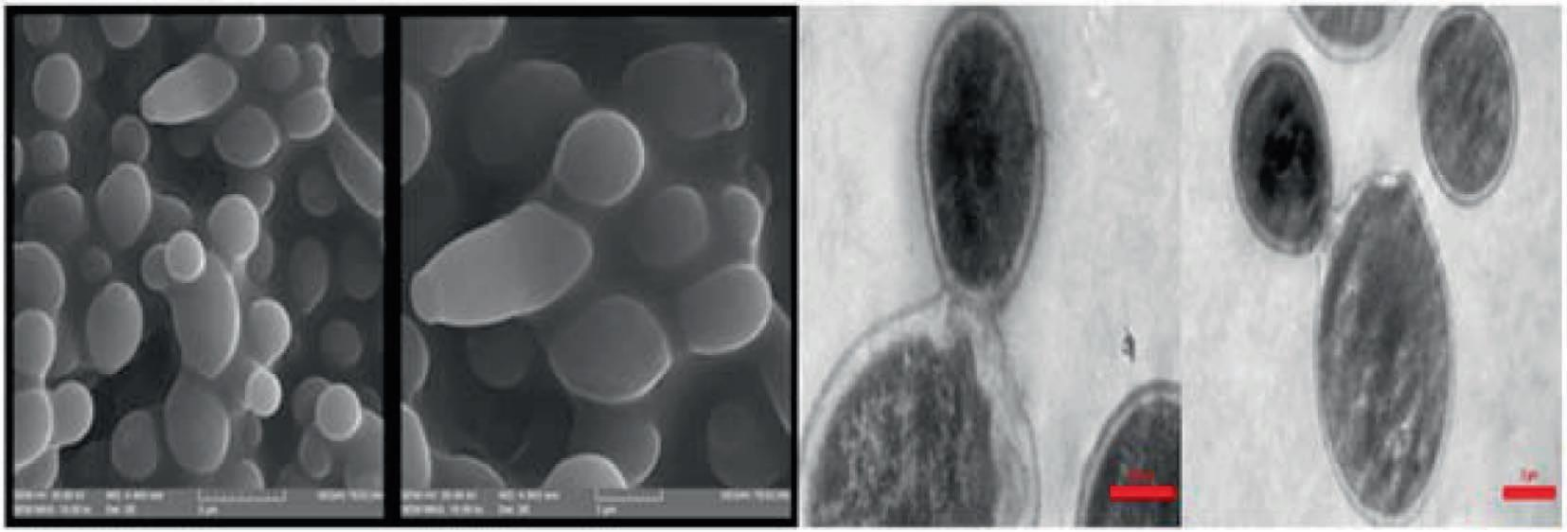
(b)

**Figure:**
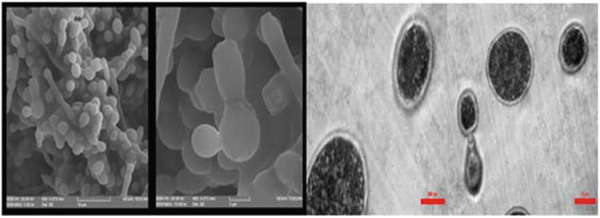
(c)

**Figure:**
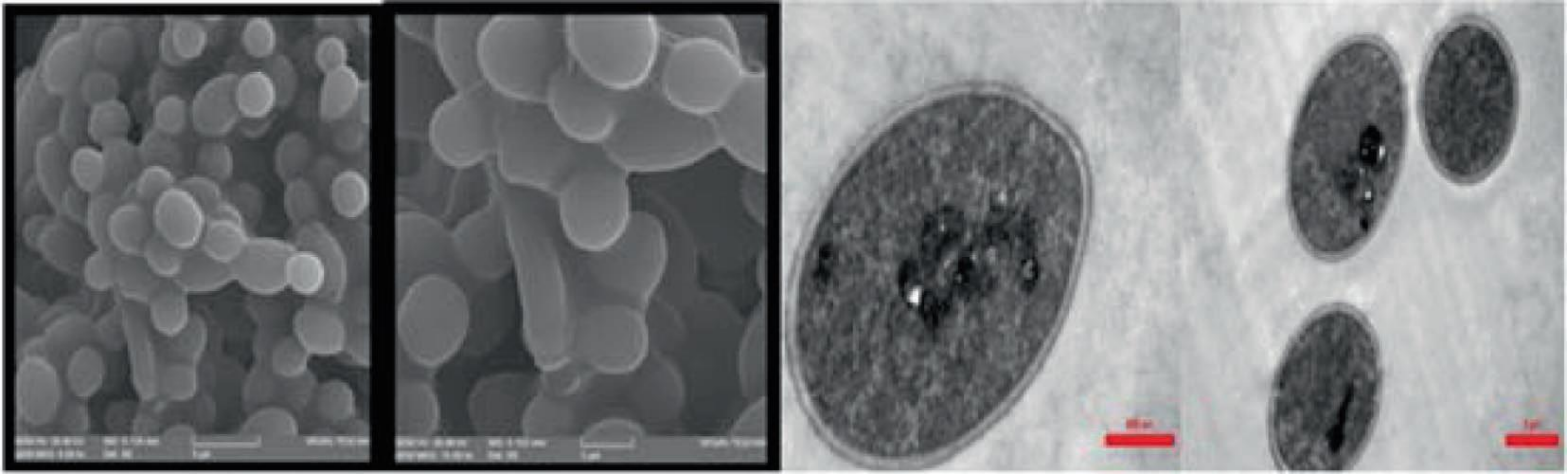
(d)

**Figure:**
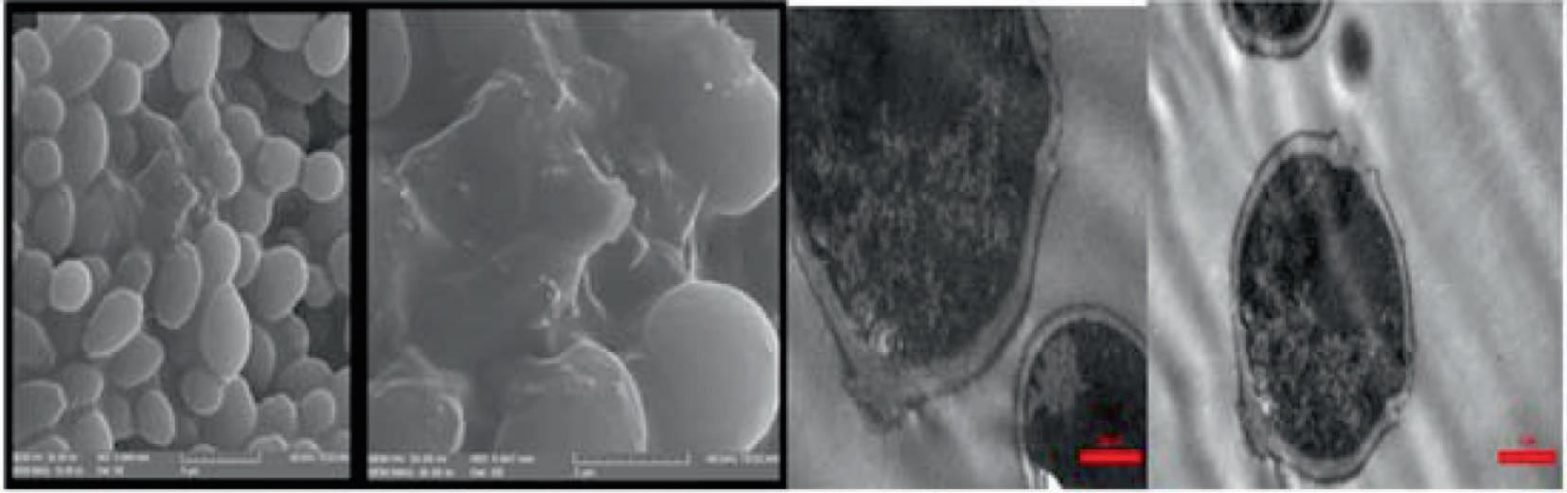
(e)

**Figure:**
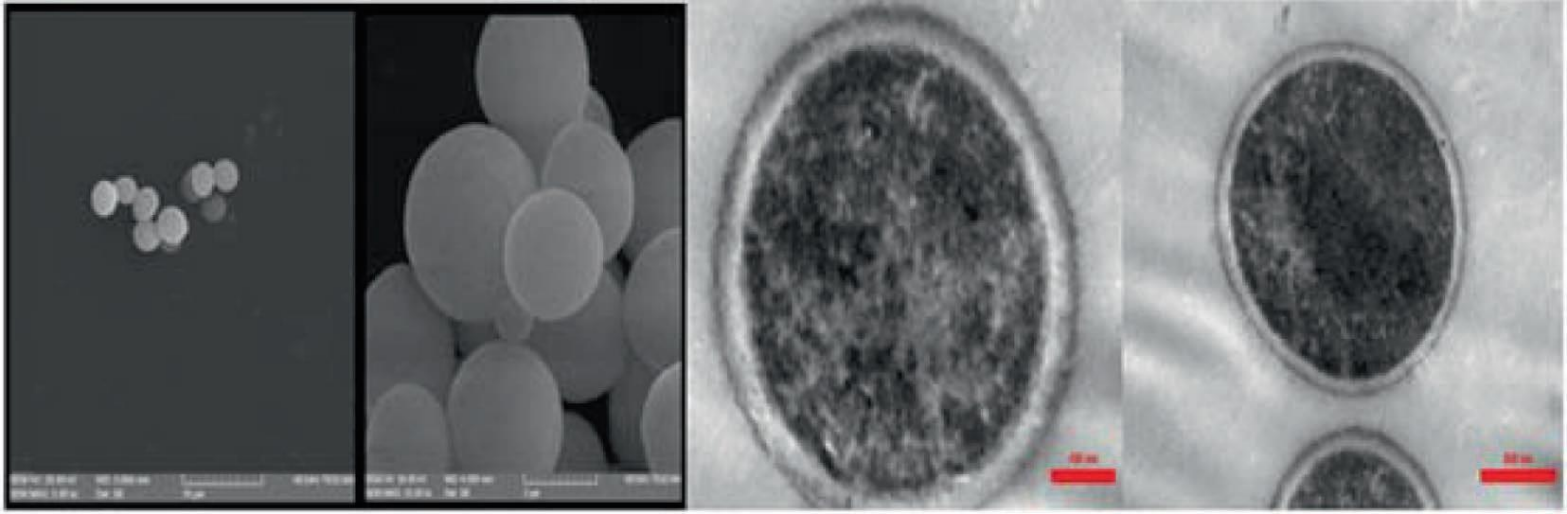
(f)

Normal *Candida* cells (Figure 1a) were round and spherical and also had smooth surfaces and clusters of grapes with uniform size and distribution. But in cells treated with Compounds 10a, 2a, 2b, and 4a at 0.5× MIC (Figures 1c, 1d, and 1e, the morphology of the surfaces is changed, some cells are stuck to each other, and some parts of the cells are broken. In surface defects, cell division into a ring of scars is noticeable at the tip of the blastoconidial cell and the bud that comes out from the opposite tip of the cell. The blastoconidial septum looks fragile. The true hyphal filament is also remarkable.

In the pictures related to SEM, terbinafine in the vicinity of *Candida* (concentration 1/2 MIC), Figure 1b shows the formation of dark colored vacuoles in the cell after exposure to the drug. Also, in Figure 1c related to Compound 2a, the presence of dark and numerous granules shows the vacuole after exposure to the compound. Also, Figure 1d shows the formation of dark colored vacuoles inside the cytoplasm after exposure to the drug.

### 3.4. Ergosterol Measurement

The method of Arthington‐Skaggs et al. was used to determine the effect of compounds on ergosterol biosynthesis in *Candida* cells. With this method, the amount of intracellular ergosterol in *Candida* cells was measured and compared. The results were checked by a spectrophotometer. Then, the results were analyzed based on the amount of ergosterol reduction compared to the control (without treatment) (Figure 2).

Figure 2Diagram of ergosterol level. (a) A 24‐h culture of *Candida* fungus without drug (b) 2 MIC, MIC, and 1/2 MIC: terbinafine; (c) 2 MIC, MIC, and 1/2 MIC: 10a; (d) 2 MIC, MIC, and 1/2 MIC: 2b.

**Figure:**
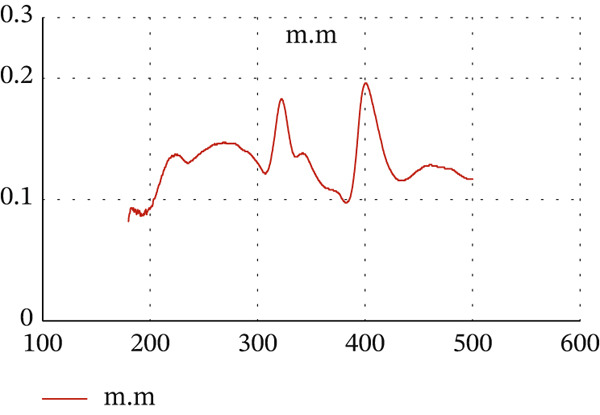
(a)

**Figure:**
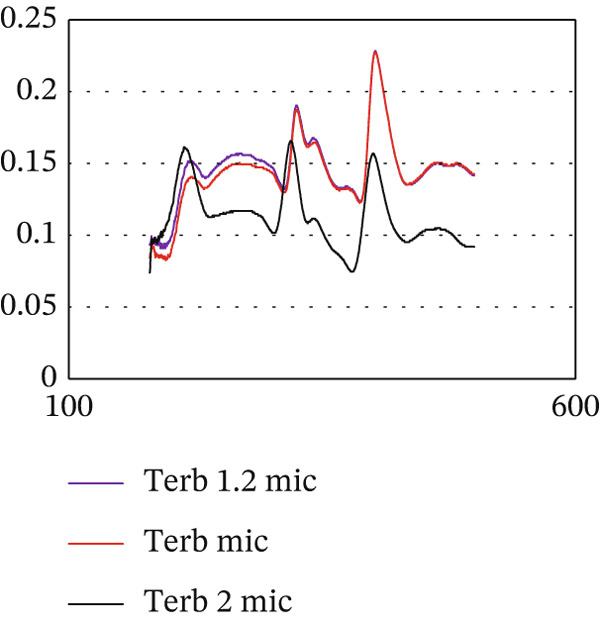
(b)

**Figure:**
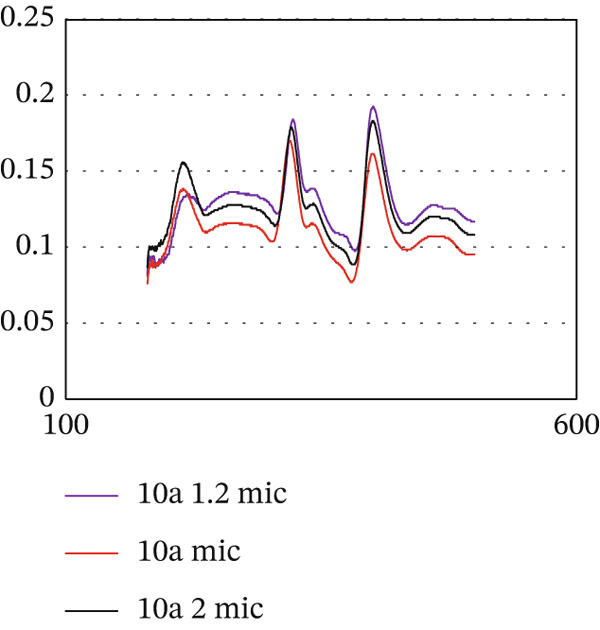
(c)

**Figure:**
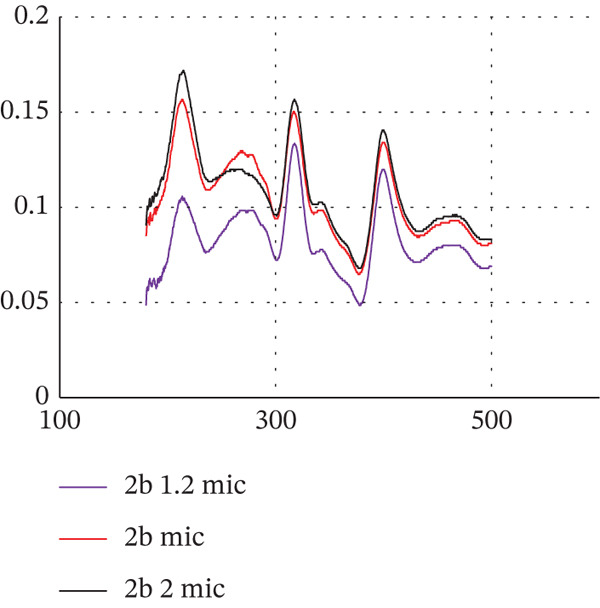
(d)

### 3.5. Homology Modeling

For the sequence section, the FASTA format was selected and used in the Swiss model server for modeling homology.

The fold recognition method was also used to compare different methods of preparing the three‐dimensional structure of the sequence (folding detection). In this method, instead of template amino acids, the desired sequence is directly substituted for the main protein. Search the initial sequence on the I‐TASSER server, and the results can be summarized in Table 4 for 10 models performed.

**Table 4:** Fold recognition result for the desired sequence.

Rank	PDB code	TM score	RMSD	Cov.
1	6c6nA	0.897	0.51	0.901
2	5dbjE	0.740	3.20	0.829
3	3nixA	0.707	3.08	0.792
4	3ataqA	0.705	3.57	0.821
5	5wgrA	0.697	4.35	0.843
6	3e1tA	0.697	3.72	0.796
7	5fn0A	0.691	3.70	0.804
8	1dodA	0.688	3.02	0.770
9	6bzaA	0.687	3.73	0.794
10	6ainA	0.685	3.07	0.770

*Note:* Finally, with the studies performed and the results of both methods, Model 1 (6c6nA) was selected as the protein in this study.

### 3.6. Molecular Docking and Molecular Dynamics Analysis

The docking results (Table 5) indicate that the enyne derivatives exhibit various binding affinities toward SQLE, with docking scores ranging from −12.1 to −9.3. Notably, terbinafine, as a reference ligand, has a docking score of −9.3. Compound 5b shows the highest binding affinity with a docking score of −12.1. This compound, with two hydroxyl groups on the phenyl rings, may form strong hydrogen bonds with the active site of the enzyme, enhancing its binding affinity. Compound 3b follows with a docking score of −11.1. The presence of methoxy groups likely contributes to its interaction with SQLE, albeit less effectively than the hydroxyl groups in 5b. Compounds 1b, 7b, 4b, 2b, and 8b have docking scores ranging from −10.3 to −10. These compounds exhibit moderate binding affinities, with various substituents such as methyl, dichloro, and chloro groups influencing their binding interactions. The variance in scores within this group suggests that while these substituents offer some advantage, they do not provide as strong an interaction as hydroxyl or methoxy groups. Compounds 8, 10b, 10, and 12 have docking scores from −9.9 to −9.3, comparable to terbinafine. The structural similarities in the backbone of these compounds might offer a similar interaction profile as terbinafine, but with slight modifications that do not significantly enhance or diminish their binding affinities.

**Table 5:** A 2D demonstration of selected ligands and docking outcomes (in kcal/mol).

Compound	IUPAC name	MW (g/mol)	Docking score (kcal/mol)	*Δ* *G*_bind (MMGBSA) (kcal/mol)	Remarks
5b	Bis[(4‐hydroxyphenyl)methyl] hex‐2‐en‐4‐ynedioate	352.34	−12.1	−36.6 ± 4.0	Highest docking score
3b	1,6‐Bis[(4‐methoxyphenyl)methyl] (2E)‐hex‐2‐en‐4‐ynedioate	380.4	−11.1	−34.5 ± 4.4	Strong H‐bonding
2b	1,6‐Bis[(4‐methylphenyl)methyl] (2E)‐hex‐2‐en‐4‐ynedioate	348.4	−10.3	−21.1 ± 2.4	Lead in some assays
Terbinafine	Reference	277.4	−9.3	Reference	Standard

The root‐mean‐square deviation (RMSD) of the C*α* atoms in the protein backbones was measured during a 100 ns MD simulation (Figure 3a). The RMSD examination showed dissimilarities in the backbone motion of the protein in each ligand′s manifestation. The CA atoms variations in the presence of all compounds except 1a and 3b (Figure 3a) follow a similar configuration, with the variability of about two and three orders of magnitude instead of four and five in 1a and 3b. According to the RMSD plots, the binding of all ligands except 1a and 3b restricts the motion of the protein.

Figure 3(a) The root‐mean‐square deviation (RMSD) of the C*α* atoms in the protein backbones was measured during a 100 ns MD simulation. (b) The root‐mean‐square fluctuation (RMSF) shows the variation of every single CA atom relative to the average position for the protein in the presence of ligands.

**Figure:**
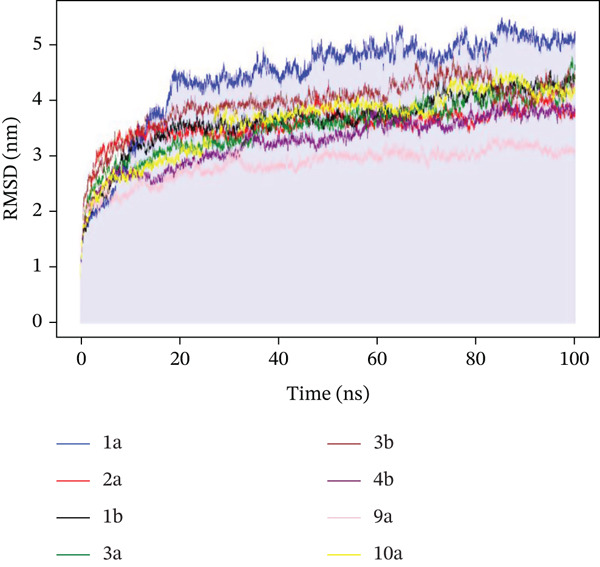
(a)

**Figure:**
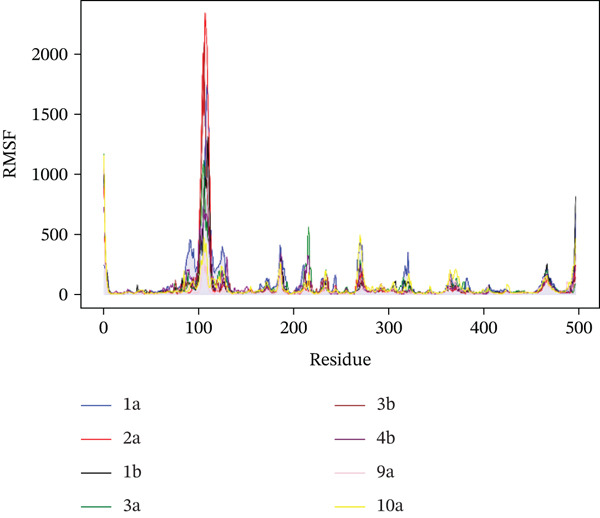
(b)

The root‐mean‐square fluctuation (RMSF) shows the variation of every single CA atom relative to the average position for the protein in the presence of ligands, and it is outlined in Figure 3b. According to the figure, the substantial fluctuations accrued in the residue range 80–120 when we have Compounds 1a and 2a in the active site of the protein. Huge fluctuations occur in the residue range of 425–450 of the enzymes.

Conformational clustering was conducted on the molecular dynamics trajectories of the previously described ligand–protein complexes to select representative conformations for further evaluation. Clustering was carried out using the *k*‐means algorithm and the RMSD of residues, with a sampling frame of 10 frames. We considered the densest cluster centers, which are more stable, as the representative binding mode conformations (Figures 4a, 4b, 4c, 4d, 4e, 4f, 4g, and 4h). Analysis of the protein active site discloses two primary interactions that influence the accommodation of ligand in the active site: a hydrophobic “clamp” that provides affinity and a hydrogen bonding network that determines binding modes.

Figure 4Binding modes of squalene epoxidase with representative structures of Compounds (a) 1a, (b) 2a, (c) 1b, (d) 3a, (e) 3b, (f) 5b, (g) 9a, and (h) 10a from clustering analysis.

**Figure:**
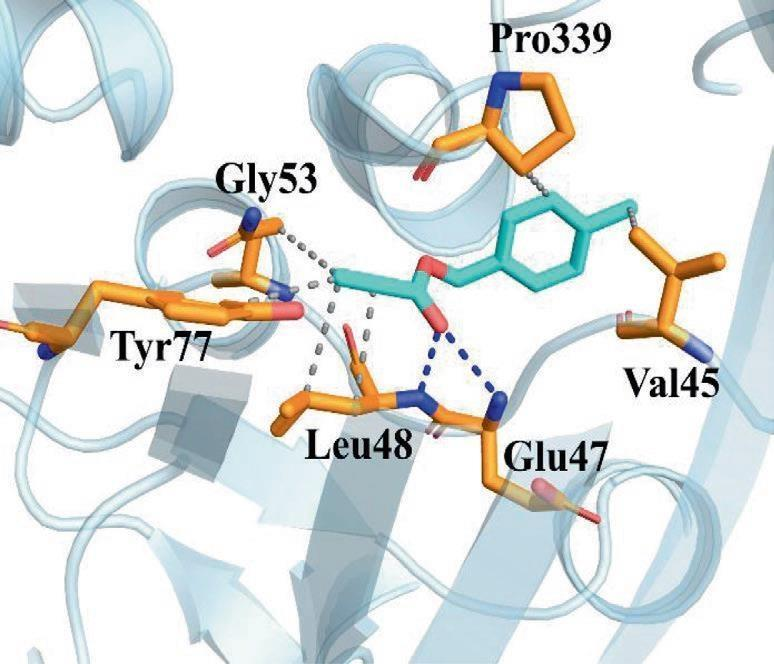
(a)

**Figure:**
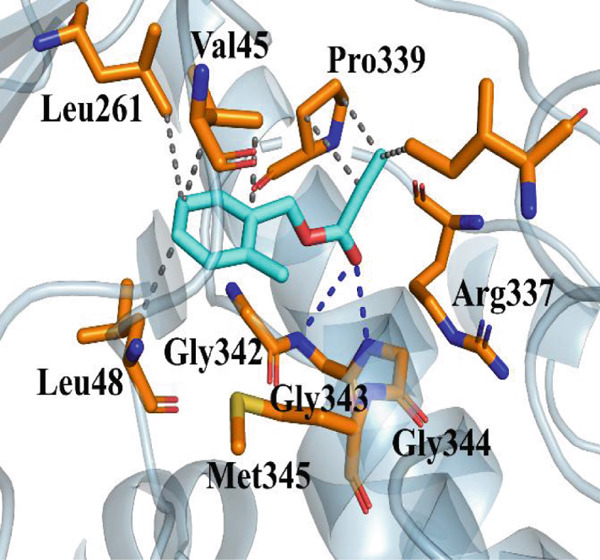
(b)

**Figure:**
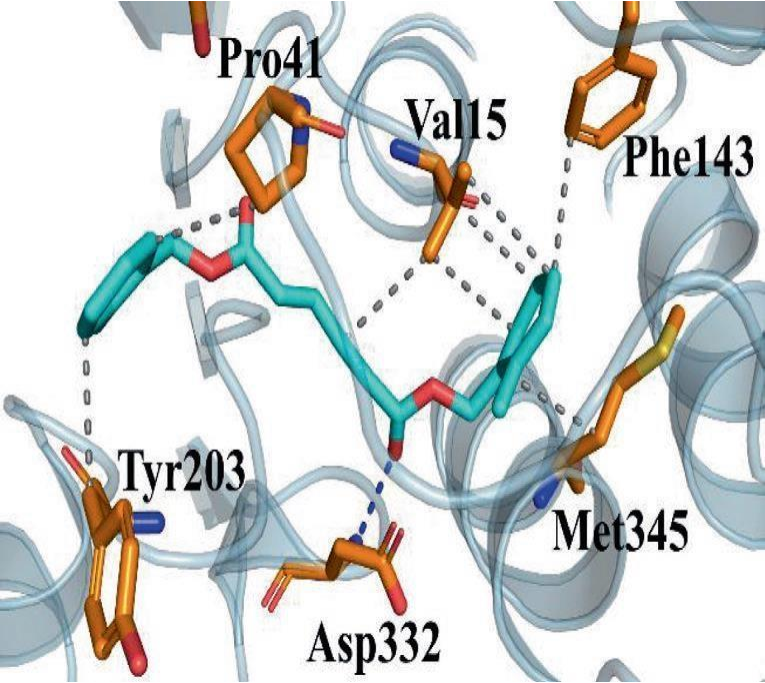
(c)

**Figure:**
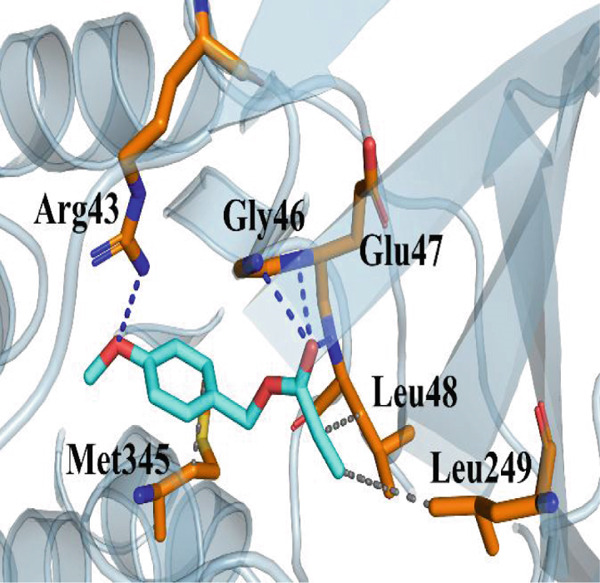
(d)

**Figure:**
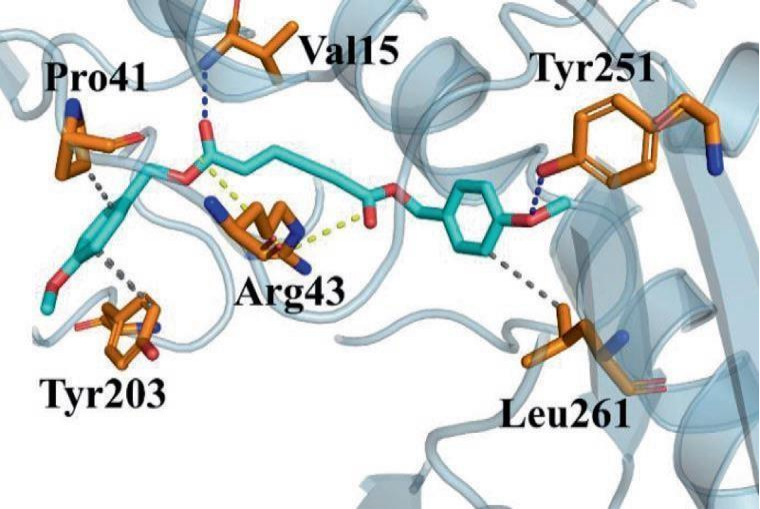
(e)

**Figure:**
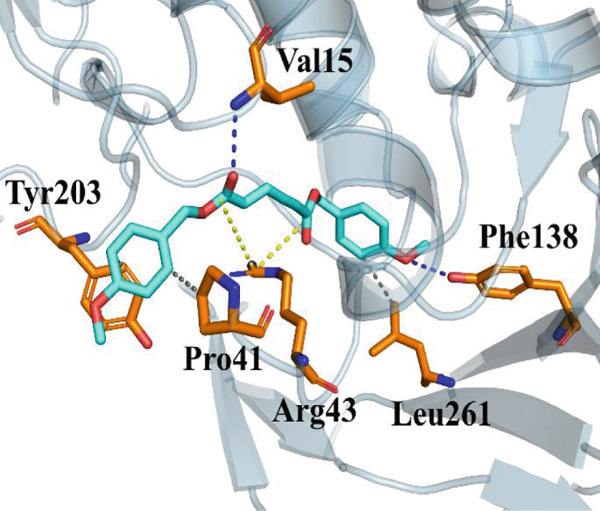
(f)

**Figure:**
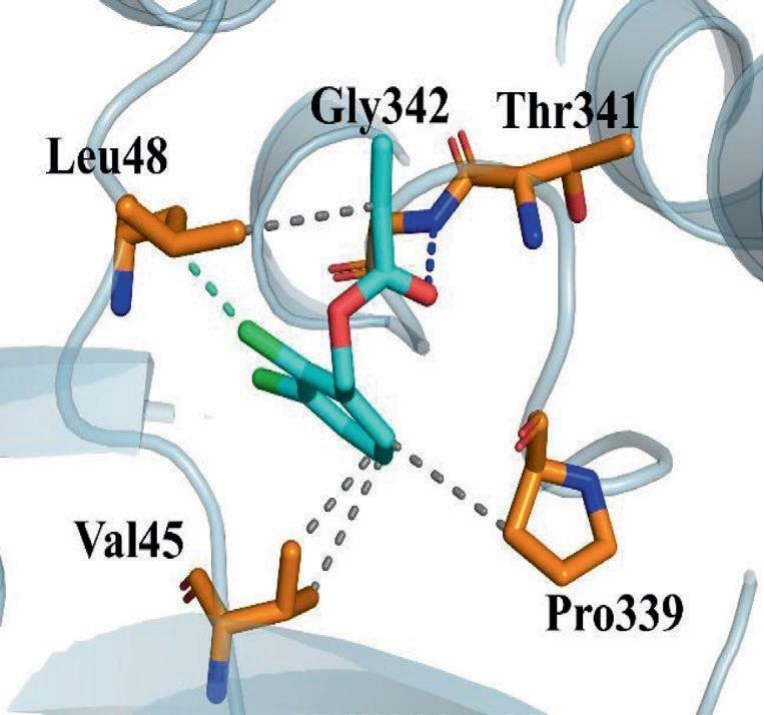
(g)

**Figure:**
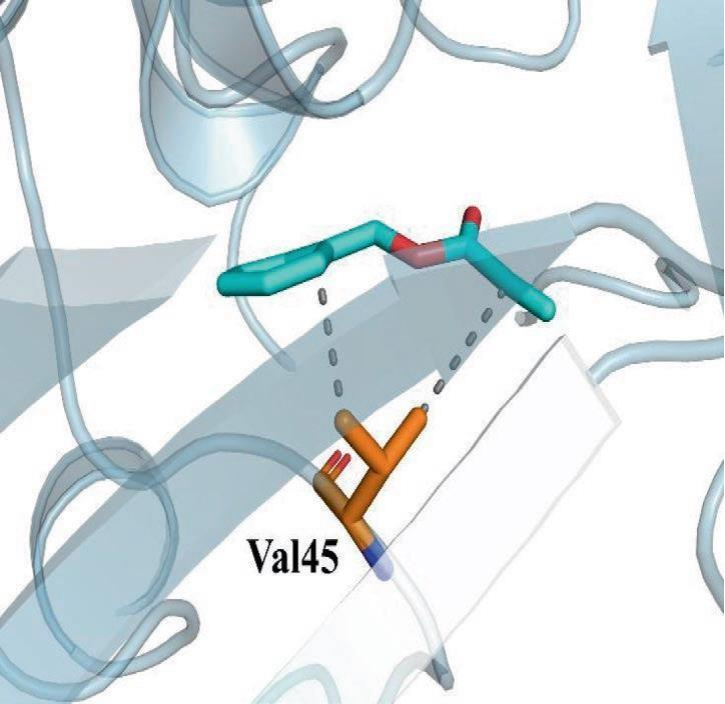
(h)

As presented in Figure 4a,b, when comparing the clustering figures for Compounds 1a and 2a, a notable difference is observed in their binding orientations. In Compound 1a, the enyne moiety is oriented toward Leu48, but in Compound 2a, the ring structure is oriented toward Leu48. This difference in orientation can be attributed to the structural variations between the two compounds, specifically the ortho substitution of a methyl group in Compound 2a. The presence of the methyl group in the ortho position of Compound 2a introduces steric hindrance. This bulky group prevents the ester part of the molecule from approaching Glu47 effectively. In Compound 1a, the ester oxygen is positioned to form hydrogen bonds with Glu47. This interaction is significant and influences the orientation of the molecule, allowing the enyne moiety to face Leu48. For Compound 2a, the methyl group hinders this interaction with Glu47. As a result, the compound reorients itself to avoid steric clashes, flipping the molecule so that the ring structure faces Leu48 instead. The flipping of Compound 2a facilitates the formation of hydrogen bonds with Gly342 and Gly343. This new orientation allows the molecule to stabilize within the binding pocket through interactions that are otherwise hindered in Compound 1a due to the lack of steric hindrance from the methyl group. Figure 4d shows the key interactions and orientation of Compound 3a within the active site of the protein. The notable feature of Compound 3a is the presence of a methoxy group at the para position. Similar to Compounds 1a and 2a, Compound 3a interacts with Leu48, Leu249, and Met345 through hydrophobic contacts. This indicates that van der Waals (vdW) interactions play a significant role in the binding of all three compounds. The interaction of Gly46 and Gly47 with Compound 3a is reminiscent of the hydrogen bonding observed in Compound 1a with Glu47. The presence of the methoxy group at the para position in Compound 3a introduces a different steric and electronic environment compared to Compounds 1a and 2a. The methoxy group can participate in additional dipole interactions and may influence the overall binding orientation. However, when the binding energy of these compounds is calculated, it can be observed that Compounds 1a and 3a have close binding energies to each other and are obviously better than Compound 2a (Table S2).

This implies that the introduction of a bulky group, like methyl, in the ring, especially at the ortho position, can significantly change its potency toward the enzyme. According to Figure 4g, the presence of two chlorine atoms at the ortho and meta positions in 9a introduces significant steric and electronic effects that stabilize the binding orientation. Compound 9a forms hydrogen bonds with Gly342 and Thr341 and interacts hydrophobically with Leu48, Val45, and Pro339. The orientation of Compound 9a shows that the Cl groups are positioned to interact favorably within the binding pocket, enhancing vdW interactions. However, the single chlorine atom at the ortho position in 10a reduces steric hindrance compared to Compound 9a and shows hydrophobic interactions with Val45 (Figure 4h). Unbolting to make hydrogen bonds in the active site of the protein, as well as having Cl groups at the ortho position of this compound, significantly reduces the binding energy. Figure 4c shows the clustering representative structure of Compound 1b within the binding site of the protein. This compound forms hydrogen bonds with Tyr203, interacting electrostatically with Asp332; forms hydrophobic interactions with Phe143, Val15, and Pro41; and engages in hydrophobic interactions, similar to other compounds, with Met345. Compound 1b shows a slightly lower binding affinity than 2a, indicating that the added bulk does not enhance binding but rather might introduce steric clashes or less optimal interactions. Figure 4e,f shows the clustering representative structure of Compounds 3b and 5b, respectively, within the binding site of the protein. Compound 3b forms hydrogen bonds with Tyr203, electrostatically interacting with Arg43, engaging in *π*–*π* interactions with Tyr251, which provides hydrophobic interactions with Leu261, Pro41, and Val15. Compound 3b has a binding energy of −23.6 kcal/mol, the best among all studied compounds. Compound 1b, which has a methyl group in the ortho position, causes steric hindrance and is less favorable for hydrogen bonding interactions, while Compound 3b introduces a methoxy group in the para position, which enhances dipole interactions and stabilizes the binding orientation.

### 3.7. Hydrogen Bond Analysis

To evaluate the type and strength of hydrogen bonds between 1a, 2a, 1b, 3a, 3b, 4b, 9a, and 10a and the protein, MD simulation of ligand–enzyme was accomplished during the 100 ns trajectory period. We used the H‐bond module of AmberTools22 to check the hydrogen bonding profiles between the chosen ligands and the enzymes. As default values, the angle and H‐bonding distance threshold were set to 135 ^′^ and 3.0 Å, respectively.

**Figure 5:**
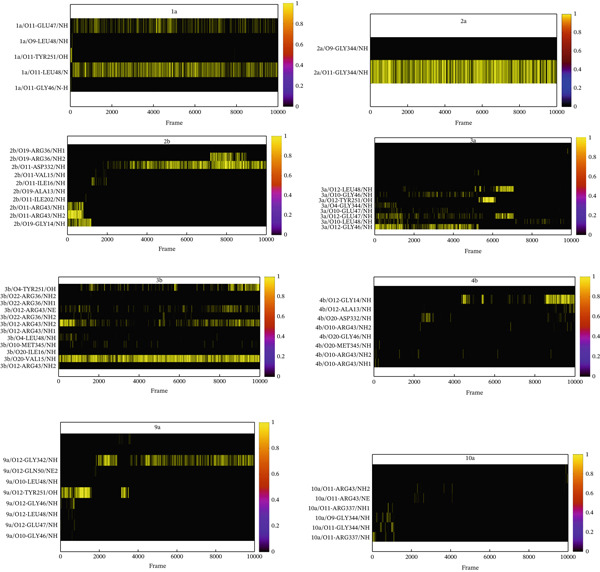
Hydrogen bond analysis for Compounds 1a, 2a, 2b, 3a, 3b, 4b, 9a, and 10a.

Figure 5 demonstrates the hydrogen bonding occupancy plots versus time for all aforementioned ligands. The hydrogen bonds occurred in more frame scale from 0 to 1 in terms of occupancy. Compound 3b, with the highest number of hydrogen bonds and the highest occupancy, can be a good reference for other ligands to be evaluated in this regard. This compound has three H‐bonds with the highest occupancies during MD simulation involving the ‐NH group of Val15 and the ‐NH2 group of Arg43 with O22 and O12 atoms of the ligand. The other most potent ligands, such as 9a and 1a, have a different pattern of H‐bonding involving Gly 46, Gly47, and Leu48 with O10, O11, and O12 of the ligands. This finding indicates that Arg43 and Leu 48 play a crucial role in accommodating the ligand in the middle of the active site of the enzyme.

### 3.8. Binding Free Energy and Energy Decomposition Analysis

The binding affinity of selected ligands was investigated using the MM‐PB/GBSA method to estimate binding free energy. To calculate the MM‐GB/PBSA binding energy, 1000 snapshots of the MD trajectory were taken. According to the definitions in Equations 1–4, Table S2 shows the contribution terms in the binding free energies of the inhibitors against the SQLE enzyme. The free energy order and thus the inhibition efficiency of the ligands against the enzyme indicate that 3b and 4b, followed by 9a and 1a, are the most potent inhibitors. According to the energy terms, the vdW energy for 3b and 4b is highest among the others, which can be attributed to the formation of salt bridges incorporating the positive charge of the ‐NH2 group in Arg43 and two negative groups of the carboxyl group in ligands. In addition, the *π*–stacking interaction between rings in these ligands with Pro41 and Tyr203 makes vdW energy more favorable for these ligands.

The MMPBSA formulation can provide supplementary information as a complementary examination to evaluate the comparative contribution of every residue to the binding free energy of the enzyme/ligand complex. The outcomes are depicted in Figure 6. Another piece of evidence for the crucial residues in the active site of the enzyme, which have a tremendous impact on the accommodation of ligands, can be found in such a plot. In this figure, the total binding free energy for each residue is depicted by the blue bars, representing the sum of vdW, electrostatic (Ele), polar solvation (Polar), and nonpolar solvation (NonPolar) energies.

Figure 6Ligand–residue energy decomposition for Compounds (a) 1a, (b) 2a, (c) 1b, (d) 3a, (e) 3b, (f) 5b, (g) 9a, and (h) 10a.

**Figure:**
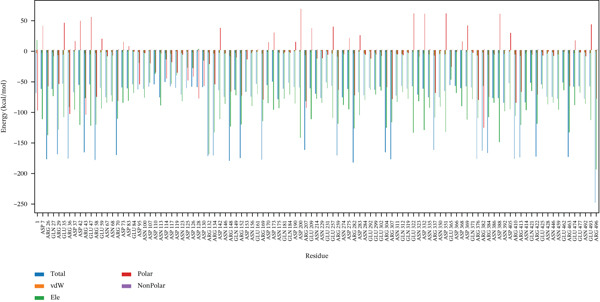
(a)

**Figure:**
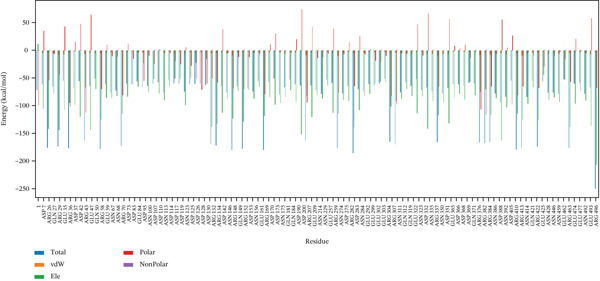
(b)

**Figure:**
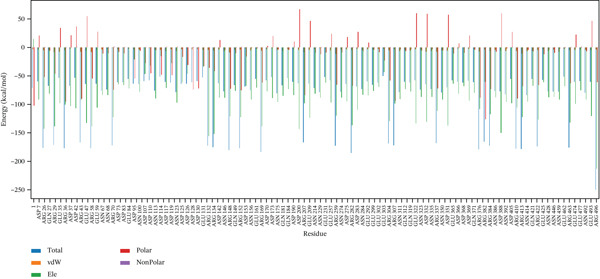
(c)

**Figure:**
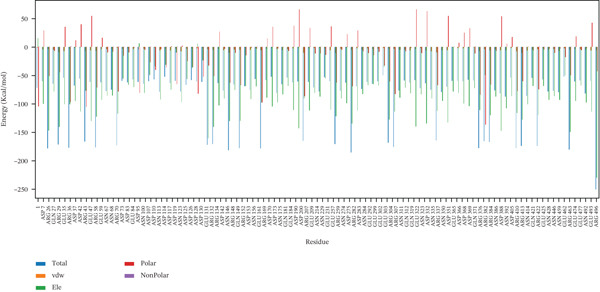
(d)

**Figure:**
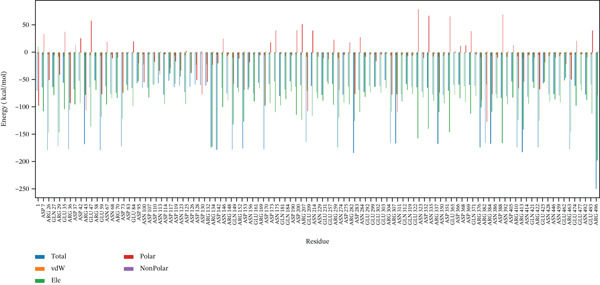
(e)

**Figure:**
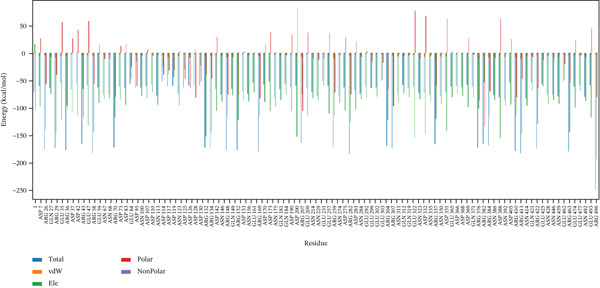
(f)

**Figure:**
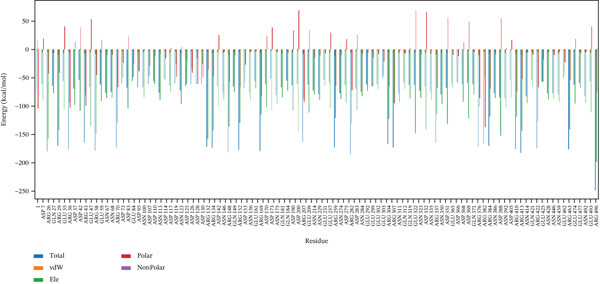
(g)

**Figure:**
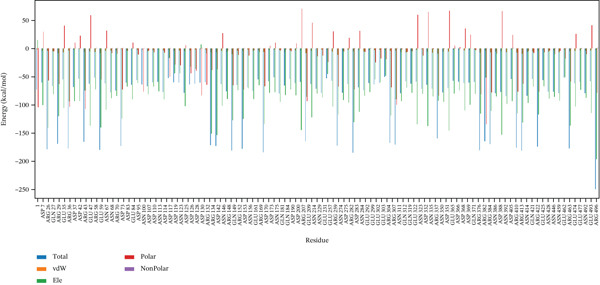
(h)

According to Figure 6, we can see the highest contribution of arginine residues in the binding energy of ligands bound to the enzyme. Among these arginine residues, we can notice the role of Arg43, which is part of the active site of the enzyme. According to Figure 6a, the energy decomposition analysis reveals that both hydrophobic (vdW) and Ele interactions play significant roles in the binding of Compound 1a to the protein. The residues Gly53, Tyr77, Leu48, Glu47, Val45, and Pro339 are critical for the binding stability, each contributing differently through various types of interactions. The detailed interaction map from the clustering analysis provides insight into how these residues interact with the ligand, confirming the energy decomposition findings. Figure 6b illustrates the energy decomposition analysis for Compound 2a. Similar to Compound 1a, certain residues significantly contribute to the binding energy of Compound 2a, stabilizing or destabilizing the complex.

## 4. Discussion

Over the past several decades, significant efforts have been made to develop novel antifungal agents in response to the limitations of existing therapies, including toxicity, resistance, and drug–drug interactions [47–50]. Despite these efforts, only a limited number of antifungal drugs have been successfully introduced into clinical use, underscoring the need for new compounds with distinct mechanisms of action.

Ergosterol biosynthesis represents a critical and well‐established target for antifungal drug development. Within this pathway, SQLE plays a pivotal role as the first oxygen‐dependent enzyme, catalyzing the conversion of squalene to 2,3‐oxidosqualene. Inhibition of SQLE disrupts ergosterol production, resulting in squalene accumulation and subsequent fungal cell death. Terbinafine, an allylamine antifungal agent, exerts its antifungal activity primarily through this mechanism. In the present study, novel antifungal compounds were designed based on the structural features of terbinafine, with a particular focus on enyne functional groups to enhance interactions within the SQLE active site. The biological evaluation demonstrated that the synthesized compounds exhibited antifungal activity, supporting their potential role as SQLE inhibitors.

Computational analyses provided further insight into the molecular basis of ligand binding. Energy decomposition analysis revealed that vdW and nonpolar interactions are the dominant contributors to ligand stabilization within the enzyme active site. These interactions are primarily associated with hydrophobic contacts between the alkyl chains of the ligands and the surrounding residues of SQLE.

Among the active site residues, Phe820 was identified as a key contributor to ligand binding, exerting a significant stabilizing effect through vdW interactions, particularly in the case of CAF and the synthesized ligands. In addition, metal–ligand interactions were found to influence binding affinity. The presence of zinc ions contributed to long‐range Ele interactions, which enhanced ligand stabilization. In contrast, Polar energy opposed ligand binding, indicating that excessive polarity may reduce binding efficiency.

Residues located around Ala767, including Asp764, Leu764, Leu765, and Ile768, showed minimal contribution to the binding energy, suggesting a limited role in ligand stabilization. Similarly, residues such as Gln817, Met816, and Gly819 displayed weak dynamic contributions through vdW interactions, which were partially counteracted by unfavorable Polar effects.

Taken together, the biological and computational findings indicate that the synthesized compounds interact effectively with key regions of the SQLE active site, primarily through hydrophobic interactions. These results support the potential of the designed scaffold as a promising lead for the development of new antifungal agents targeting ergosterol biosynthesis.

## 5. Conclusion

Among the compounds, more than 10 compounds with the best docking results were selected for synthesis and then synthesized. Then, after synthesizing the compounds and confirming the structure by ^1^H‐NMR and ^13^C‐NMR spectroscopy and then checking the MIC on *C. albicans*, *S. cerevisiae*, and *A. fumigatus*, Compounds 1a, 2a, 3a, 4a, 9a, 10a, and2b showed good antifungal activity with 0.7–31.25 *μ*g/mL MIC. According to the in silico results and biological test results in this research, we are able to predict that the compound with a triple bond (acetylene) can also have antifungal activity. With this reasonable possibility, in addition, the effect of triple band position was compared to other functional groups. In previous research, according to the results, it was determined that capillin is a compound that has a diyne bond and is one of the antifungal agents that has two conjugated triple bonds. On the other hand, according to our results and previous research, it was found that the addition of a carbonyl group to the conjugation position of the triple bond significantly increases the antifungal property. This effect of increased activity can be seen in Group A compounds (esters). However, according to the bioinformatic results as well as the antifungal results for the compounds with acetylene functional group and carbonyl group in the conjugated position of the triple bond (compounds of the first stage named a1–a10) and also the placement of two enyne groups in a row (compounds of the second stage named 1b–10b), it was determined that the MIC activity of Compounds 1a, 2a, 9a, 10a, and 2b was good. One of the most important reasons for this decrease in activity effect can be due to the special arrangement of functional groups, in which the addition of another carbonyl group with electron‐absorbing properties is next to the double bond. In addition, considering that the MIC of Compounds 1a, 2a, 9a, and 10a is very close to the patterns of the main molecule (terbinafine), the lack of activity or the weakening of antifungal activity in Compounds 1b, 9b, and 10b can be attributed to chain lengthening due to reaction. Coupling and the position of the rings that affect the shape and lipophilicity were attributed. These data show that in enyne compounds, both the chain length and the presence of aromatic rings and the position of triple and double bonds have an effect on the activity and minimum concentration of the inhibitor. In the continuation of the research, in order to investigate more precisely the mechanism of the effect of synthetic compounds on *C. albicans*, three combinations of compounds that had the best MIC results (2a, 10a, and 2b) were selected, and SEM and TEM images were selected (TEM is outside the plan, and more methods are mentioned). Compared to the control, it was found that the morphology of the cells was messed up and underwent changes, including shape change and, in some cases, perforation of the *Candida* cell wall. Next, the amount of ergosterol was also measured. Curves represent *Candida* sterols that were grown for 24 h in SDB and analyzed by UV spectrophotometers. The graphs show the absorbance of 2 MIC, MIC, and 1/2 MIC at the wavelength of 200–500 nm, from top to bottom. As it is known, ergosterol peaks of three curves between the wavelength (−240 to 300 nm) are reduced, and as a result, squalene, the precursor of ergosterol, shows peaks at 220–200 nm. Therefore, according to these findings, new molecules with different structural pharmacophores were designed based on the method derived from the structural model of terbinafine, as well as molecular docking, resulting in new compounds that had good antifungal properties.

## Author Contributions

Mohadese Mohammadi performed the synthesis procedures and molecular docking analyses and prepared the initial draft of the manuscript. Parviz Rashidi Ranjbar contributed to the conceptualization of the study and provided critical supervision throughout the project. Parisa Azerang designed and conducted the biological assays, including SEM analysis, was responsible for manuscript development and project supervision, and prepared the final draft of the manuscript. Soroush Sardari contributed to project conceptualization, supervised the research activities, and supported the bioinformatics analysis and data interpretation. Mohadese Mohammadi and Parviz Rashidi Ranjbar contributed equally.

## Funding

This study was funded by the Pasteur Institute of Iran, 10.13039/501100010679, 1169.

## Disclosure

All authors reviewed and approved the final version of the manuscript.

## Conflicts of Interest

The authors declare no conflicts of interest.

## Supporting information


**Supporting Information Figure S1:** Additional supporting information can be found online in the Supporting Information section. Figure S1: Full ^1^H‐NMR and ^13^C‐NMR and mass spectra for all synthesized compounds (1a–10a and 1b–10b). Table S1: Full MIC datasets. Table S2: Full docking tables.

## Data Availability

The data that support the findings of this study are available on request from the corresponding authors. The data are not publicly available due to privacy or ethical restrictions.

## References

[1] Enoch D. , Ludlam H. , and Brown N. , Invasive Fungal Infections: A Review of Epidemiology and Management Options, Journal of Medical Microbiology. (2006) 55, no. 7, 809–818, 10.1099/jmm.0.46548-0. 16772406

[2] Suleyman G. and Alangaden G. J. , Nosocomial Fungal Infections: Epidemiology, Infection Control, and Prevention, Infectious Disease Clinics of North America. (2016) 30, no. 4, 1023–1052, 10.1016/j.idc.2016.07.008. 27816138

[3] Sardi J. C. O. , Scorzoni L. , Bernardi T. , Fusco-Almeida A. M. , and Mendes Giannini M. J. S. , *Candida* Species: Current Epidemiology, Pathogenicity, Biofilm Formation, Natural Antifungal Products and New Therapeutic Options, Journal of Medical Microbiology. (2013) 62, no. 1, 10–24, 10.1099/jmm.0.045054-0. 23180477

[4] Rajasekar V. , Darne P. , Prabhune A. , Kao R. Y. , Solomon A. P. , Ramage G. , Samaranayake L. P. , and Neelakantan P. , A Curcumin-Sophorolipid Nanocomplex Inhibits *Candida albicans* Filamentation and Biofilm Development, Colloids and Surfaces B: Biointerfaces. (2021) 200, 111617, 10.1016/j.colsurfb.2021.111617, 33592455. 33592455

[5] Enoch D. A. , Yang H. , Aliyu S. H. , and Micallef C. , The Changing Epidemiology of Invasive Fungal Infections, Human Fungal Pathogen Identification: Methods and Protocols, 2017, Springer, 17–65, 10.1007/978-1-4939-6515-1_2. 27837497

[6] Santiago T. M. G. , Pritt B. , Gibson L. E. , and Comfere N. I. , Diagnosis of Deep Cutaneous Fungal Infections: Correlation Between Skin Tissue Culture and Histopathology, Journal of the American Academy of Dermatology. (2014) 71, no. 2, 293–301, 10.1016/j.jaad.2014.03.042, 24836547. 24836547

[7] Ngo H. X. , Garneau-Tsodikova S. , and Green K. D. , A Complex Game of Hide and Seek: The Search for New Antifungals, Medicinal Chemistry Communications. (2016) 7, no. 7, 1285–1306, 10.1039/C6MD00222F. 27766140 PMC5067021

[8] Sheng C. and Zhang W. , New Lead Structures in Antifungal Drug Discovery, Current Medicinal Chemistry. (2011) 18, no. 5, 733–766, 10.2174/092986711794480113, 21182484. 21182484

[9] Fang J. , Huang B. , and Ding Z. , Efficacy of Antifungal Drugs in the Treatment of Oral Candidiasis: A Bayesian Network Meta-Analysis, The Journal of Prosthetic Dentistry. (2021) 125, no. 2, 257–265, 10.1016/j.prosdent.2019.12.025. 32165010

[10] Mehrandish S. and Mirzaeei S. , A Review on Ocular Novel Drug Delivery Systems of Antifungal Drugs: Functional Evaluation and Comparison of Conventional and Novel Dosage Forms, Advanced Pharmaceutical Bulletin. (2021) 11, no. 1, 28–38, 10.34172/apb.2021.003, 33747850. 33747850 PMC7961232

[11] Godge G. R. , Bharat S. C. , Shaikh A. B. , Randhawan B. B. , Raskar M. A. , and Hiremath S. N. , Formulation Perspectives in Topical Antifungal Drug Therapy: A Review, Journal of Drug Delivery and Therapeutics. (2023) 13, no. 5, 110–119, 10.22270/jddt.v13i5.6079.

[12] Day A. W. , Hayes E. , Perez-Lozada J. , DiLeo A. , Blandino K. , Maguire J. , and Kumamoto C. A. , Candida Albicanscolonization Modulates Murine Ethanol Consumption and Behavioral Responses Through Elevation of Serum Prostaglandin E2and Impact on the Striatal Dopamine System, MBio. (2025) 16, no. 11, e02239-25, 10.1128/mbio.02239-25, 41099524. 41099524 PMC12607880

[13] Romo J. A. , Pierce C. G. , Chaturvedi A. K. , Lazzell A. L. , SF M. H. , Saville S. P. , and Lopez-Ribot J. L. , Development of Anti-Virulence Approaches for Candidiasis via a Novel Series of Small-Molecule Inhibitors of Candida albicans Filamentation, MBio. (2017) 8, no. 6, 10–1128, 10.1128/mbio.01991-17. PMC5717394 29208749

[14] Park S. , Kelly R. , Kahn J. N. , Robles J. , Hsu M.-J. , Register E. , Li W. , Vyas V. , Fan H. , Abruzzo G. , Flattery A. , Gill C. , Chrebet G. , Parent S. A. , Kurtz M. , Teppler H. , Douglas C. M. , and Perlin D. S. , Specific Substitutions in the Echinocandin Target Fks1p Account for Reduced Susceptibility of Rare Laboratory and Clinical *Candida* sp. Isolates, Antimicrobial Agents and Chemotherapy. (2005) 49, no. 8, 3264–3273, 10.1128/AAC.49.8.3264-3273.2005, 16048935. 16048935 PMC1196231

[15] Harvey A. , Strategies for Discovering Drugs From Previously Unexplored Natural Products, Drug Discovery Today. (2000) 5, no. 7, 294–300, 10.1016/S1359-6446(00)01511-7, 10856912. 10856912

[16] Hui S. T. , Gifford H. , and Rhodes J. , Emerging Antifungal Resistance in Fungal Pathogens, Current Clinical Microbiology Reports. (2024) 11, no. 2, 43–50, 10.1007/s40588-024-00219-8, 38725545. 38725545 PMC11076205

[17] Cao X. , Xu Y. , Cao Y. , Wang R. , Zhou R. , Chu W. , and Yang Y. , Design, Synthesis, and Structure–Activity Relationship Studies of Novel Thienopyrrolidone Derivatives With Strong Antifungal Activity Against Aspergillus fumigates, European Journal of Medicinal Chemistry. (2015) 102, 471–476, 10.1016/j.ejmech.2015.08.023. 26310892

[18] Nowosielski M. , Hoffmann M. , Wyrwicz L. S. , Stepniak P. , Plewczynski D. M. , Lazniewski M. , Ginalski K. , and Rychlewski L. , Detailed Mechanism of Squalene Epoxidase Inhibition by Terbinafine, Journal of Chemical Information and Modeling. (2011) 51, no. 2, 455–462, 10.1021/ci100403b. 21229992

[19] Vogelsinger H. , Weiler S. , Djanani A. , Kountchev J. , Bellmann-Weiler R. , Wiedermann C. J. , and Bellmann R. , Amphotericin B tissue distribution in autopsy material after treatment with liposomal amphotericin B and amphotericin B colloidal dispersion, Journal of Antimicrobial Chemotherapy. (2006) 57, no. 6, 1153–1160, 16627591. 16627591 10.1093/jac/dkl141

[20] Alvarez F. J. , Douglas L. M. , and Konopka J. B. , Sterol-Rich Plasma Membrane Domains in Fungi, Eukaryotic Cell. (2007) 6, no. 5, 755–763, 10.1128/EC.00008-07, 17369440. 17369440 PMC1899238

[21] Sah S. K. , Hayes J. J. , and Rustchenko E. , The Role of Aneuploidy in the Emergence of Echinocandin Resistance in Human Fungal Pathogen *Candida albicans* , PLoS Pathogens. (2021) 17, no. 5, e1009564, 10.1371/journal.ppat.1009564. 34043737 PMC8158998

[22] Yousfi H. , Ranque S. , Rolain J.-M. , and Bittar F. , In Vitro Polymyxin Activity Against Clinical Multidrug-Resistant Fungi, Antimicrobial Resistance and Infection Control. (2019) 8, no. 1, 10.1186/s13756-019-0521-7. PMC6480676 31044071

[23] Ranu B. C. and Chattopadhyay K. , A New Route to the Synthesis of (E)- and (Z)-2-Alkene-4-Ynoates and Nitriles From Vic-Iiodo-(E)-Alkenes Catalyzed by Pd(0) Nanoparticles in Water, Organic Letters. (2007) 9, no. 12, 2409–2412, 10.1021/ol0708121, 17488036. 17488036

[24] Tai H.-H. and Bloch K. , Squalene Epoxidase of Rat Liver, The Journal of Biological Chemistry. (1972) 247, no. 12, 3767–3773, 10.1016/S0021-9258(19)45101-6, 5033388. 5033388

[25] Pelletier G. , Lie S. , Mousseau J. J. , and Charette A. B. , One-Pot Synthesis of 1-Iodoalkynes and Trisubstituted Alkenes From Benzylic and Allylic Bromides, Organic Letters. (2012) 14, no. 21, 5464–5467, 10.1021/ol302544s. 23075119

[26] Borba-Santos L. P. , Rodrigues A. M. , Gagini T. B. , Fernandes G. F. , Castro R. , de Camargo Z. P. , Nucci M. , Lopes-Bezerra L. M. , Ishida K. , and Rozental S. , Susceptibility of *Sporothrix brasiliensis* Isolates to Amphotericin B, Azoles, and Terbinafine, Sabouraudia. (2015) 53, no. 2, 178–188, 10.1093/mmy/myu056. 25394542

[27] Azerang P. and Sardari S. , Antifungal Activity of Enynediesters and Acetylenic Compounds Obtained by Synthesis and in Silico Prediction Pattern, JOURNAL DE MYCOLOGIE MEDICALE.(2012) 22, no. 3, 230–236, 10.1016/j.mycmed.2012.06.001, 23518080. 23518080

[28] Ramachandran P. V. , Rudd M. T. , and Reddy M. V. R. , Stereoselective Synthesis of Hex-2-(*E*)-En-4-yn-1,6-Dioates and *E*,*Z*-Muconic Acid Diesters via Organo-Catalyzed Self-Coupling of Propiolates, Tetrahedron Letters. (2005) 46, no. 15, 2547–2549, 10.1016/j.tetlet.2005.02.098.

[29] Buil J. B. , Hagen F. , Chowdhary A. , Verweij P. E. , and Meis J. F. , Itraconazole, Voriconazole, and Posaconazole CLSI MIC Distributions for Wild-Type and Azole-Resistant *Aspergillus fumigatus* Isolates, Journal of Fungi. (2018) 4, no. 3, 10.3390/jof4030103. PMC6162657 30158470

[30] Iman M. , Davood A. , Gebbink B. K. , Azerang P. , Alibolandi M. , and Sardari S. , Design and Antimicrobial Evaluation of 1-Methylimidazole Derivatives as New Antifungal and Antibacterial Agents, Pharmaceutical Chemistry Journal. (2014) 48, 513–519, 10.1007/s11094-014-1140-5.

[31] Tyagi P. , Singh M. , Kumari H. , Kumari A. , and Mukhopadhyay K. , Bactericidal Activity of Curcumin I Is Associated With Damaging of Bacterial Membrane, PLoS One. (2015) 10, no. 3, e0121313, 10.1371/journal.pone.0121313. 25811596 PMC4374920

[32] Kerfeld C. A. and Scott K. M. , Using BLAST to Teach “E-Value-Tionary” Concepts, PLoS Biology. (2011) 9, no. 2, e1001014, 10.1371/journal.pbio.1001014. 21304918 PMC3032543

[33] Zhang Y. , Interplay of I‐TASSER and QUARK for Template‐Based and Ab Initio Protein Structure Prediction in CASP10, Proteins. (2014) 82, no. S2, 175–187, 10.1002/prot.24341. PMC4067246 23760925

[34] Padalkar V. S. , Borse B. N. , Gupta V. D. , Phatangare K. R. , Patil V. S. , and Sekar N. , Synthesis and Antimicrobial Activities of Novel 2-[Substituted-1*H*-Pyrazol-4-Yl] Benzothiazoles, Benzoxazoles, and Benzimidazoles, Journal of Heterocyclic Chemistry. (2016) 53, no. 5, 1347–1355, 10.1002/jhet.1506.

[35] Patil V. S. , Padalkar V. S. , Phatangare K. R. , Umape P. G. , Borase B. N. , and Sekar N. , Synthesis, Characterization, and Antibacterial Activity of Novel (1H‐Benzo[d]imidazole‐2‐yl)‐6‐(diethylamino)‐3H‐one‐xanthene, Phenoxazine, and Oxazine, Journal of Heterocyclic Chemistry. (2015) 52, no. 1, 124–129, 10.1002/jhet.1998.

[36] Sun H. , Duan L. , Chen F. , Liu H. , Wang Z. , Pan P. , and Hou T. , Assessing the Performance of MM/PBSA and MM/GBSA Methods. 7. Entropy Effects on the Performance of End-Point Binding Free Energy Calculation Approaches, Physical Chemistry Chemical Physics. (2018) 20, no. 21, 14450–14460, 10.1039/c7cp07623a, 29785435. 29785435

[37] Kiani Y. S. , Ranaghan K. E. , Jabeen I. , and Mulholland A. J. , Molecular Dynamics Simulation Framework to Probe the Binding Hypothesis of CYP3A4 Inhibitors, International Journal of Molecular Sciences. (2019) 20, no. 18, 10.3390/ijms20184468. PMC6769491 31510073

[38] Roe D. R. and Cheatham T. E. I. I. I. , PTRAJ and CPPTRAJ: Software for Processing and Analysis of Molecular Dynamics Trajectory Data, Journal of Chemical Theory and Computation. (2013) 9, no. 7, 3084–3095, 10.1021/ct400341p, 26583988. 26583988

[39] Card G. L. , England B. P. , Suzuki Y. , Fong D. , Powell B. , Lee B. , Luu C. , Tabrizizad M. , Gillette S. , Ibrahim P. N. , Artis D. R. , Bollag G. , Milburn M. V. , Kim S. H. , Schlessinger J. , and Zhang K. Y. J. , Structural Basis for the Activity of Drugs That Inhibit Phosphodiesterases, Structure. (2004) 12, no. 12, 2233–2247, 10.1016/j.str.2004.10.004. 15576036

[40] Phatangare K. R. , Borse B. N. , Padalkar V. S. , Patil V. S. , Gupta V. D. , Umape P. G. , and Sekar N. , Synthesis, Photophysical Property Study of Novel Fluorescent 4-(1, 3-Benzoxazol-2-Yl)-2-Phenylnaphtho [1, 2-*d*][1, 3] Oxazole Derivatives and Their Antimicrobial Activity, Journal of Chemical Sciences. (2013) 125, 141–151, 10.1007/s12039-012-0324-3.

[41] Padalkar V. S. , Gupta V. D. , Phatangare K. R. , Patil V. S. , Umape P. G. , and Sekar N. , Synthesis of Novel Dipodal-Benzimidazole, Benzoxazole and Benzothiazole From Cyanuric Chloride: Structural, Photophysical and Antimicrobial Studies, Journal of Saudi Chemical Society. (2014) 18, no. 3, 262–268, 10.1016/j.jscs.2011.07.001.

[42] Kashani E. , Pesyan N. , Rashidnejad H. , Poursattar A. , and Yaghoobnejad A. H. , Synthesis and Characterization of Novel Polymeric Organic–Inorganic Complex Framework Based on Sodium 2, 4-Dioxo-6-Aryl-3-Oxa-Bicyclo [3.1.0] Hexane-1, 5-Dicarboxylate (SDAOBDC) With Three-Dimensional Hybrid Networks, Journal of the Iranian Chemical Society. (2017) 14, 1–10, 10.1007/s13738-017-1151-8.

[43] Patil A. , Chaudhari V. , Patil S. R. , Borse G. P. , and Patil V. , A Bio-Waste Derived Sustainable Heterogenous Catalyst for Biginelli Reaction, Journal of the Indian Chemical Society. (2023) 100, no. 9, 101080, 10.1016/j.jics.2023.101080.

[44] Clinical and Laboratory Standards Institute (CLSI) , Performance Standards for Antifungal Susceptibility Testing of Filamentous Fungi; Approved Standard—M38M51S, 2022, CLSI.

[45] Arthington-Skaggs B. A. , Jradi H. , Desai T. , and Morrison C. J. , Quantitation of Ergosterol Content: Novel Method for Determination of Fluconazole Susceptibility of *Candida albicans* , Journal of Clinical Microbiology. (1999) 37, no. 10, 3332–3337, 10488201. 10488201 10.1128/jcm.37.10.3332-3337.1999 PMC85559

[46] Staniszewska S. , Kuznetsova M. M. , and de Groot J. J. , *Candida albicans* Morphologies Revealed by Scanning Electron Microscopy Analysis, Brazilian Journal of Microbiology. (2013) 44, no. 3, 813–821, 10.1590/S1517-83822013005000056, 24516422. 24516422 PMC3910194

[47] Varani T. , Abdouss M. , Azerang P. , and Tahghighi A. , Acetylenic Sulfones and Acetylenic Sulfonamide Analogs: A Novel and Preferable Antimicrobial Drugs Based on Computational Strategies, Journal of Computational Biophysics and Chemistry. (2022) 21, no. 1, 115–122, 10.1142/S2737416521410027.

[48] Nigam P. K. , Antifungal Drugs and *Resistance: Current Concepts* , Our Dermatology Online. (2015) 6, no. 2, 212–221, 10.7241/ourd.20152.58.

[49] Azerang P. , Khalaj V. , Kobarfard F. , Owlia P. , Sardari S. , and Shahidi S. , Molecular Characterization of A Fungus Producing Membrane Active Metabolite and Analysis of the Produced Secondary Metabolite, Iranian Biomedical Journal. (2019) 23, no. 2, 121–128, 10.29252/ibj.23.2.121, 30218995. 30218995 PMC6707112

[50] Zare S. , Emami L. , Faghih Z. , Zargari F. , Faghih Z. , and Khabnadideh S. , Design, Synthesis, Computational Study and Cytotoxic Evaluation of Some New Quinazoline Derivatives Containing Pyrimidine Moiety, Scientific Reports. (2023) 13, no. 1, 10.1038/s41598-023-41530-6, 37660139. PMC10475017 37660139

