# Supplementary material for: Design and Synthesis of 2‐En‐4‐Ynedioate Compounds as Novel and Potential Antifungal Agents
Source: Biomed Res Int. 2026 Jun 23;2026:5560073. doi: 10.1155/bmri/5560073 (PMC13291183; doi:10.1155/bmri/5560073)
Supplement: Supplementary file 1 — Supporting Information Figure S1: Additional supporting information can be found online in the Supporting Information section. Figure S1: Full 1H‐NMR and 13C‐NMR and mass spectra for all synthesized compounds (1a–10a and 1b–10b). Table S1: Full MIC datasets. Table S2: Full docking tables. [file BMRI-2026-5560073-s001.docx]

**Supporting Information**

**1- ^1^H, ^13^C1 NMR and Mass spectra for all synthesized compounds (1a–10a, 1b–10b).**

Propiolic acid


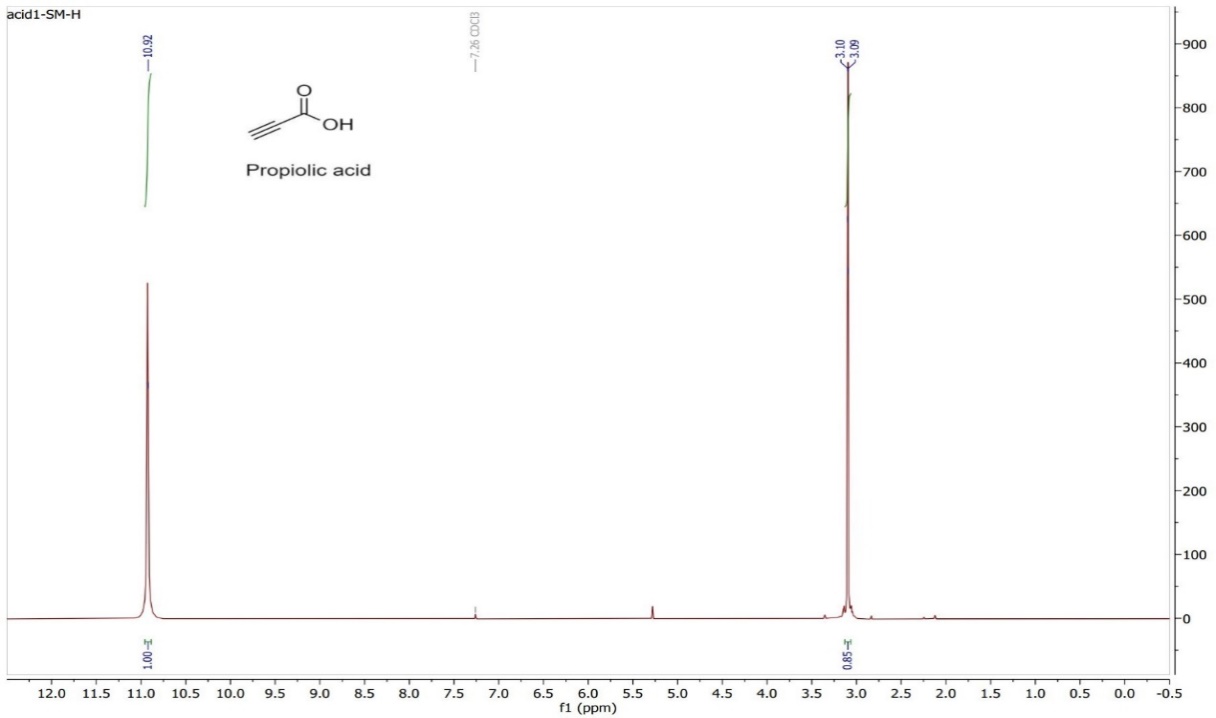


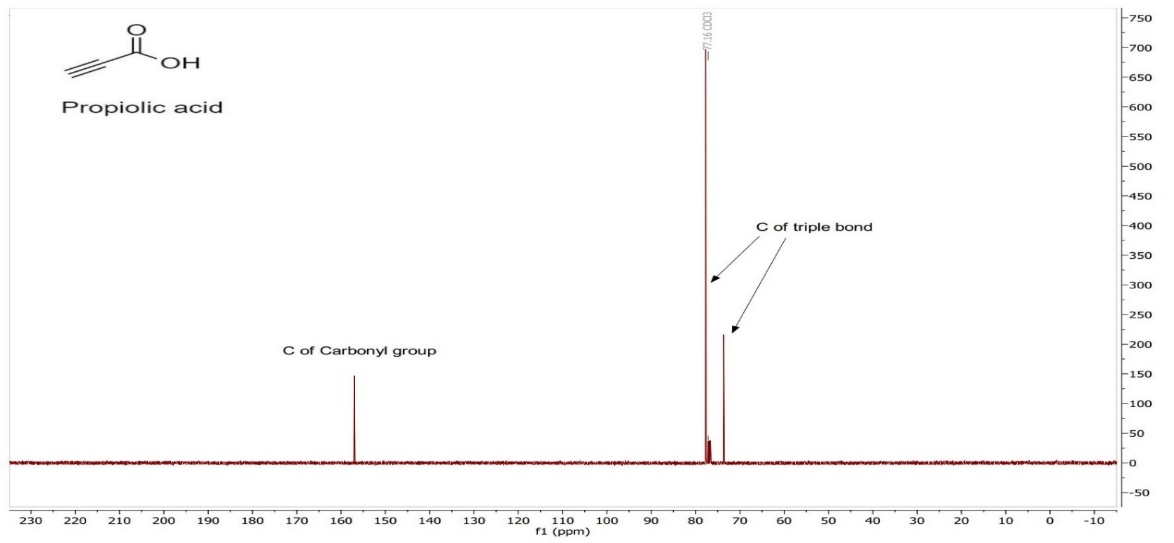


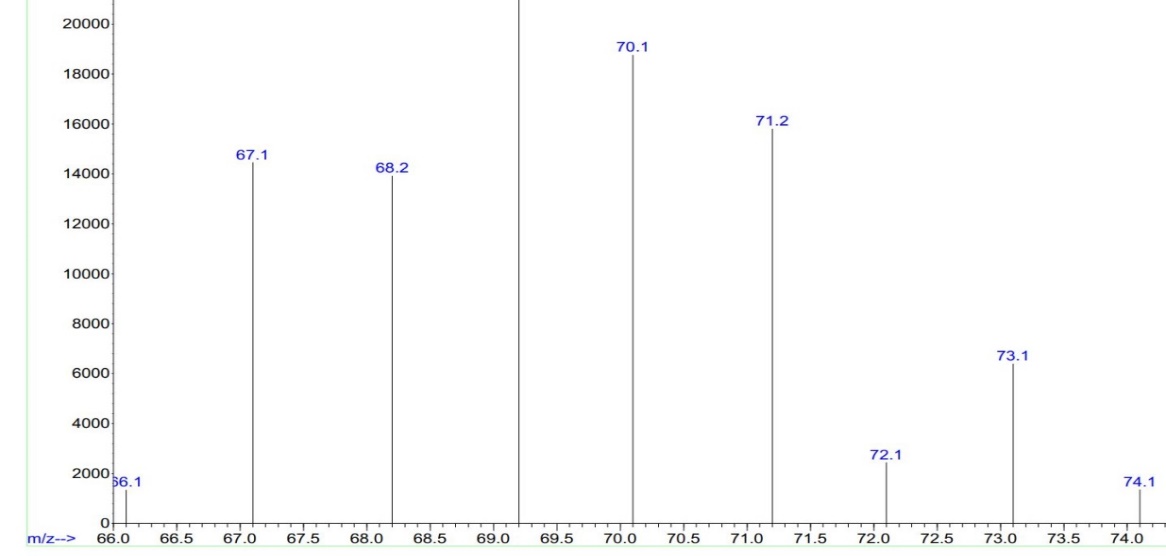


**1a:**


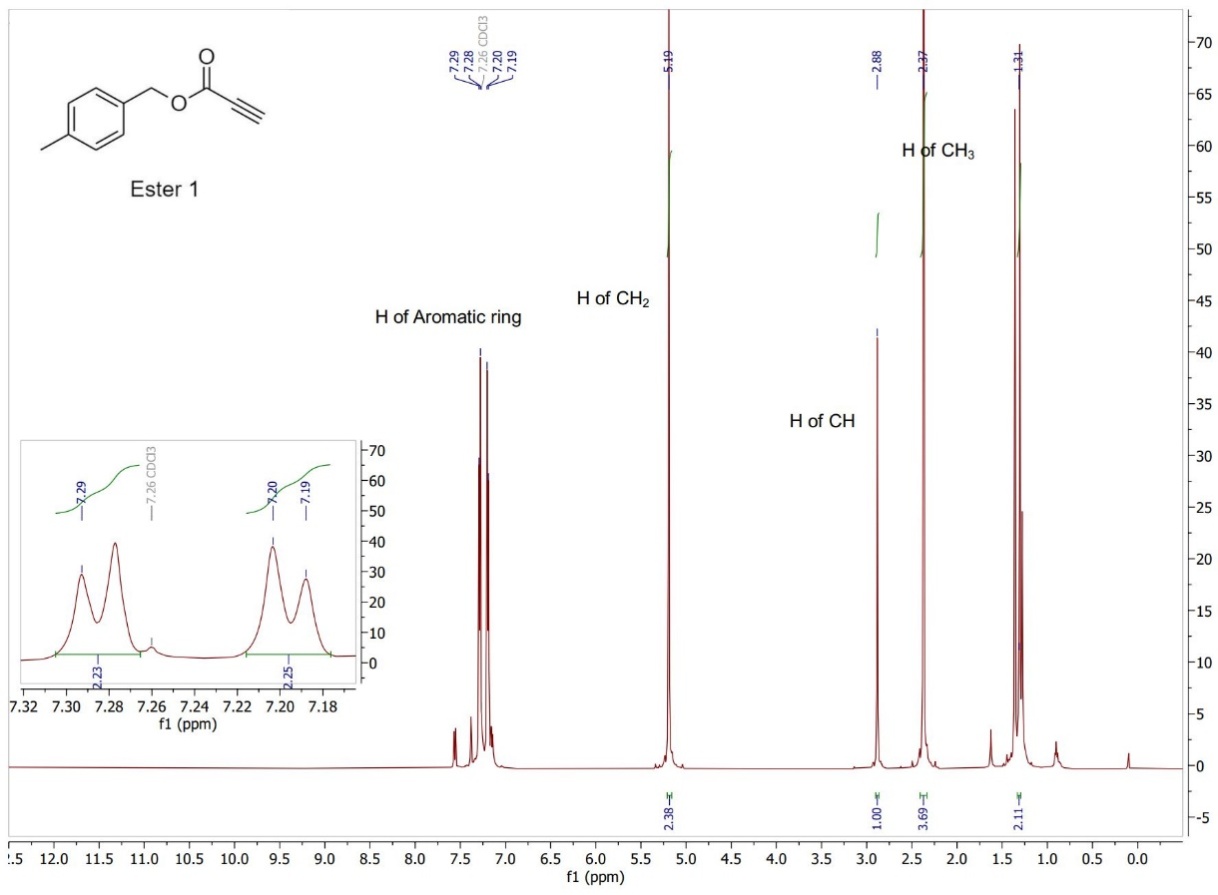


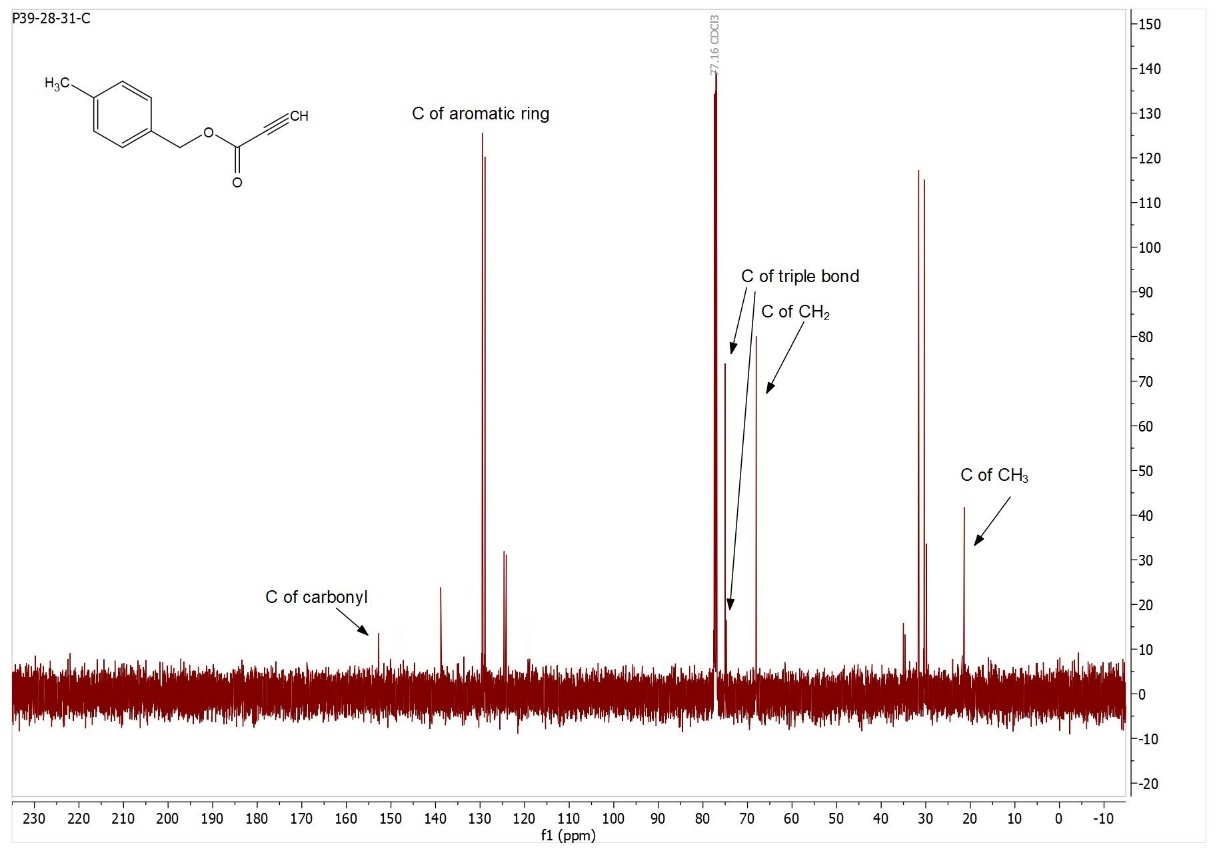


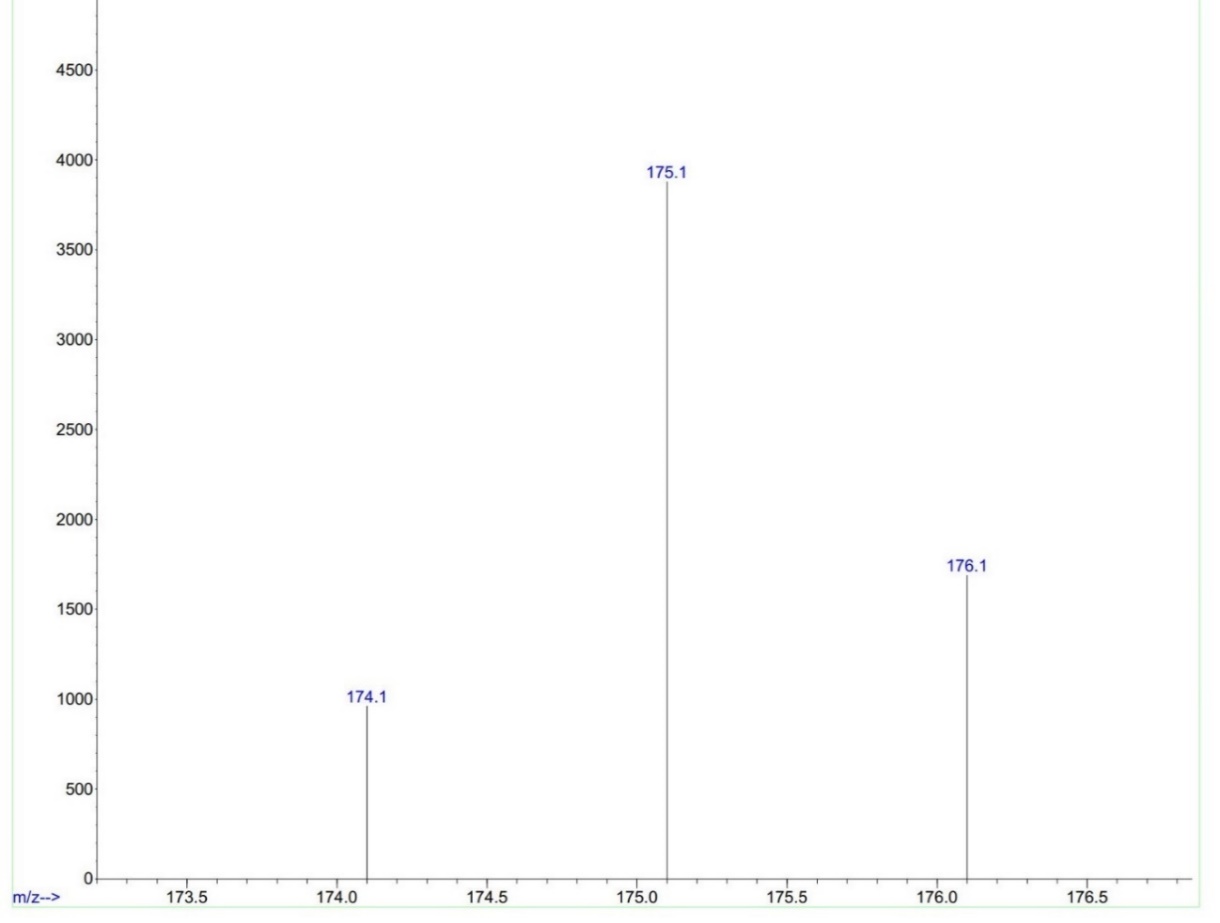


**2a:**


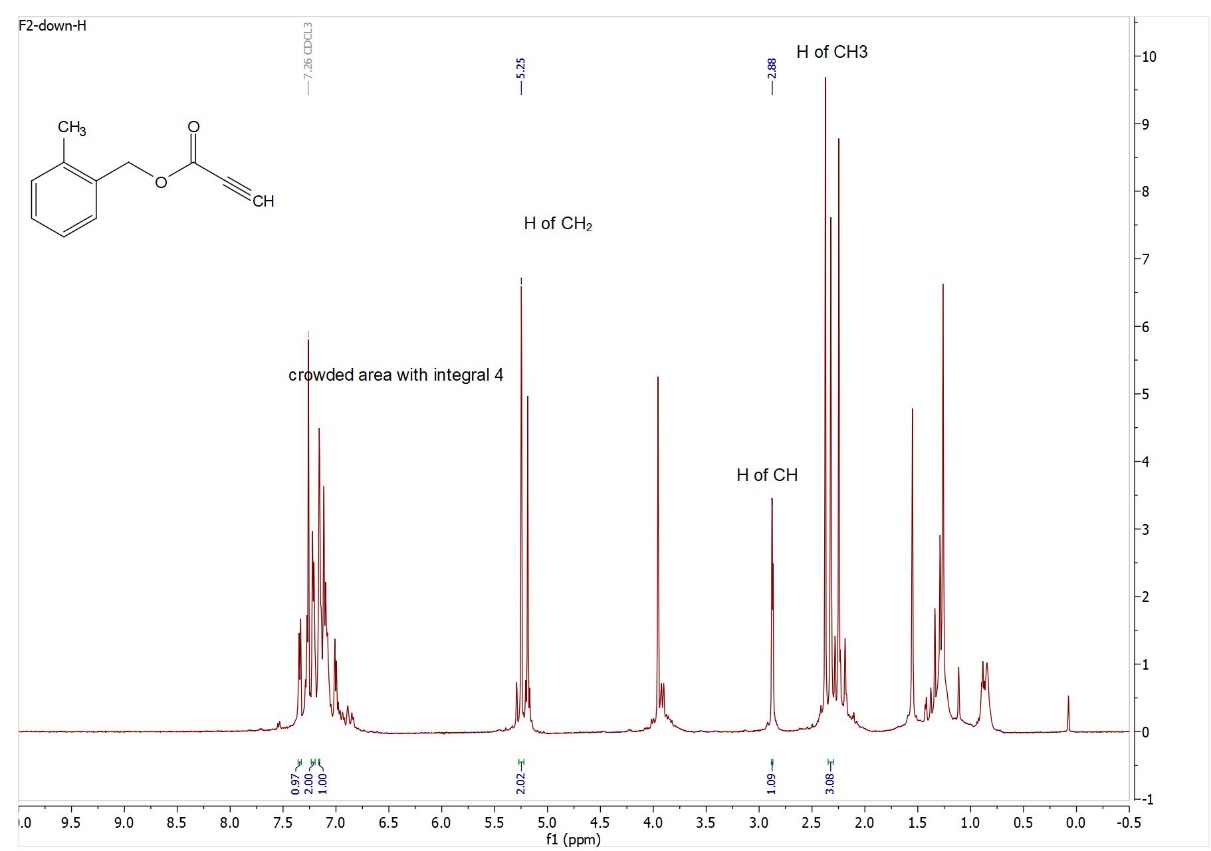


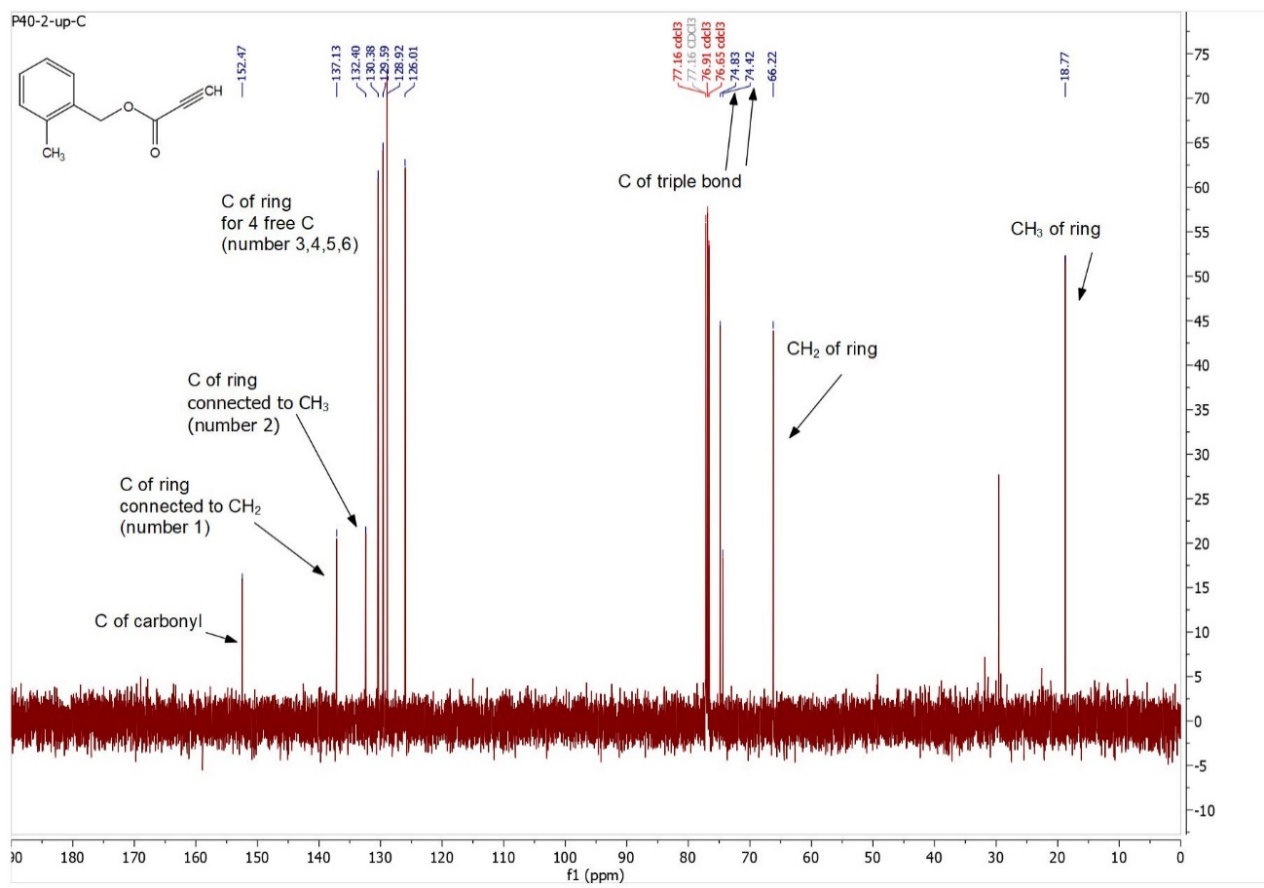


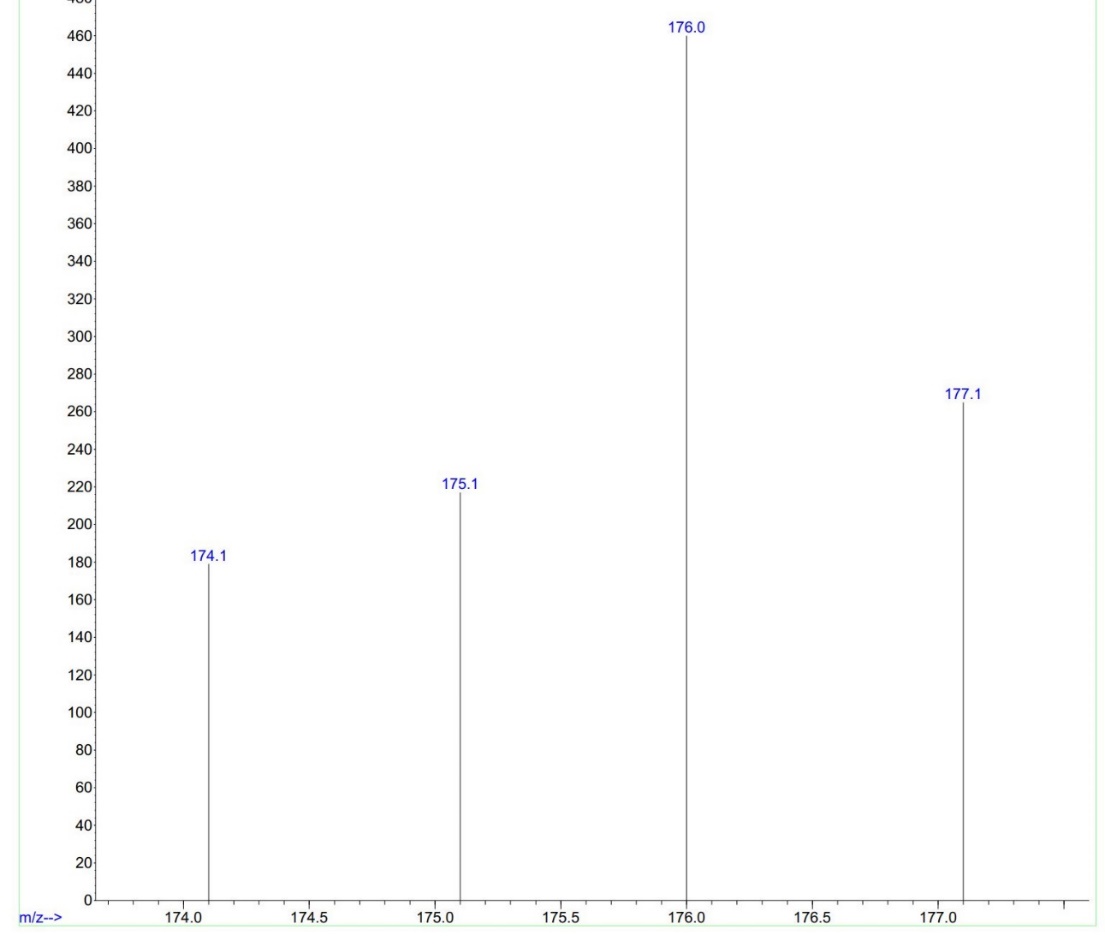


**3a:**


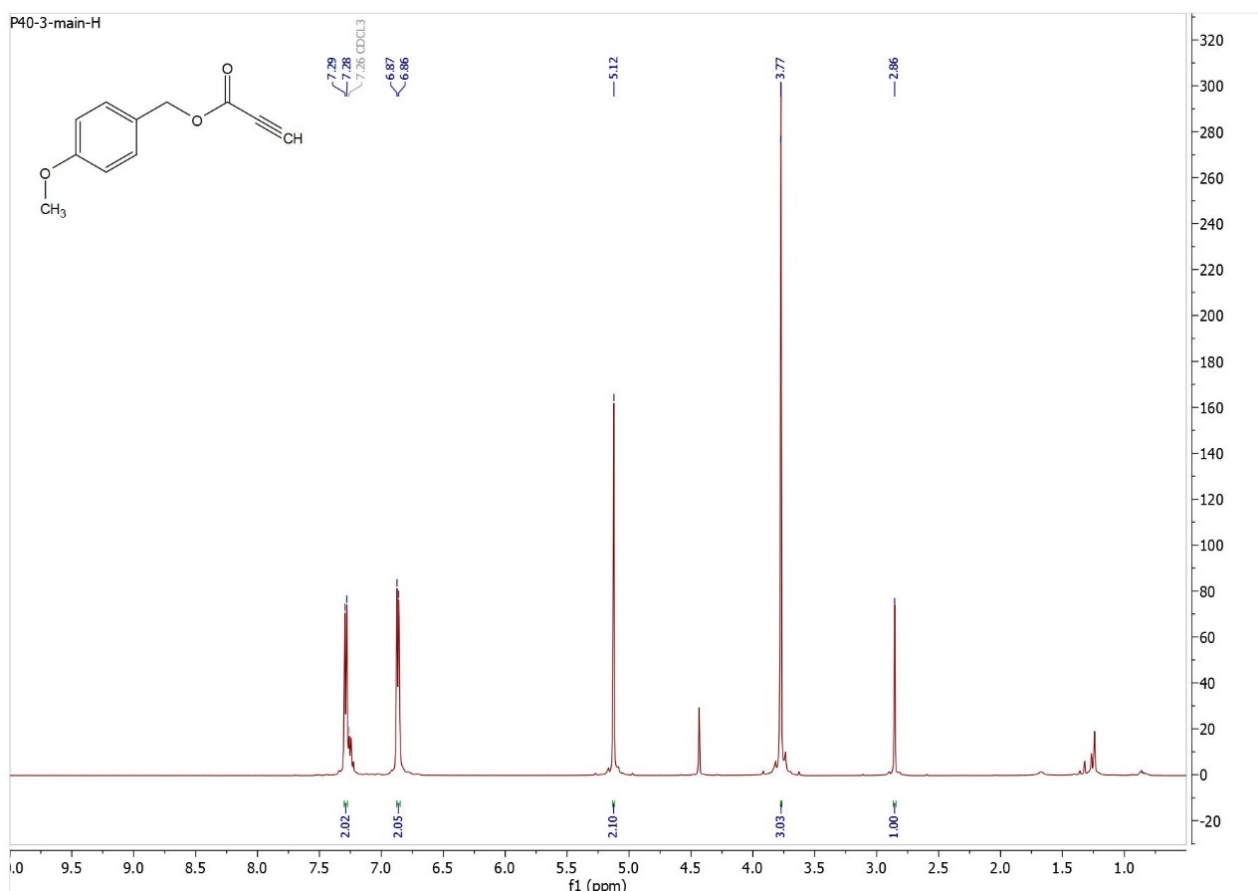


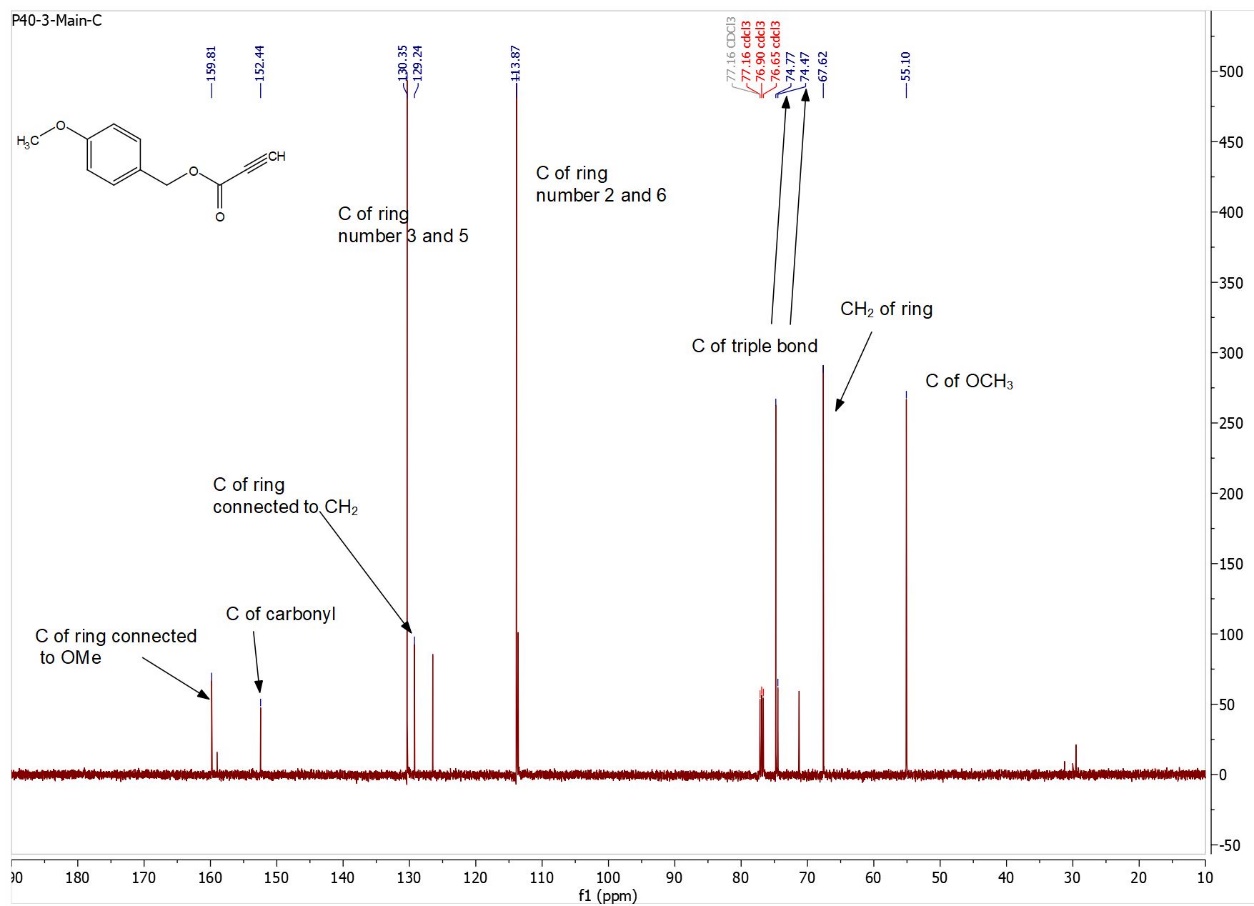


**4a:**


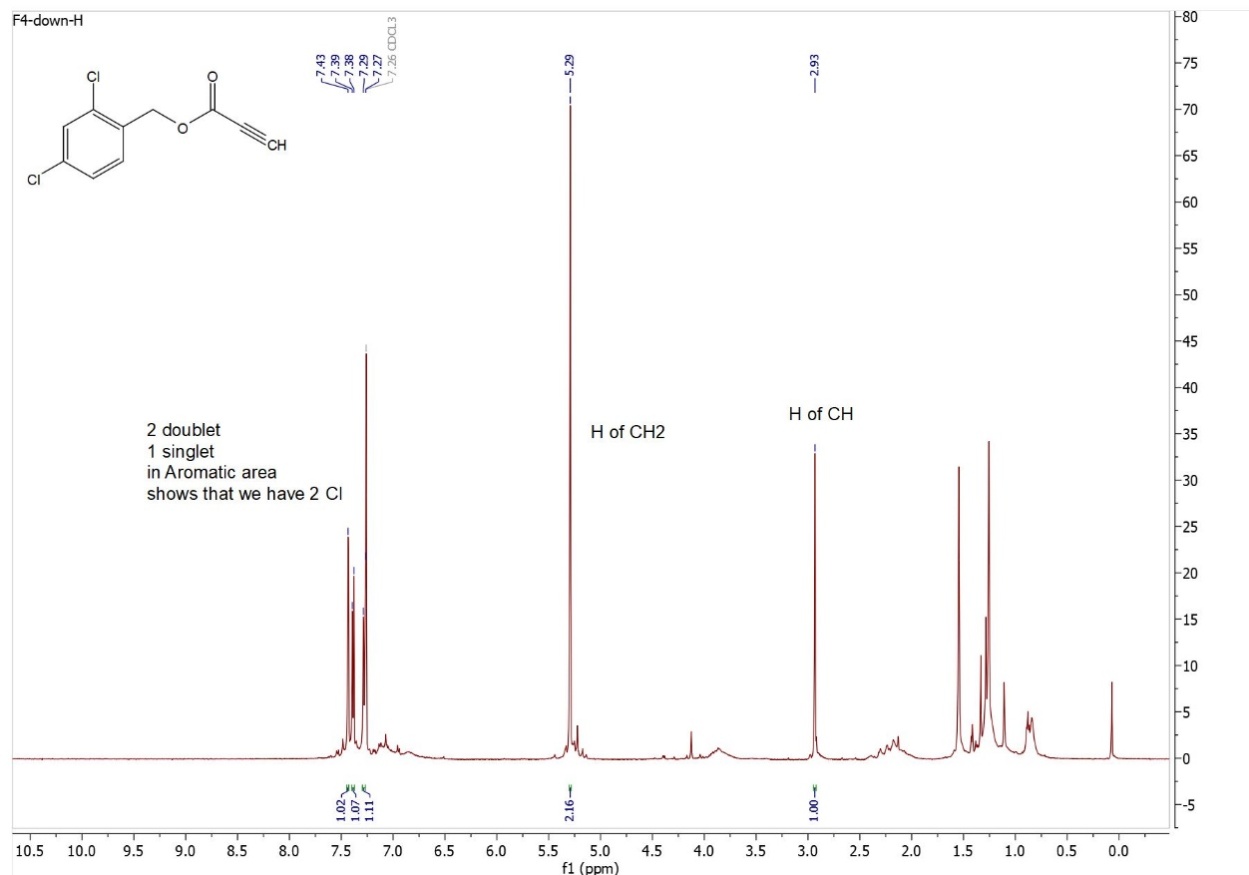


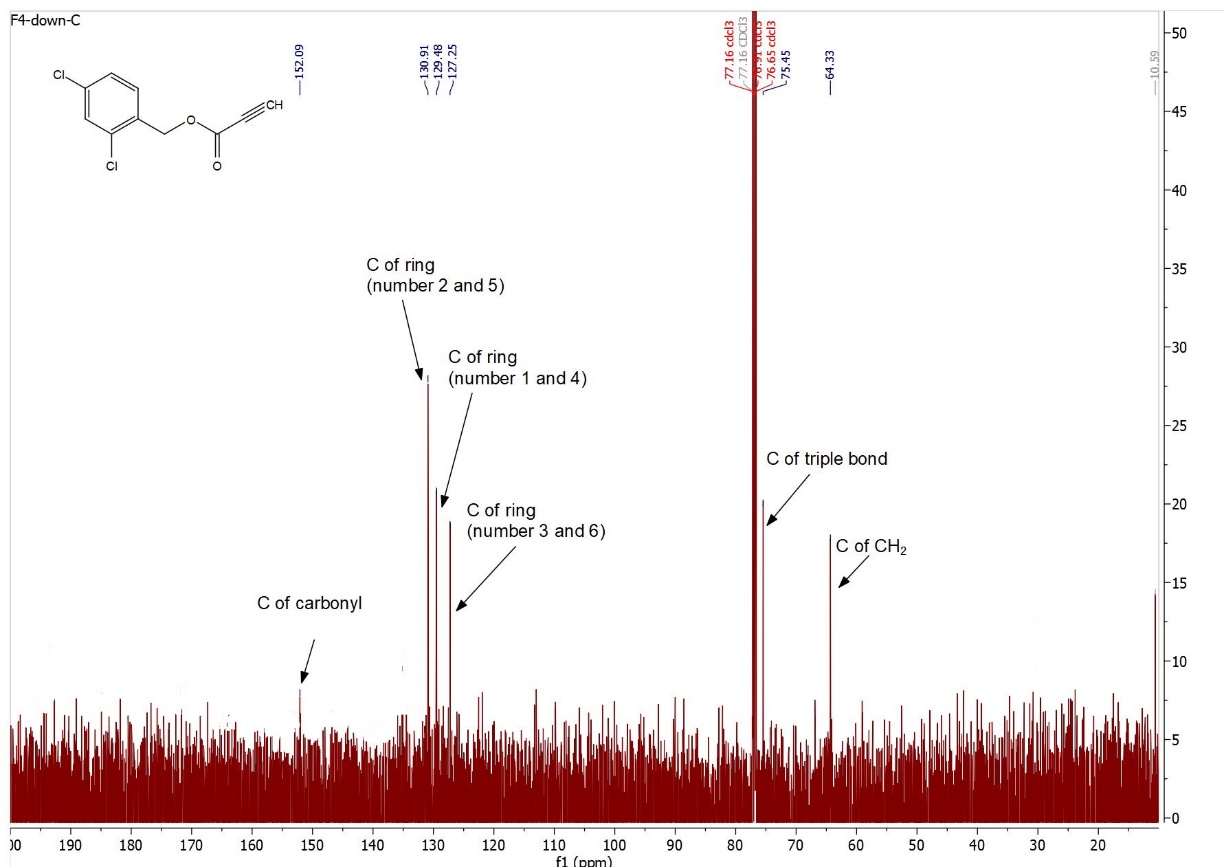


**5a:**


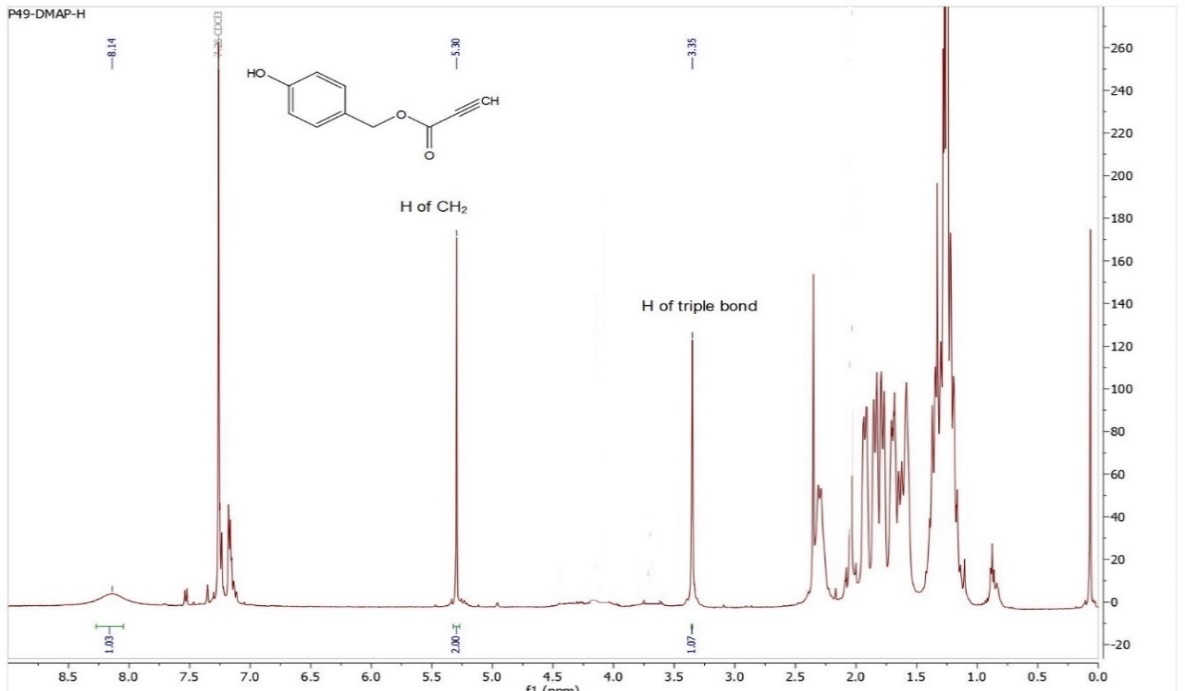


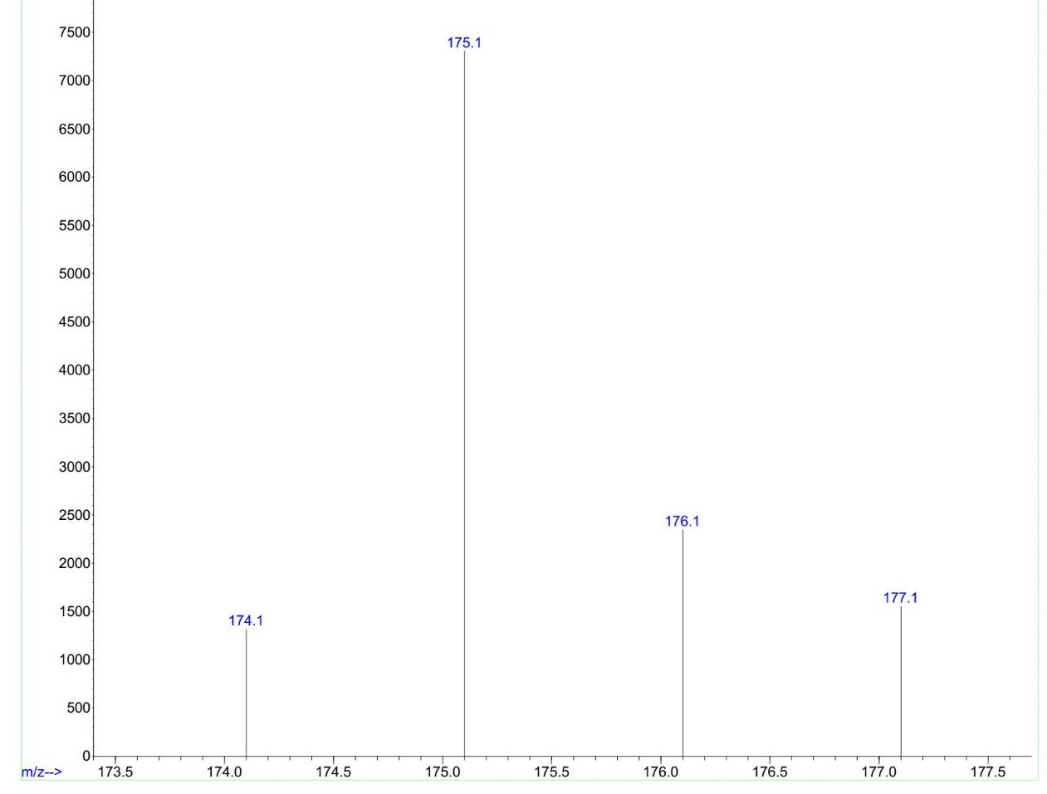


**6a:**


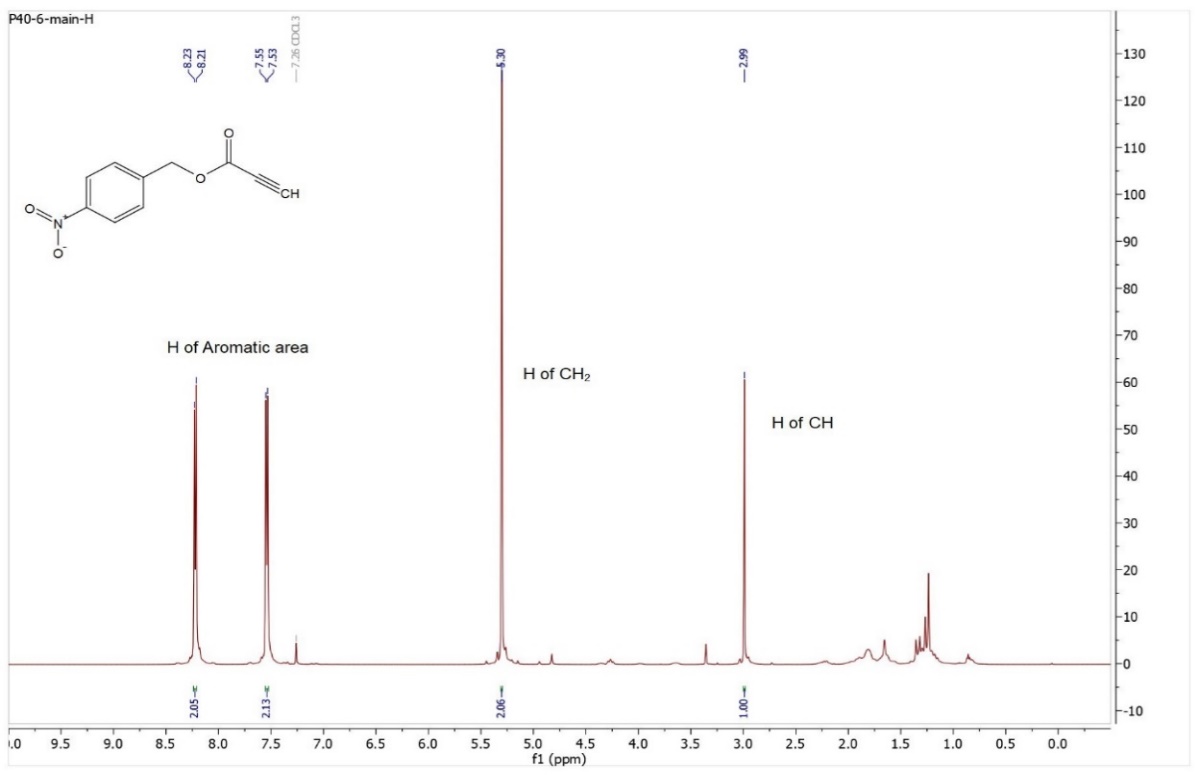


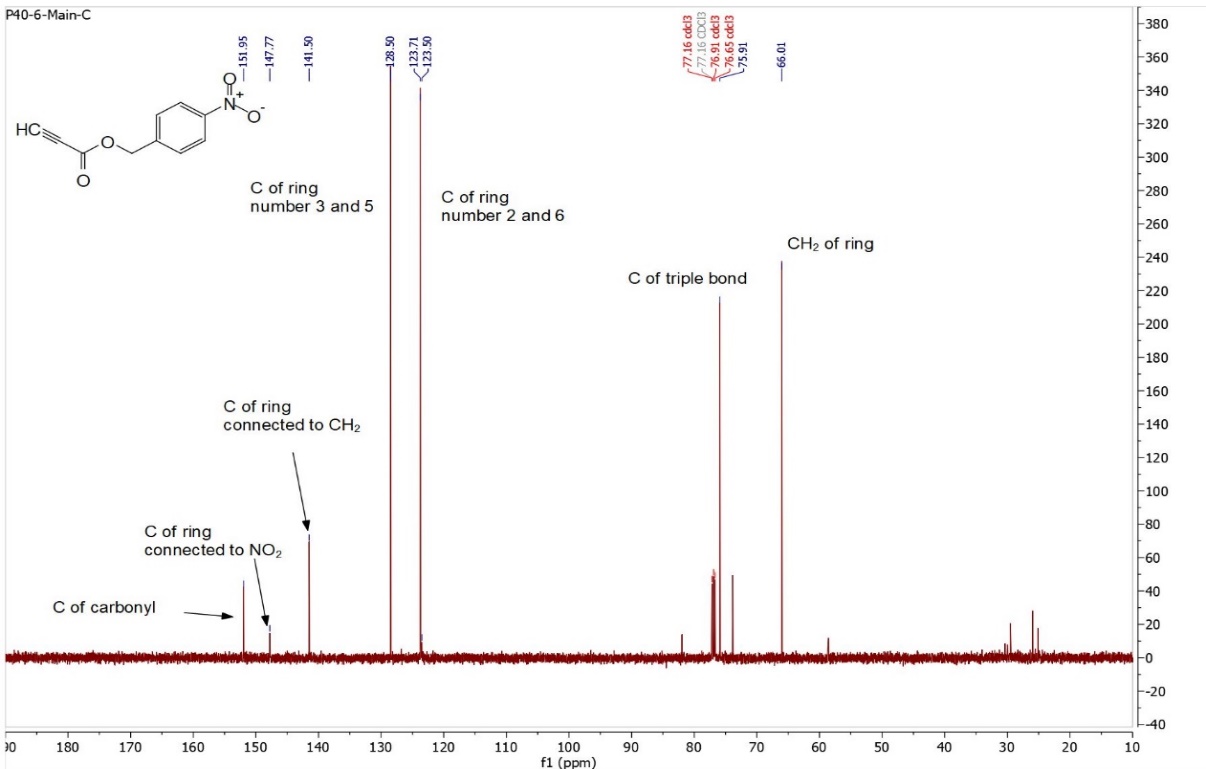


**7a:**


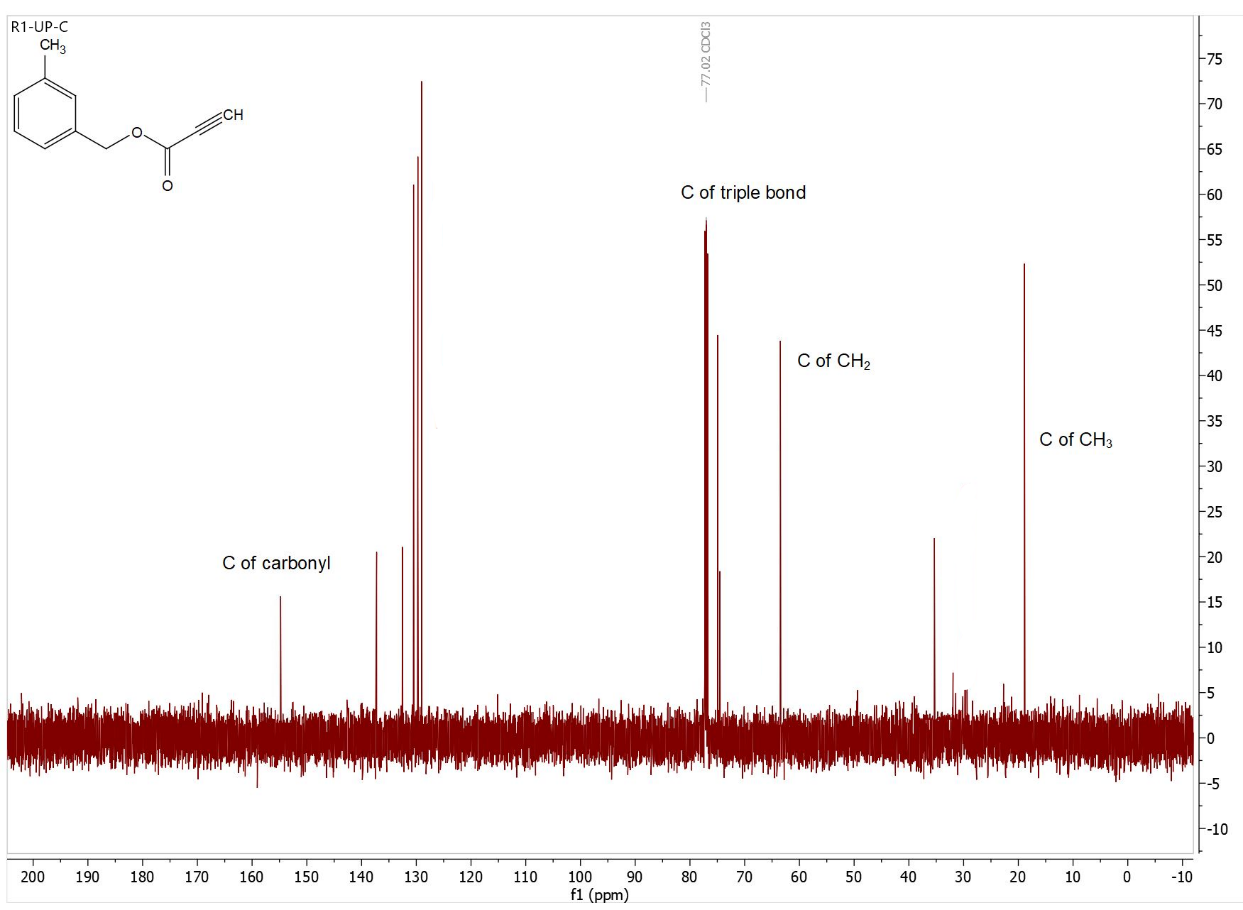


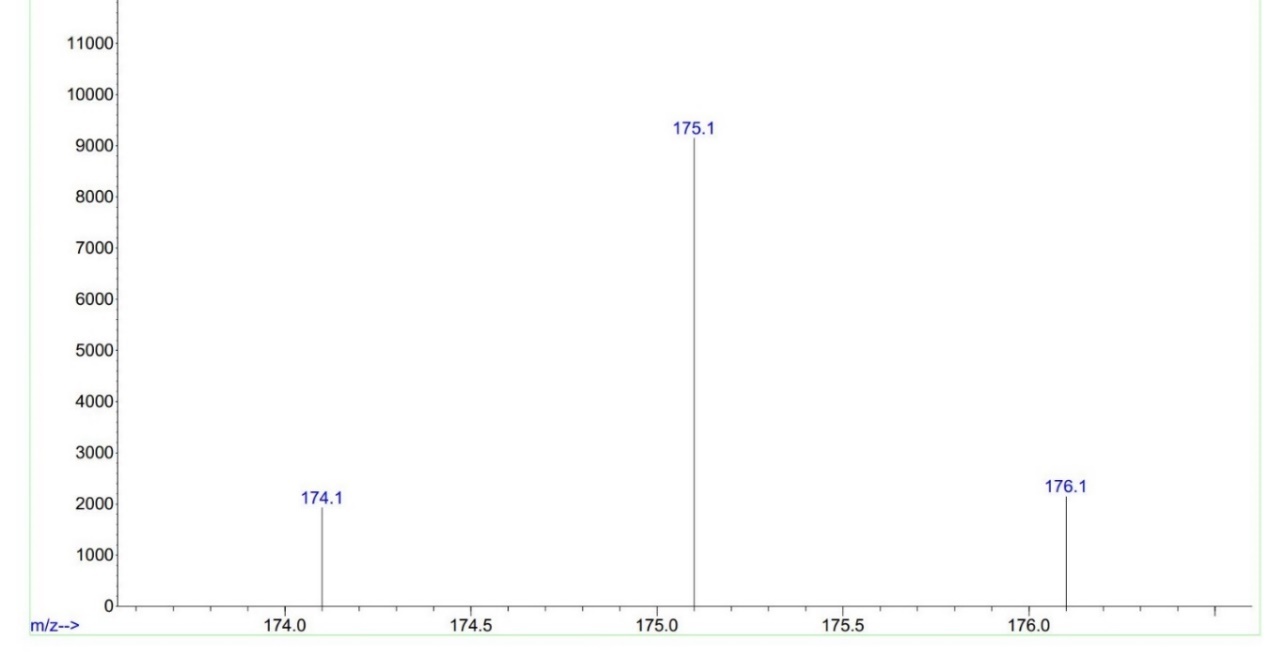


**8a:**


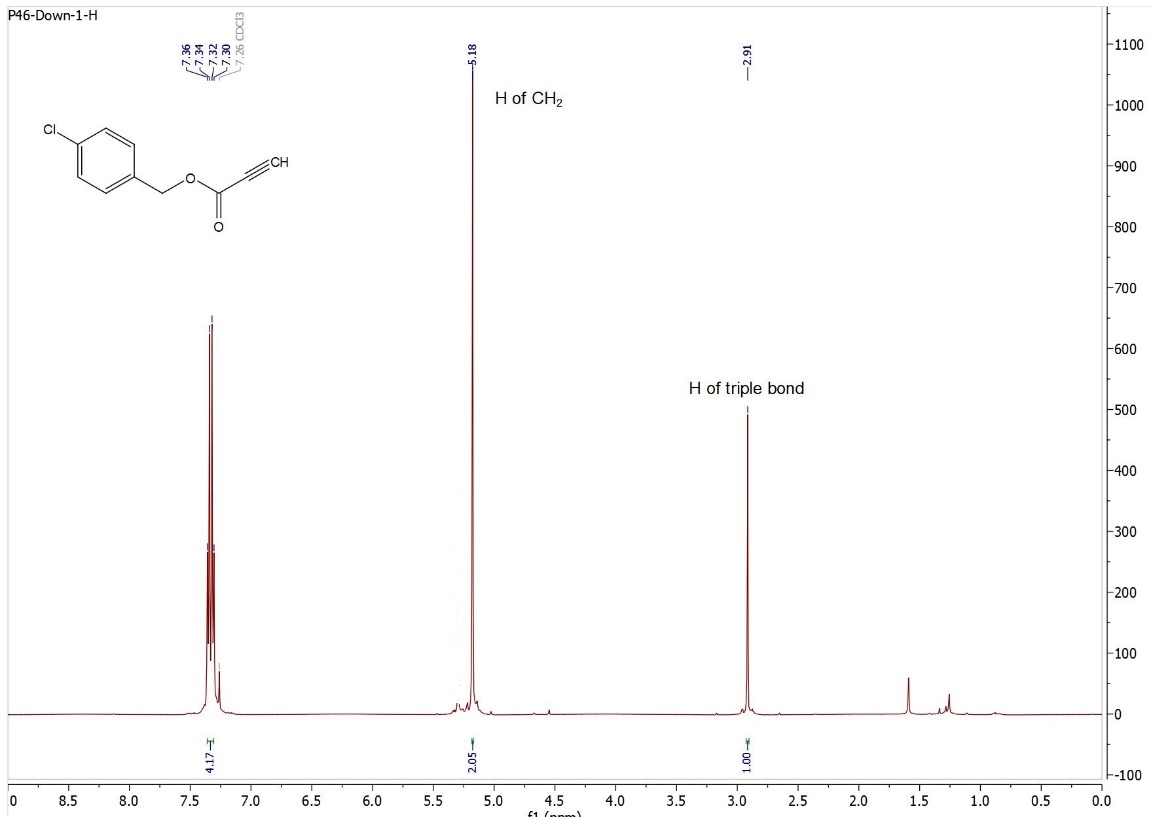


**9a:**


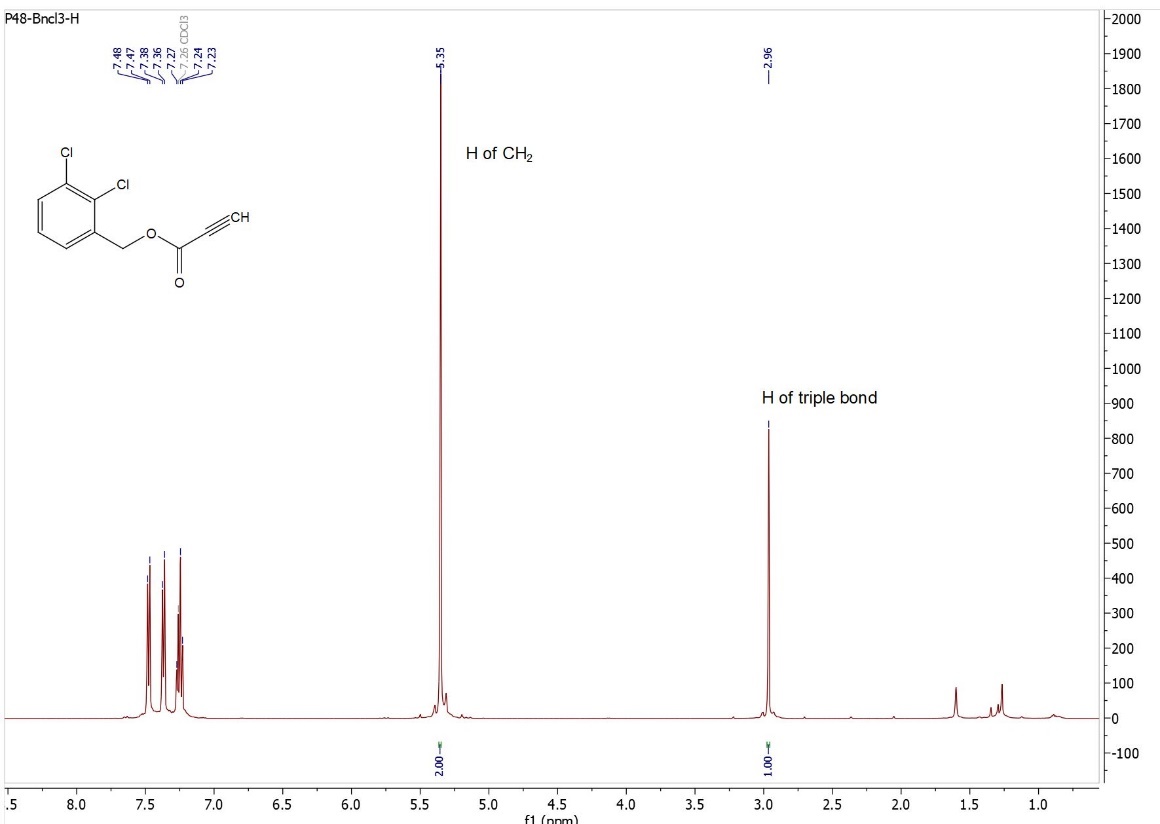


**10a:**


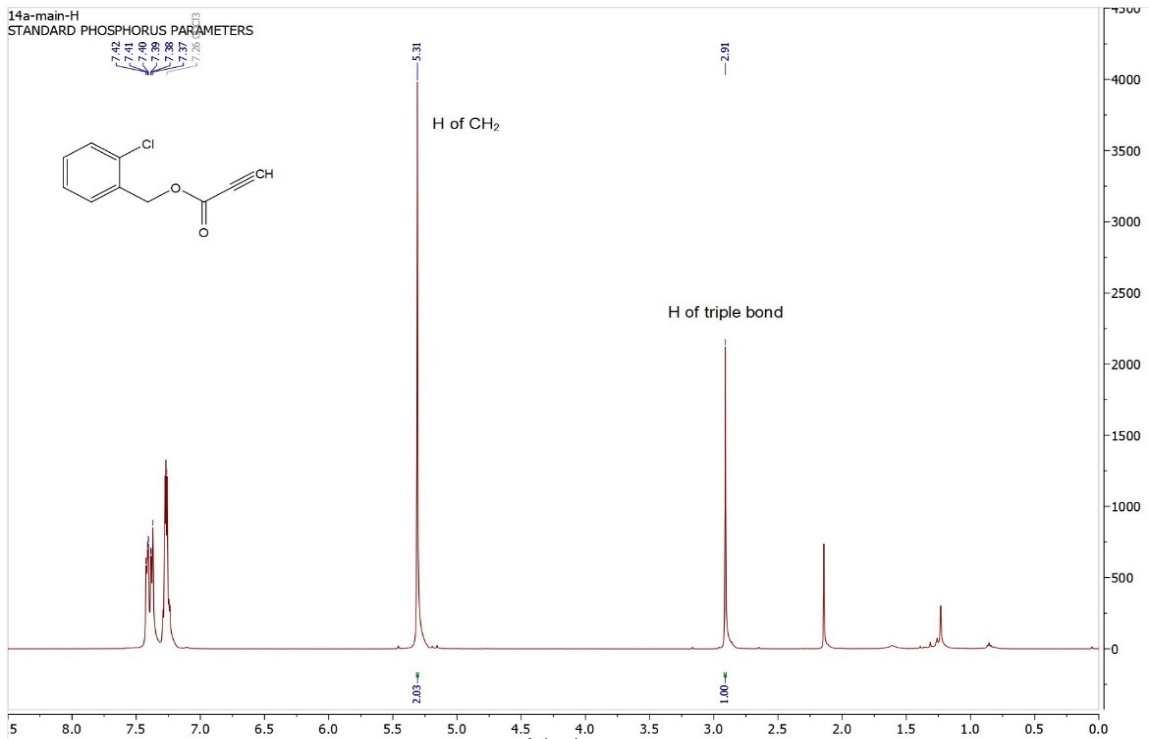


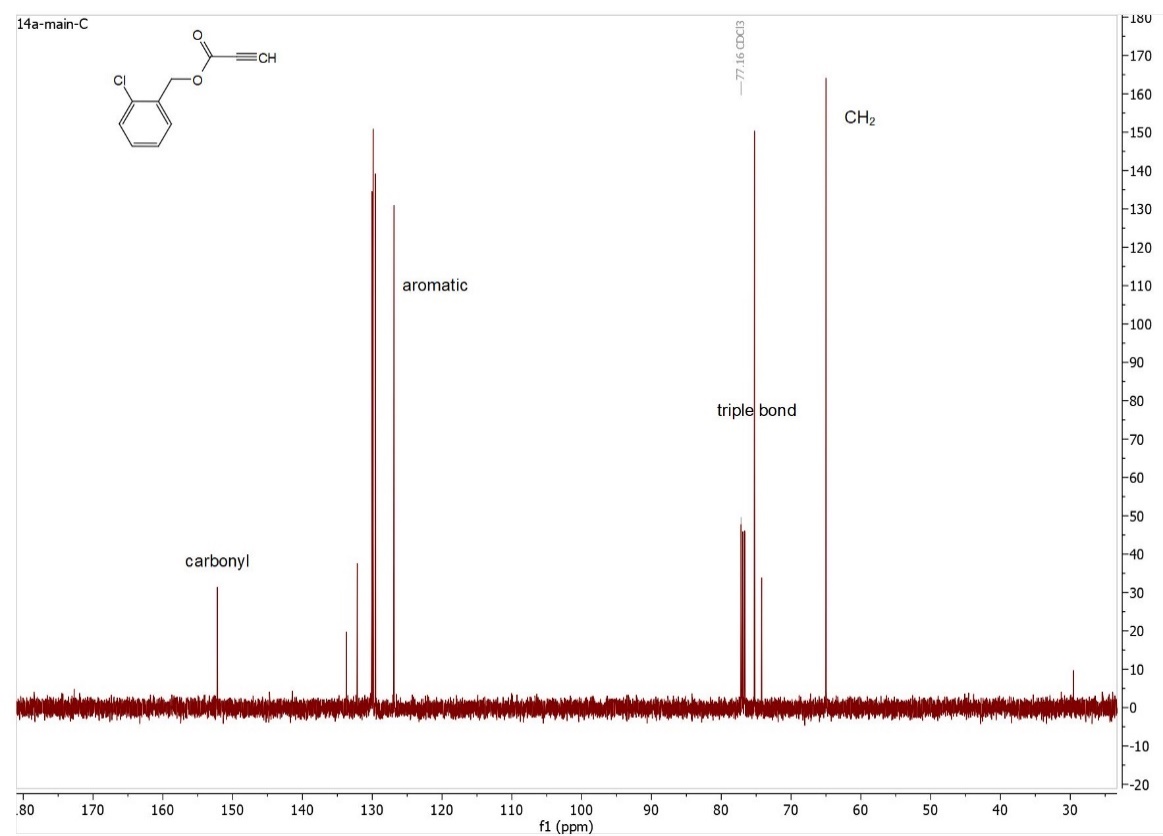


**1b:**


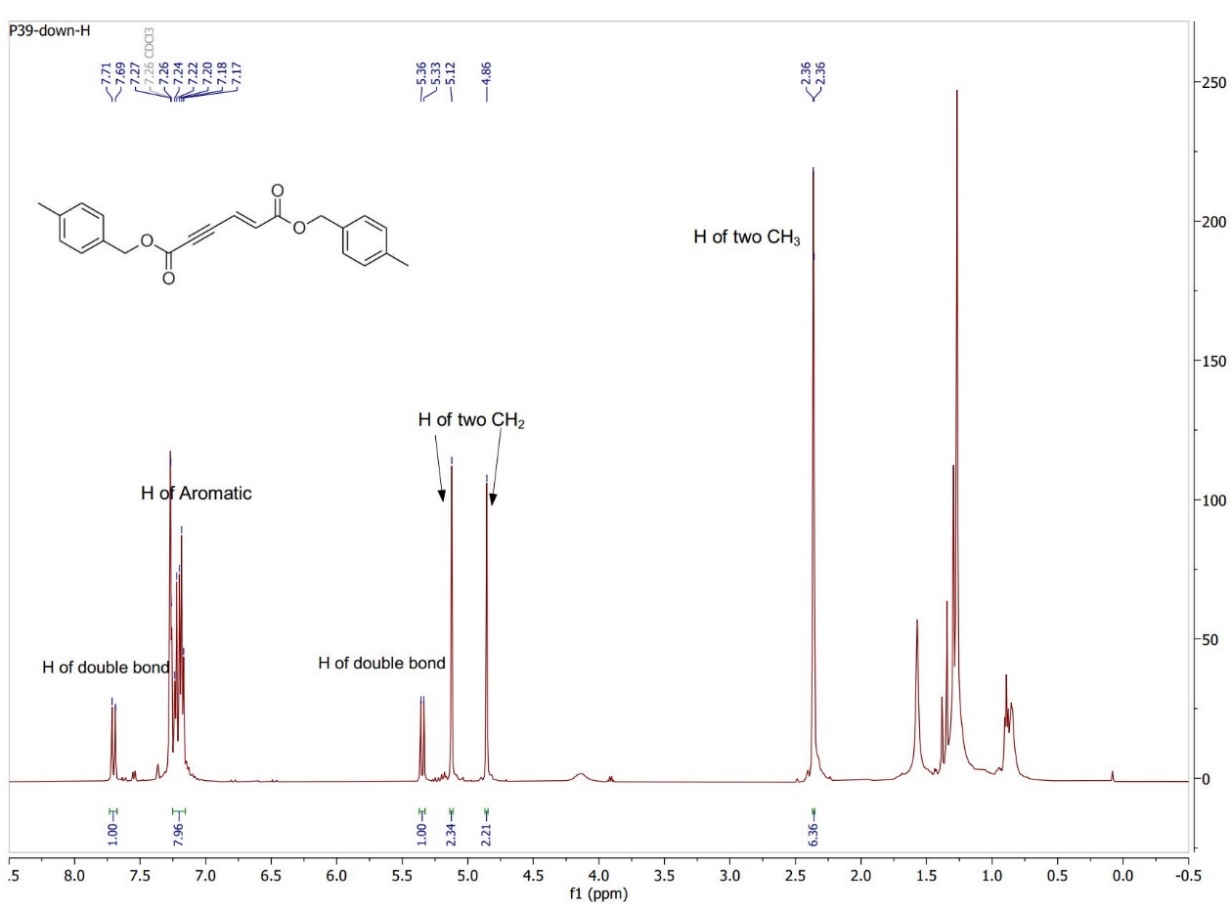


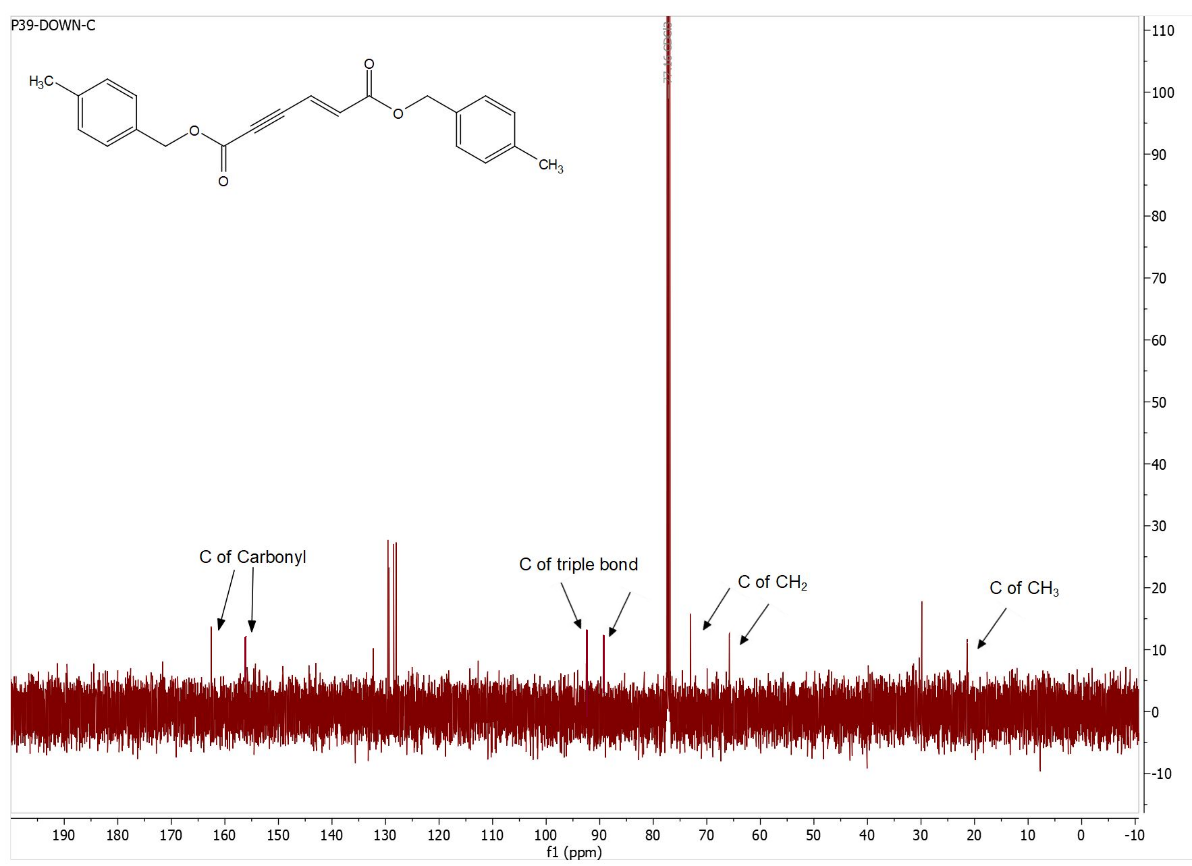


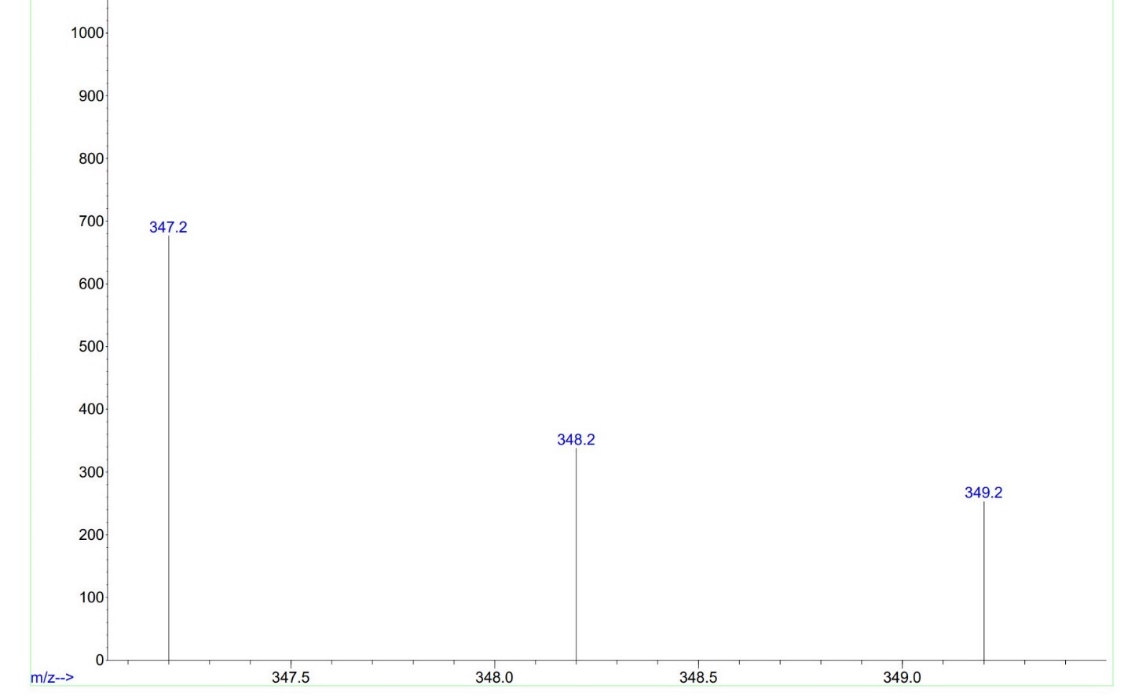


**2b:**


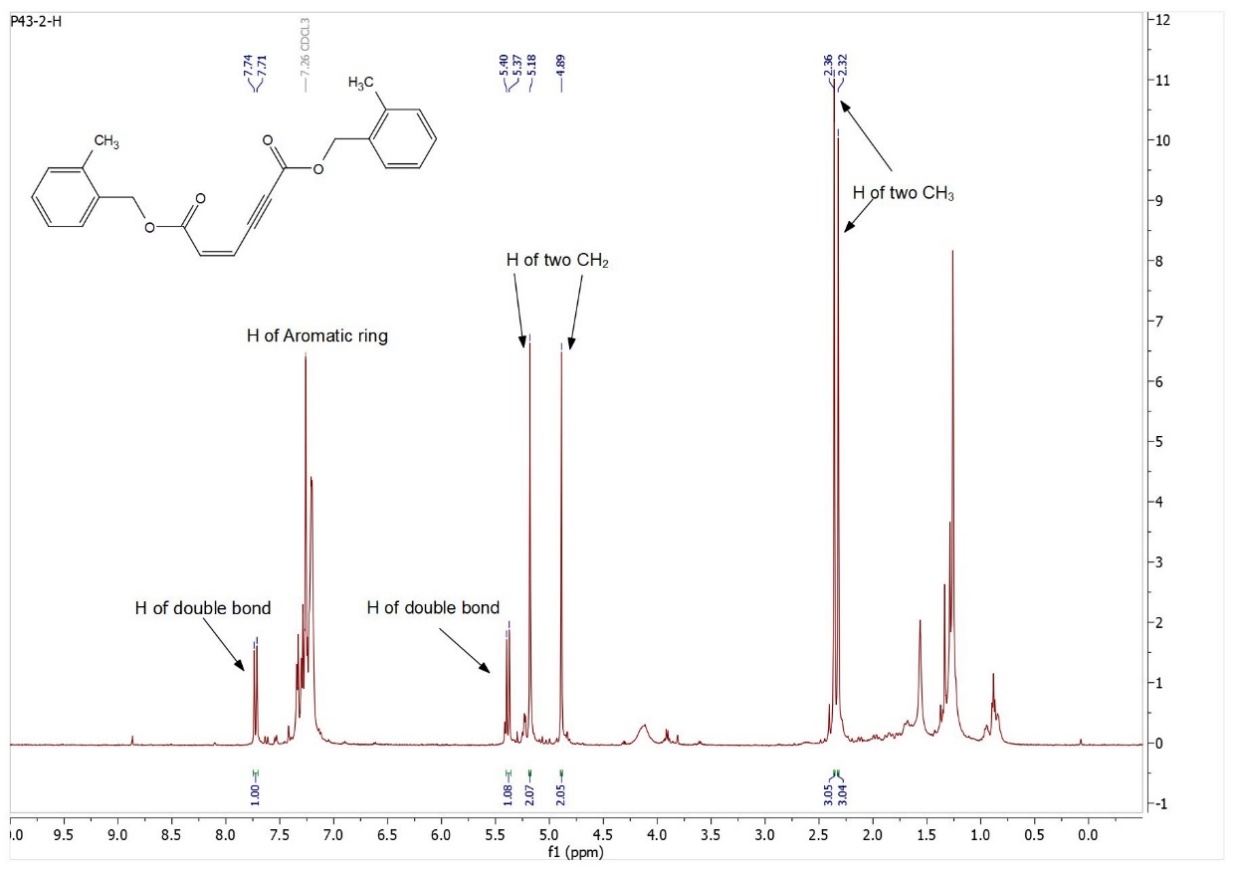


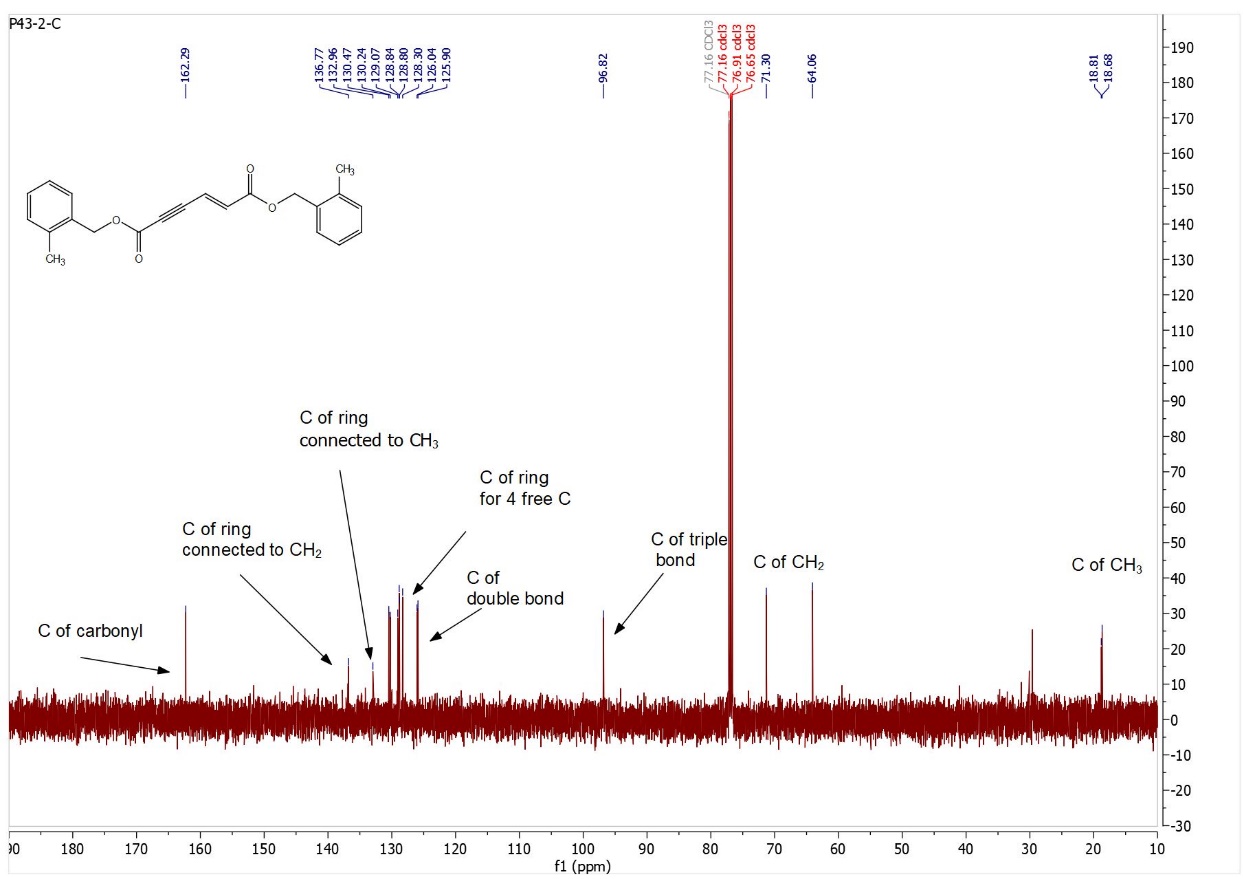


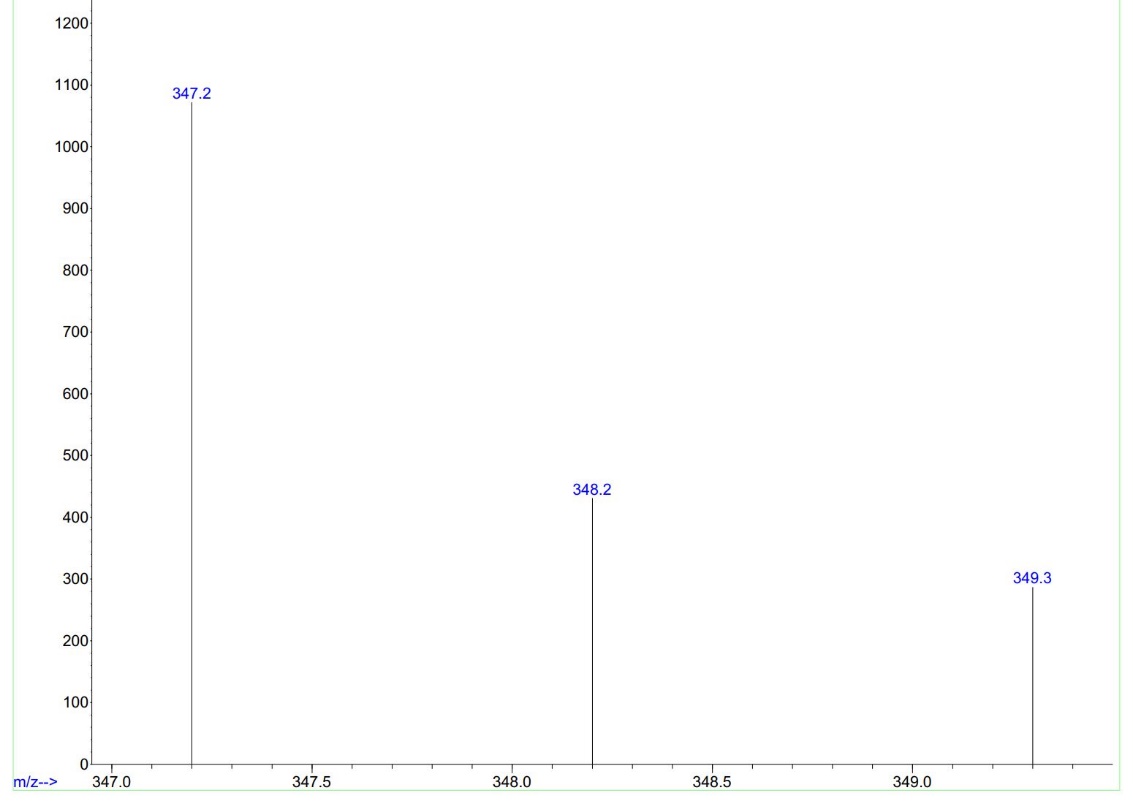


**3b:**


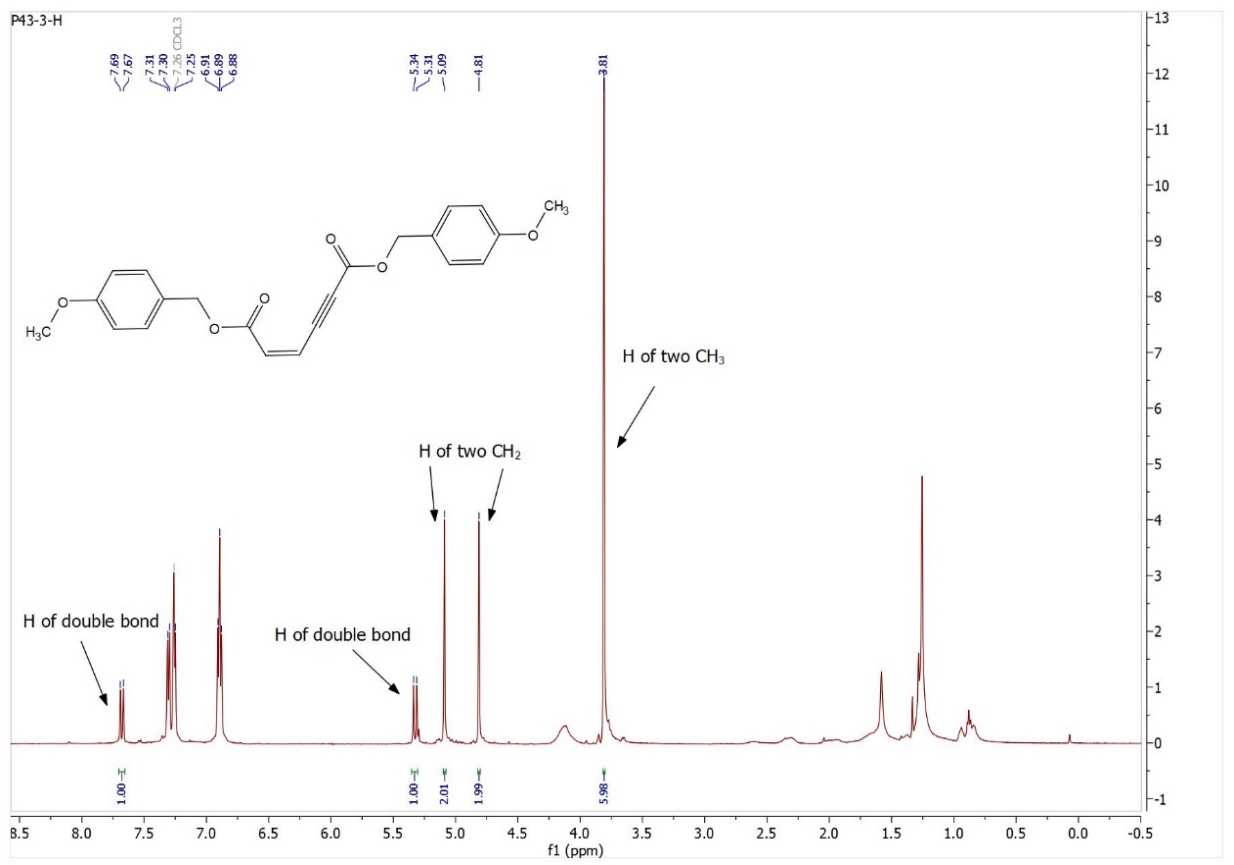


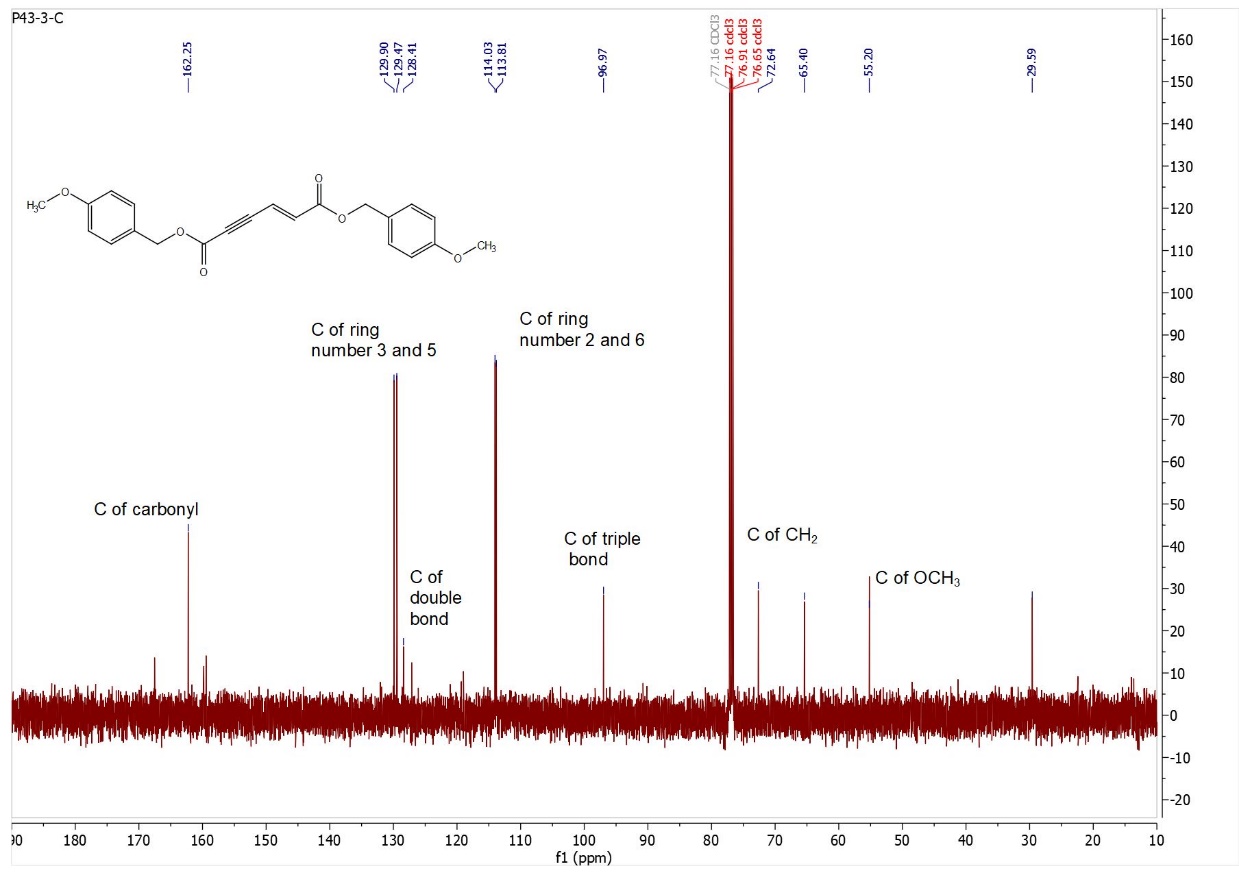


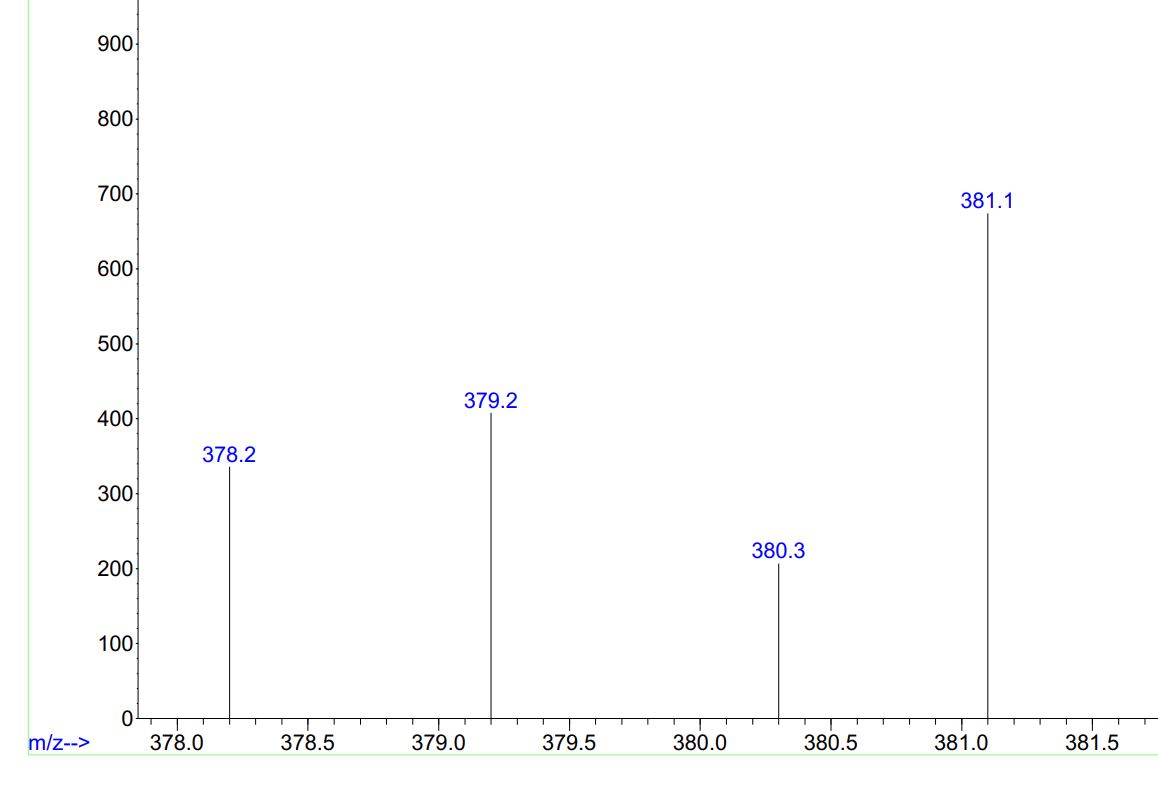


**4b:**


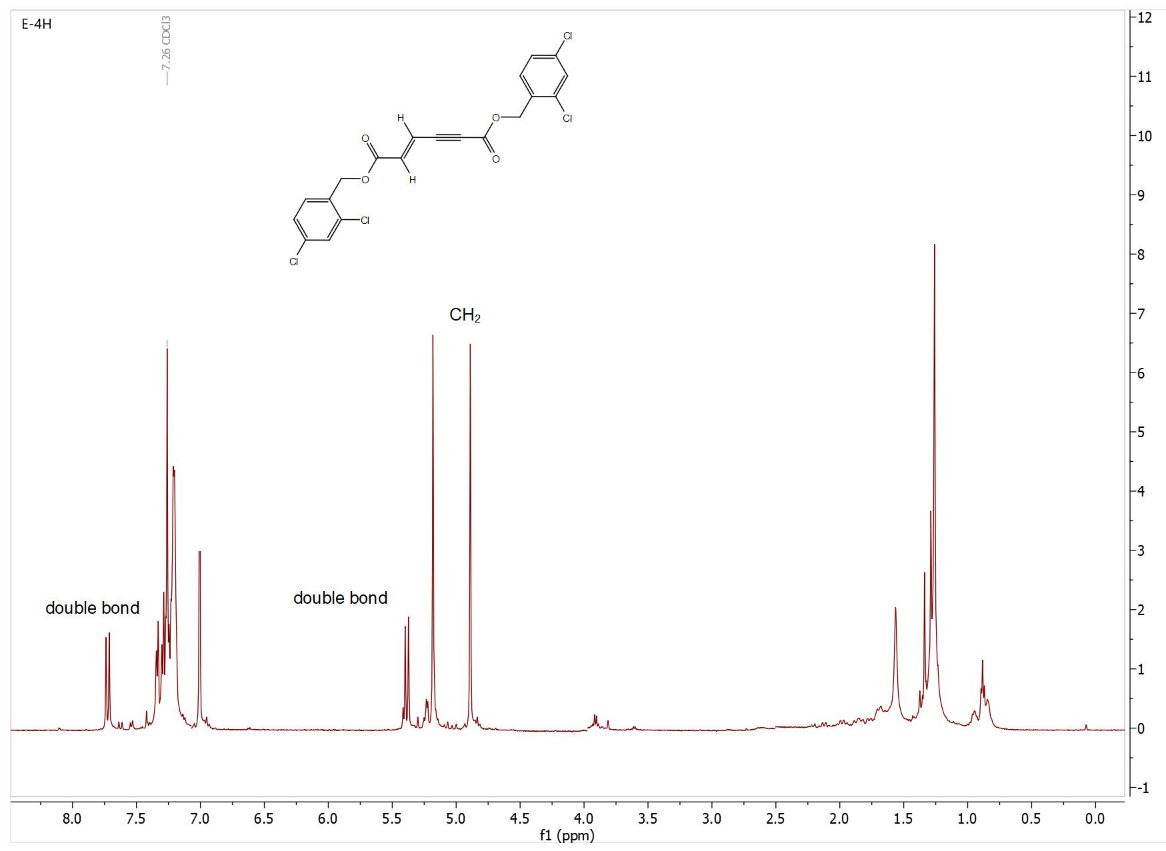


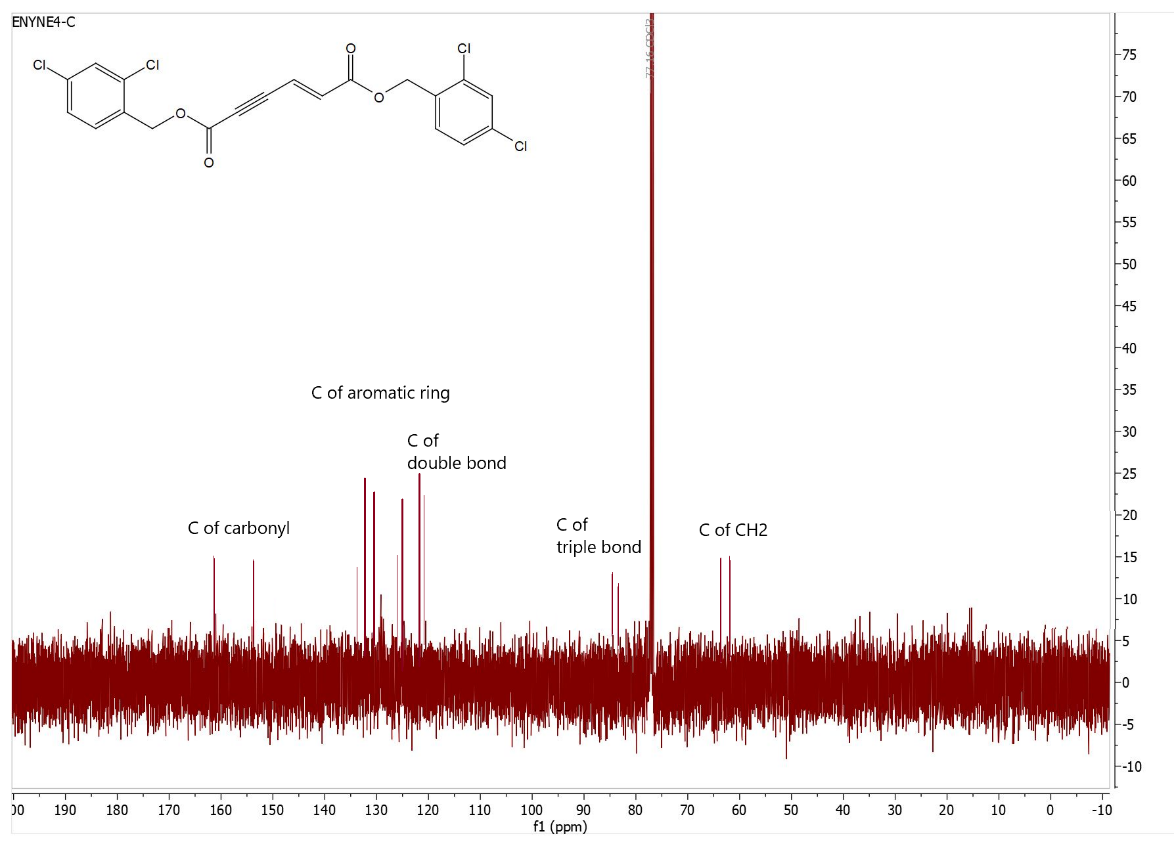


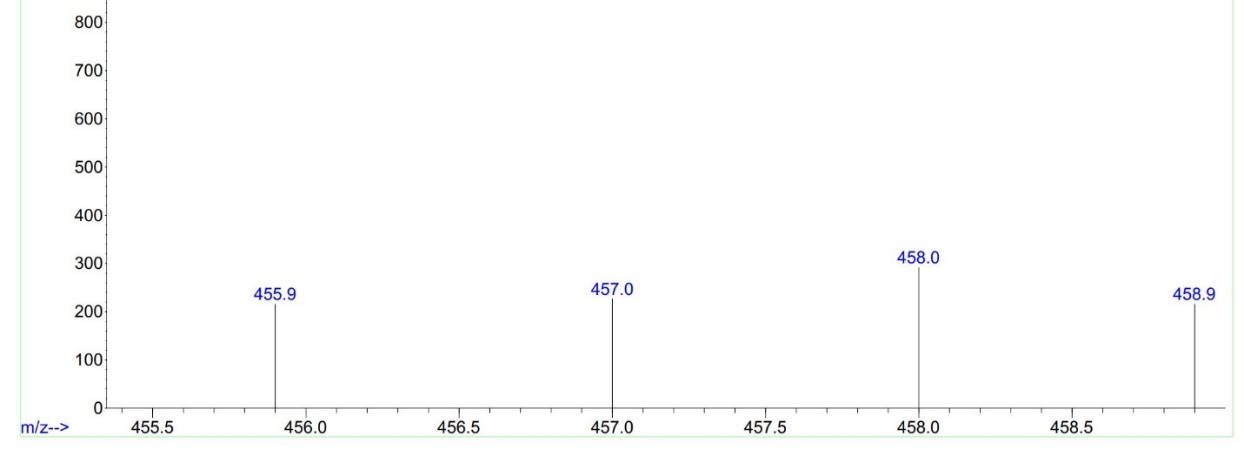


**5b:**


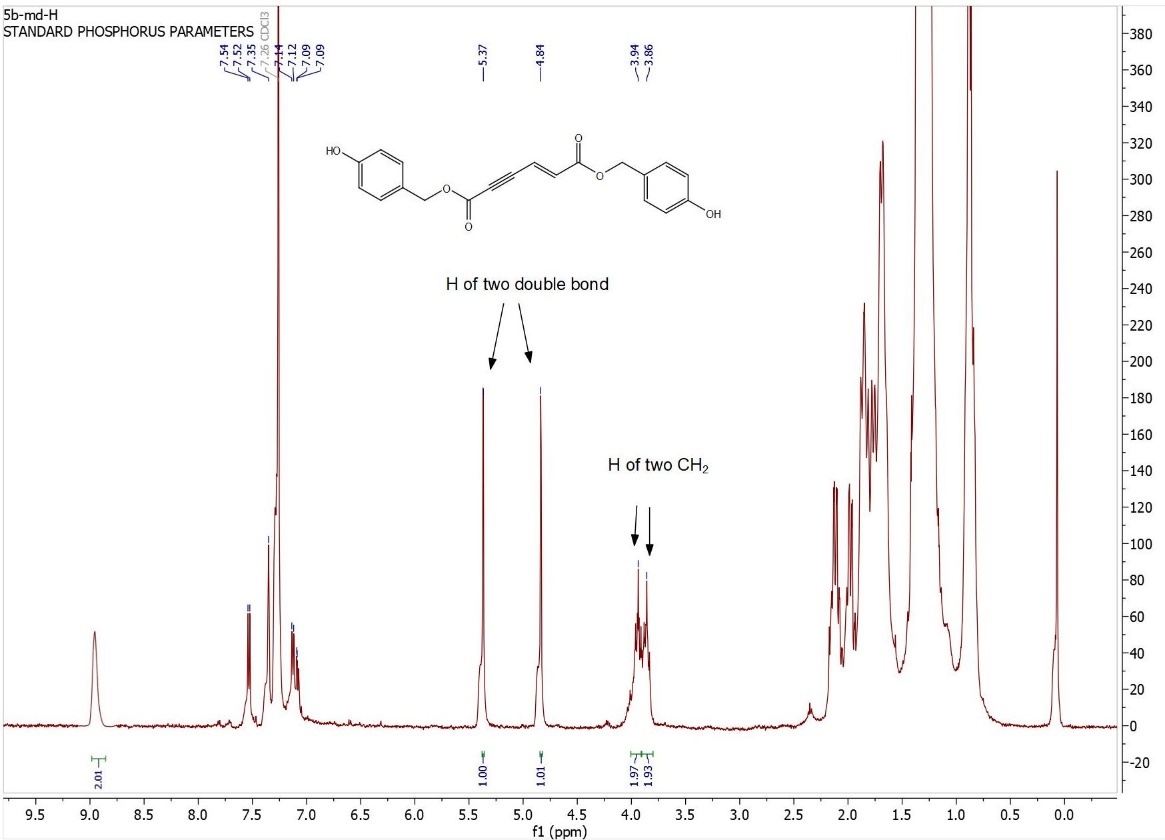


**6b:**


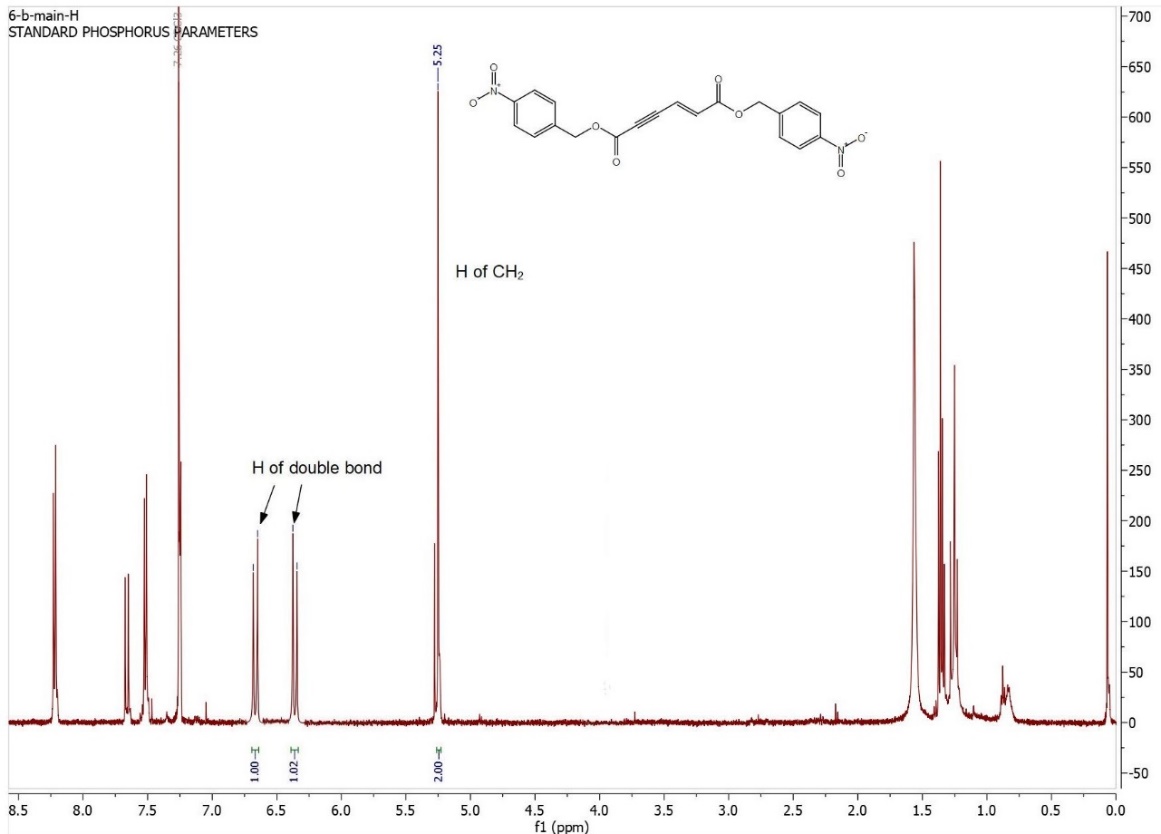


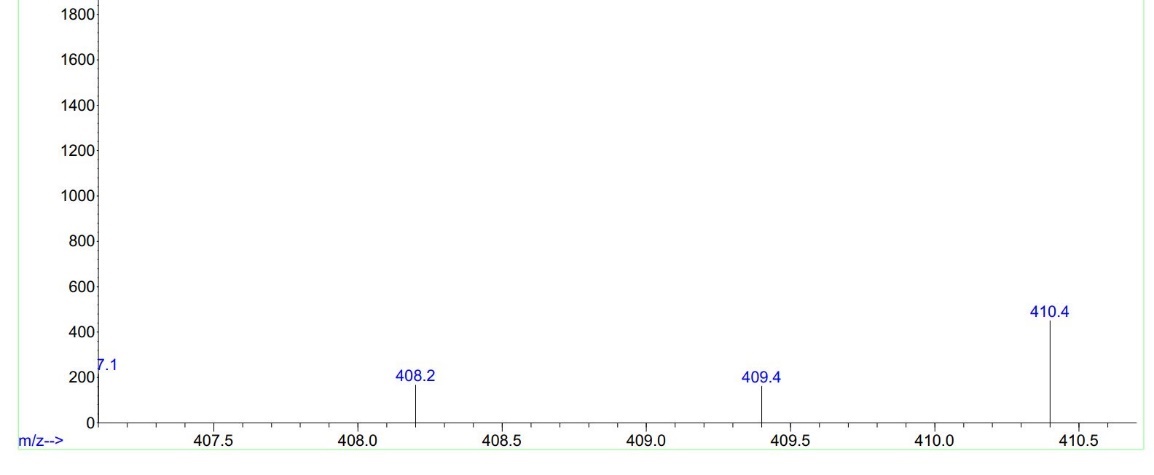


**7b:**


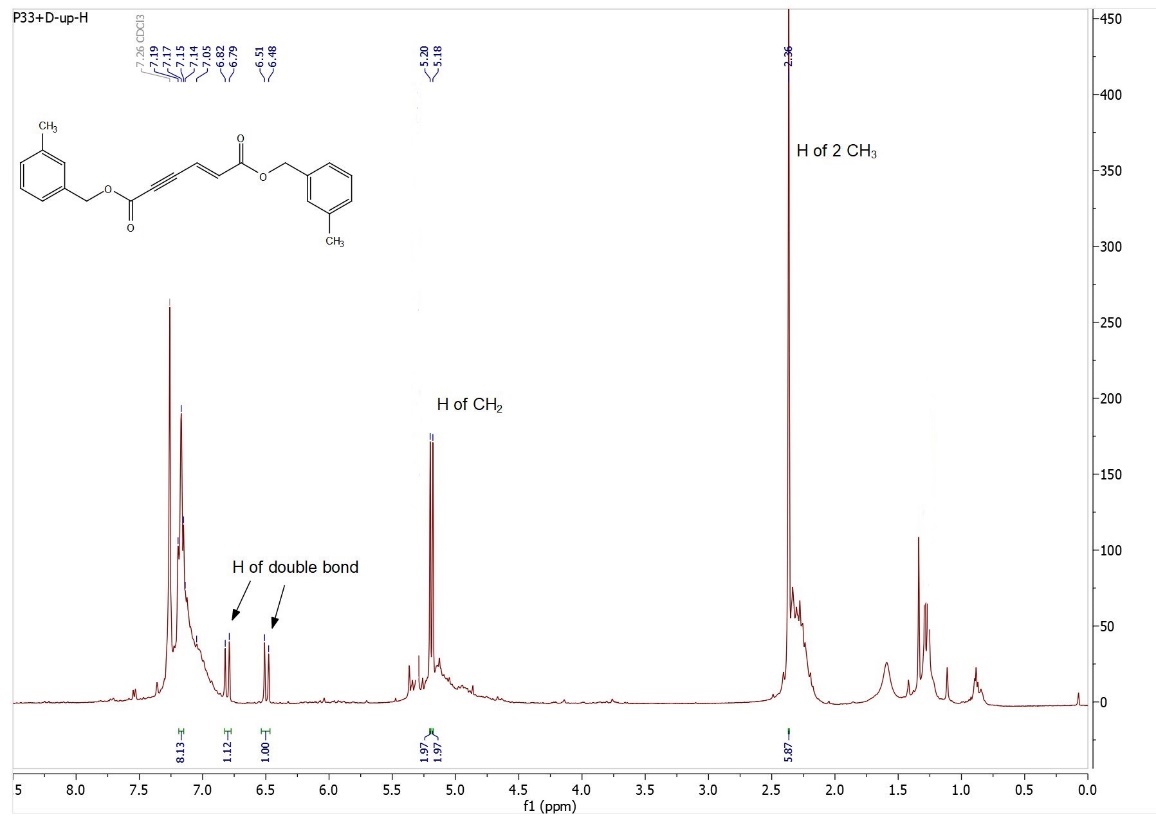


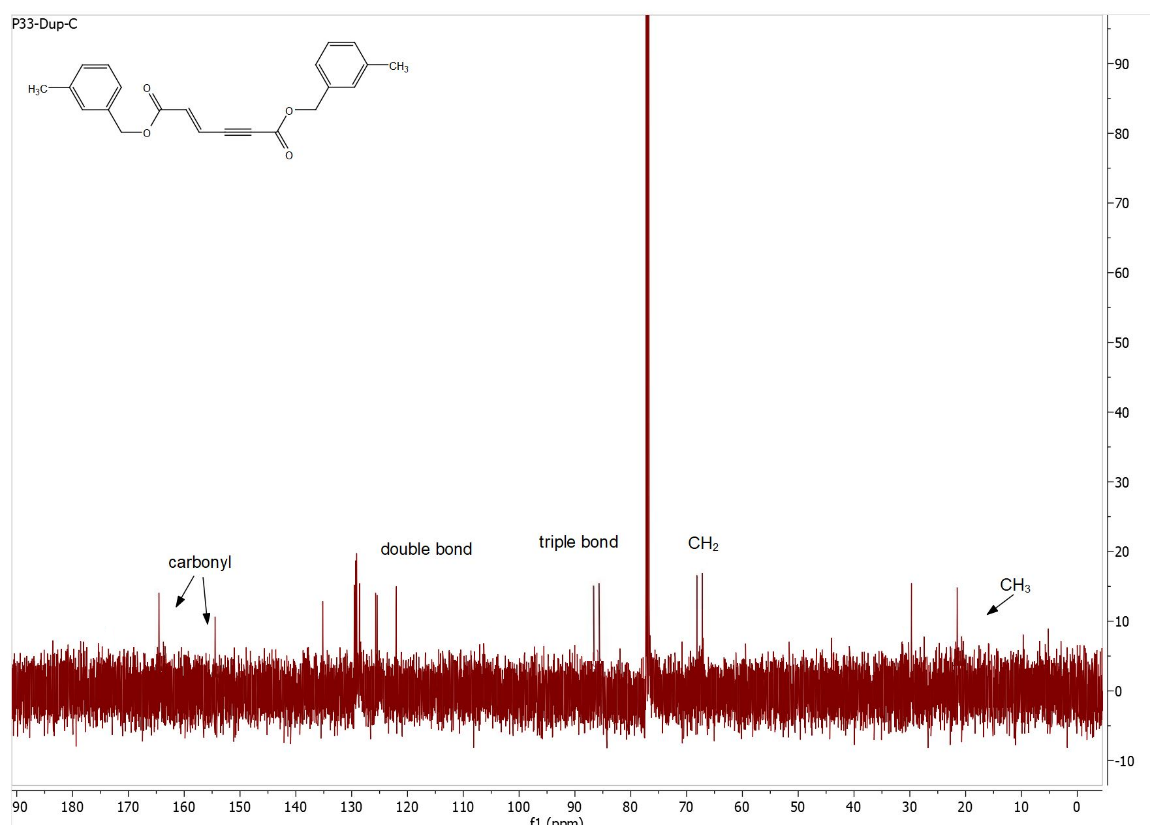


**8b:**


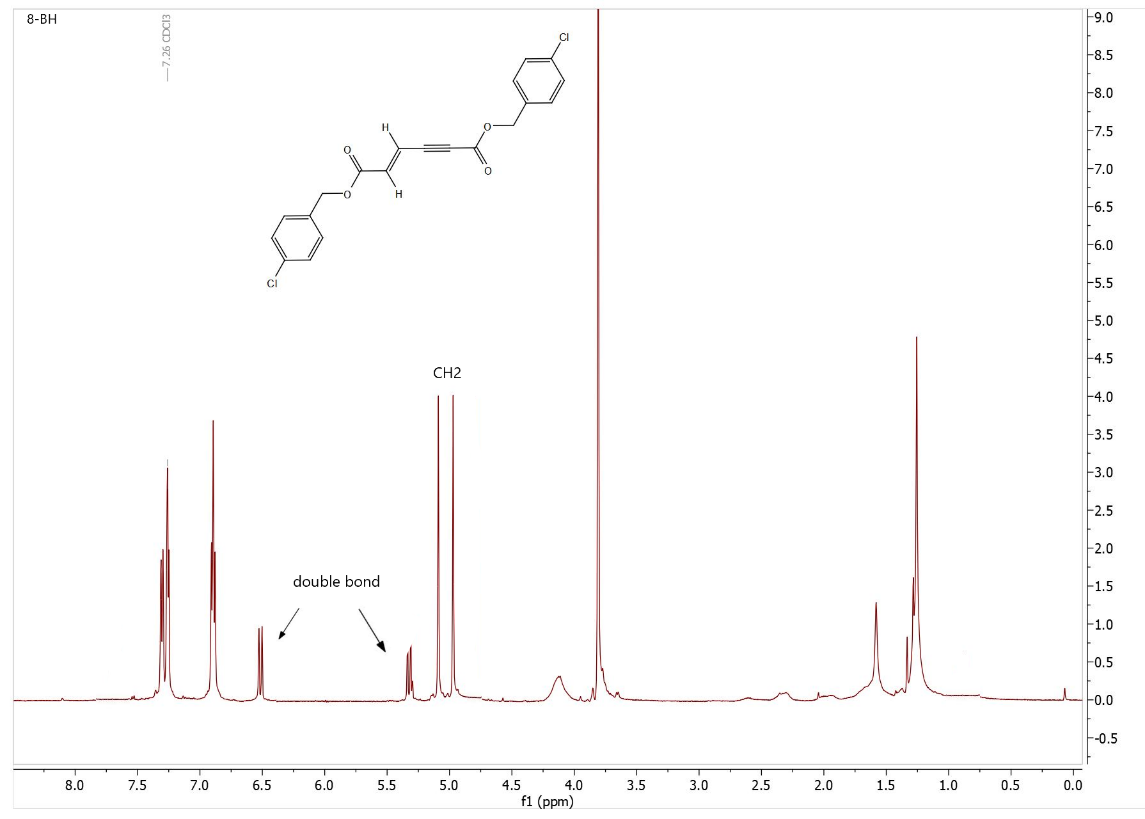


**9b:**


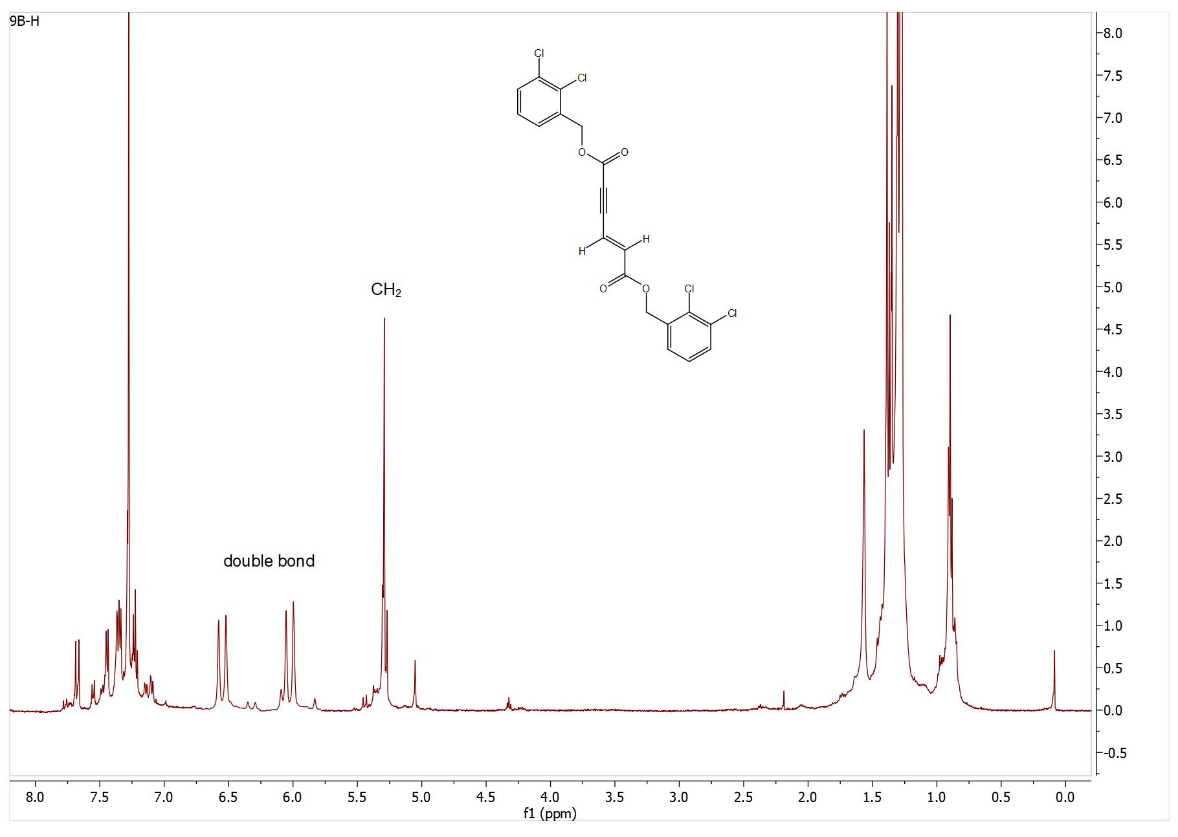


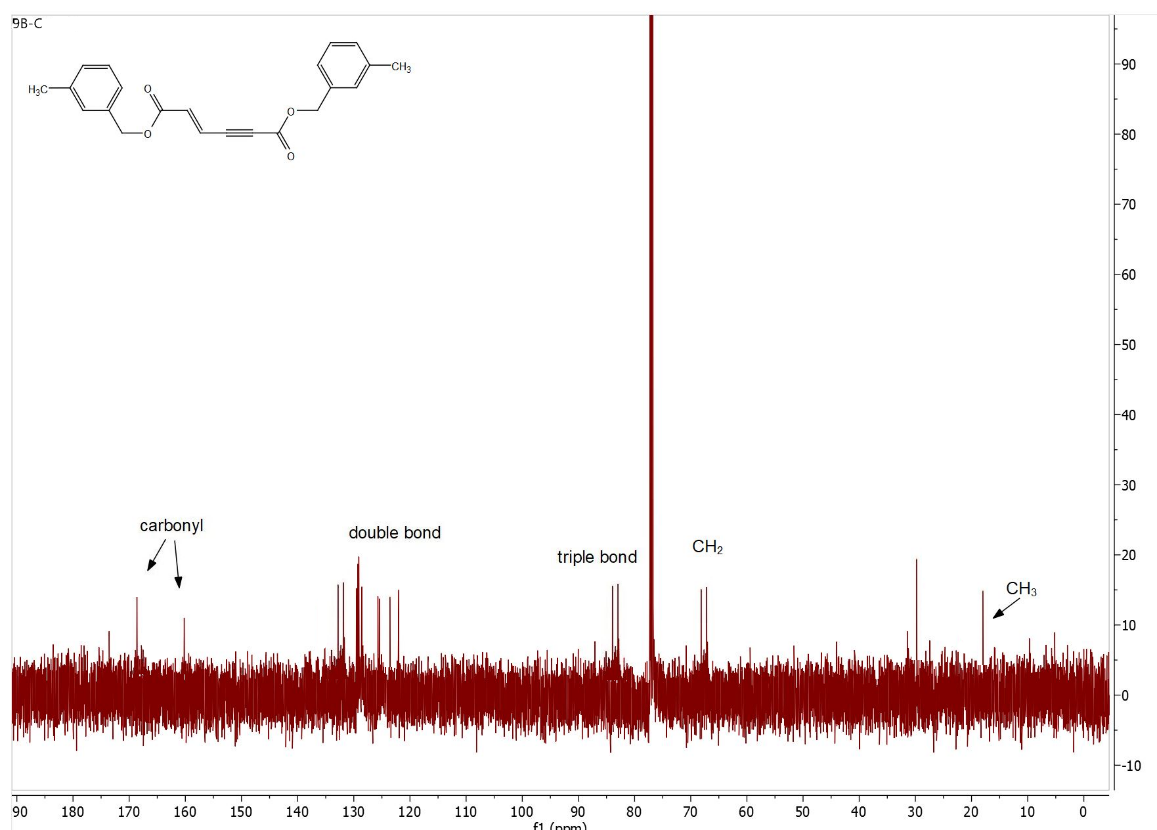


**10b:**


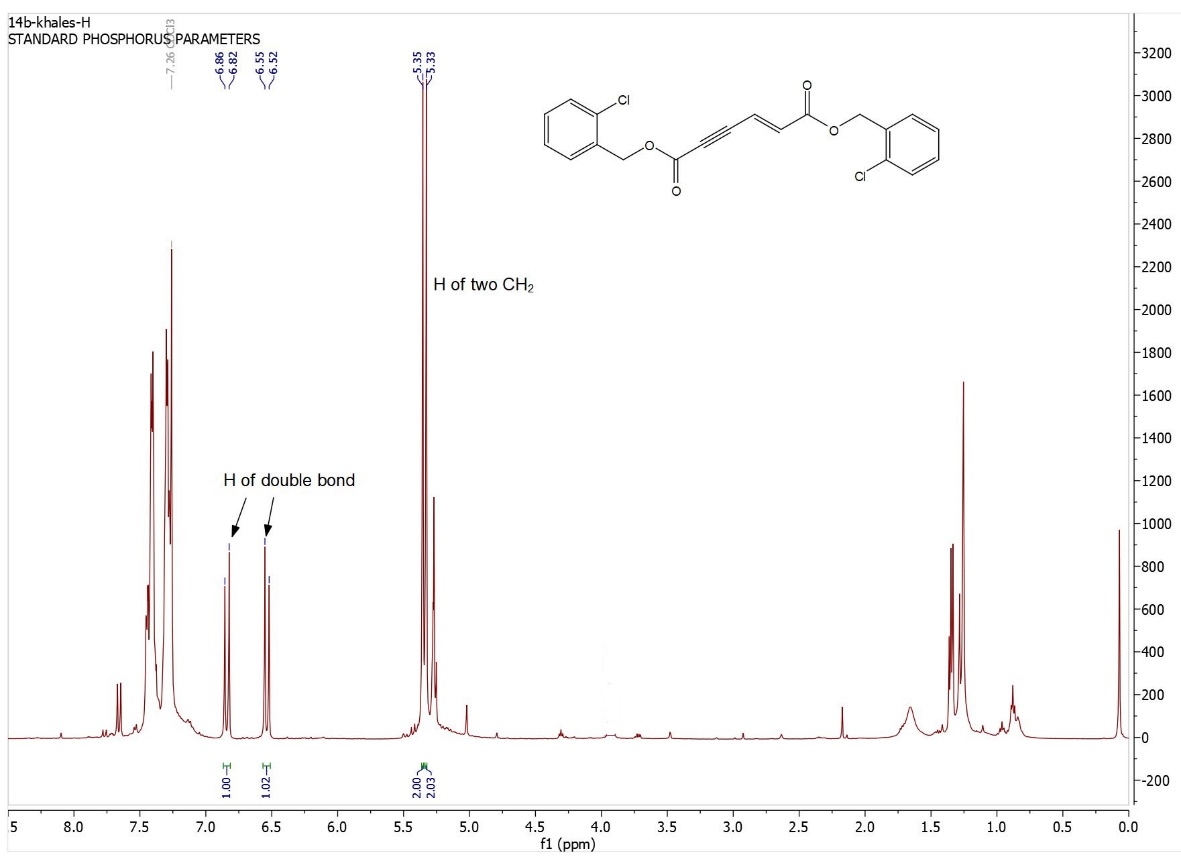


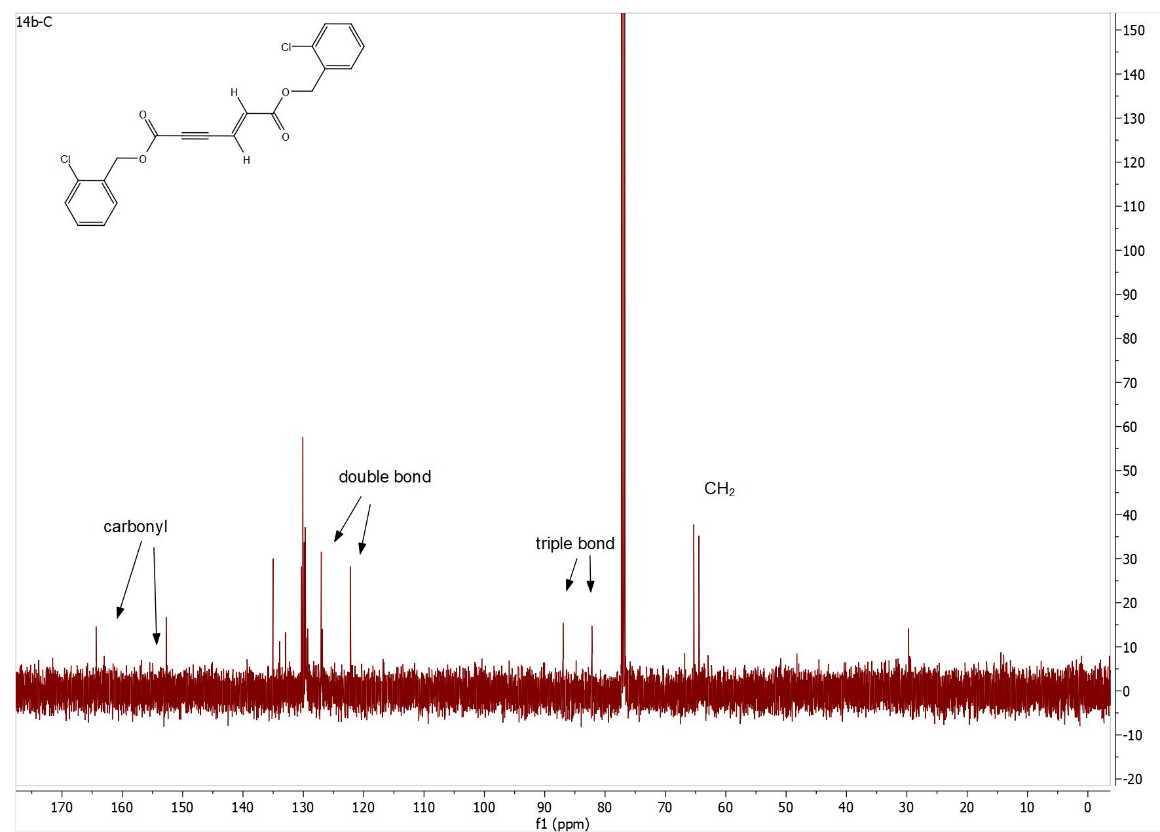


1. **Full MIC datasets (Supplementary Table S1)**

| **Sample** | ***C.albicans***  **ATCC:10231**  **MIC(μg/ml*)*** | ***S.cervisia***  **PTCC:5052**  **MIC(μg/ml)** | ***A. fumigatus***  **ATCC: 16424 MIC (µg/mL)** |
| --- | --- | --- | --- |
| 1a | 15.62 | 62.5 | 62.5 |
| 2a | 15.62 | 31.25 | 62.5 |
| 3a | 23.44 | 62.5 | 125 |
| 4a | 31.25 | 125 | 125 |
| 5a | 1000 | 500 | 250 |
| 6a | 1000 | 1000 | 500 |
| 7a | 1000 | 1000 | 125 |
| 8a | 1000 | 500 | 500 |
| 9a | 7.6 | 7.6 | 7.6 |
| 10a | 7.6 | 7.6 | 7.6 |
| 1b | 1000 | 500 | >500 |
| 2b | 250 | 31.25 | 250 |
| 3b | 1000 | 125 | 250 |
| 4b | 1000 | 125 | >500 |
| 5b | 1000 | 1000 | 250 |
| 6b | 1000 | 1000 | 250 |
| 7b | 1000 | 1000 | 250 |
| 8b | 1000 | 1000 | 500 |
| 9b | 1000 | 1000 | 125 |
| 10b | 1000 | 1000 | 125 |
| Terbinafine | 25 | 10 | <7.6 |
| DMSO | 10% V/V | 10% | 10% |

1. **Full docking docking outcomes (in kcal/mol) (Supplementary Table S2)**

| number | *Docking results* | Chemical Structure | IUPAC Name | Formula | M.W |
| --- | --- | --- | --- | --- | --- |
| 5b | -12.1 | 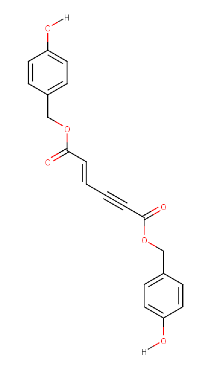 | 1,6-bis[(4-hydroxyphenyl)methyl] (2E)-hex-2-en-4-ynedioate | C_20_H_18_O_8_ | 352.34 |
| 3b | -11.1 | 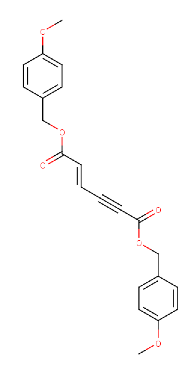 | 1,6-bis[(4-methoxyphenyl)methyl] (2E)-hex-2-en-4-ynedioate | C_22_H_20_O_6_ | 380.4 |
| 2b | -10.3 | 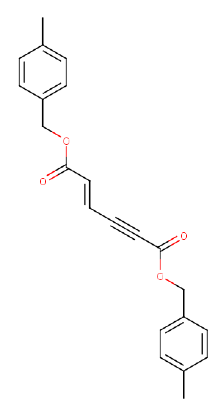 | 1,6-bis[(4-methylphenyl)methyl] (2E)-hex-2-en-4-ynedioate | C_22_H_20_O_4_ | 348.4 |
| 7b | -10.2 | 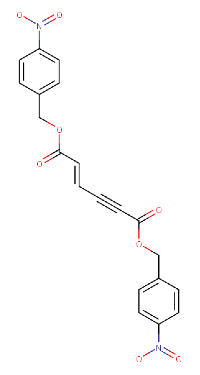 | bis(4-(dihydroxyamino)benzyl) (E)-hex-2-en-4-ynedioate | C_20_H_18_N_2_O_8_ | 414.37 |
| 4b | -10.1 | 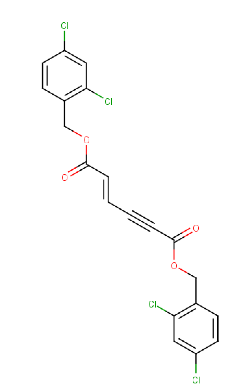 | 1,6-bis[(2,4-dichlorophenyl)methyl] (2E)-hex-2-en-4-ynedioate | C_20_H_12_Cl_4_O_4_ | 458.11 |
| 1b | -10 | 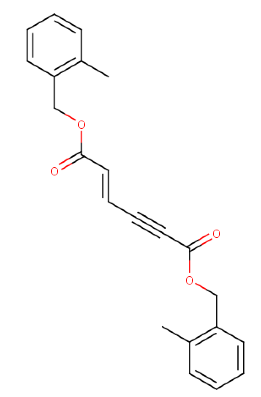 | 1,6-bis[(2-methylphenyl)methyl] (2E)-hex-2-en-4-ynedioate | C_22_H_20_O_4_ | 348.4 |
| 8b | -10 | 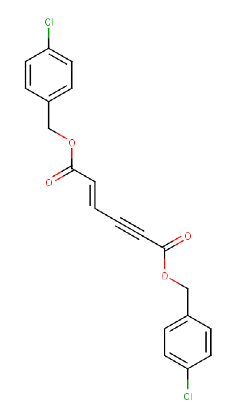 | 1,6-bis[(4-chlorophenyl)methyl] (2E)-hex-2-en-4-ynedioate | C_20_H_14_Cl_2_O_4_ | 389.23 |
| 8 | -9.9 | 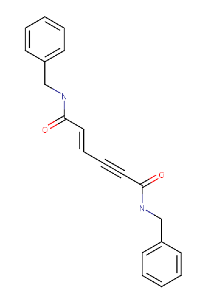 | (2E)-N,N'-dibenzylhex-2-en-4-ynediamide | C_20_H_18_N_2_O_2_ | 318.38 |
| 10b | -9.9 | 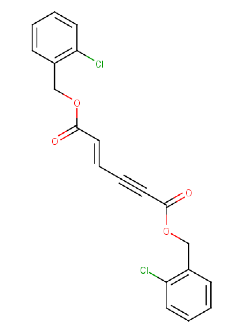 | 1,6-bis[(2-chlorophenyl)methyl] (2E)-hex-2-en-4-ynedioate | C_20_H_14_Cl_2_O_4_ | 389.23 |
| 10 | -9.3 | 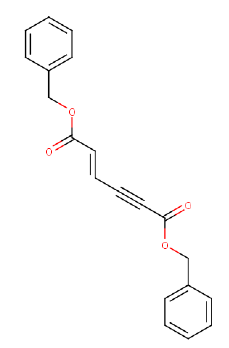 | 1,6-dibenzyl (2E)-hex-2-en-4-ynedioate | C_20_H_16_O_4_ | 320.34 |
| 12 | -9.3 | 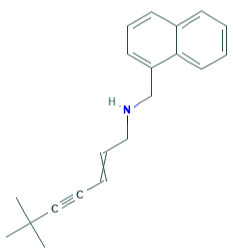 | 6,6-dimethyl-*N*-(naphthalen-1-ylmethyl)hept-2-en-4-yn-1-amine | [C_20_H_23_N](https://pubchem.ncbi.nlm.nih.gov/#query=C20H23N) | 277.4 |
| Terbinafine | -9.3 | 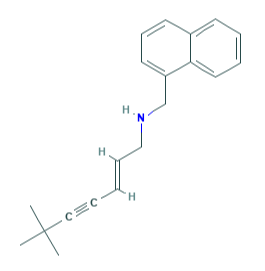 | (*E*)-6,6-dimethyl-*N*-(naphthalen-1-ylmethyl)hept-2-en-4-yn-1-amine | [C_20_H_23_N](https://pubchem.ncbi.nlm.nih.gov/#query=C20H23N) | 277.4 |
